# Supplementary material for: Effects of brand and brand trust on initial trust in fully automated driving system
Source: PLoS One. 2023 May 4;18(5):e0284654. doi: 10.1371/journal.pone.0284654 (PMC10159113; doi:10.1371/journal.pone.0284654)
Supplement: S1 File — (PDF) [file pone.0284654.s001.pdf]

SAMPLEID	maker_name	maker_umber	TB1	TB8	TB2	TB3	TB7	TB6	TB5	TB4	TA1	TA2	TA3	TA4	GENDER	AGE
1	Brand1	1	5	6	6	6	5	7	6	6	5	5	5	5	1	60
1	Brand2	2	5	5	5	5	6	5	5	5	5	5	4	5	1	60
1	Brand3	3	3	3	2	3	3	3	4	3	3	3	3	3	1	60
1	Brand4	4	4	4	4	4	4	4	4	3	5	4	4	4	1	60
1	Brand5	5	5	5	5	5	5	4	5	4	4	4	4	5	1	60
1	Brand6	6	6	5	5	5	5	5	6	5	5	5	5	5	1	60
1	Brand4	7	5	4	4	5	5	5	5	4	5	5	4	5	1	60
1	Brand8	8	5	4	5	4	4	5	4	4	4	4	4	4	1	60
1	Brand9	9	5	5	5	5	5	5	5	4	4	4	5	5	1	60
2	Brand1	1	5	6	5	4	5	1	6	5	4	3	2	3	1	56
2	Brand2	2	1	2	1	1	1	1	4	1	1	1	1	1	1	56
2	Brand3	3	4	5	4	3	3	1	4	4	3	3	1	2	1	56
2	Brand4	4	1	6	5	2	6	1	5	3	4	2	1	2	1	56
2	Brand5	5	4	3	4	3	2	1	5	4	3	2	1	2	1	56
2	Brand6	6	4	4	2	1	3	1	3	4	2	1	1	1	1	56
2	Brand4	7	3	4	3	2	3	1	5	4	3	2	1	2	1	56
2	Brand8	8	5	5	5	4	4	1	5	5	3	2	1	2	1	56
2	Brand9	9	3	5	3	1	3	3	5	2	2	4	1	2	1	56
3	Brand1	1	5	6	5	4	5	3	4	5	4	5	3	4	1	41
3	Brand2	2	5	5	6	4	5	5	5	5	5	5	4	5	1	41
3	Brand3	3	5	4	5	4	4	5	6	5	4	4	5	4	1	41
3	Brand4	4	3	3	4	3	4	3	4	4	3	5	3	3	1	41
3	Brand5	5	4	4	5	6	4	4	5	3	5	4	5	3	1	41
3	Brand6	6	3	5	4	4	5	5	5	6	4	4	4	4	1	41
3	Brand4	7	5	4	4	6	4	4	4	5	4	5	5	4	1	41
3	Brand8	8	5	6	5	5	5	5	5	6	4	5	3	5	1	41
3	Brand9	9	4	5	3	5	6	4	5	5	5	4	5	4	1	41
4	Brand1	1	4	4	1	3	3	3	3	3	3	2	3	2	1	61
4	Brand2	2	2	3	3	3	3	3	3	3	2	3	3	3	1	61
4	Brand3	3	4	4	4	4	4	4	4	4	3	3	3	3	1	61
4	Brand4	4	3	3	3	3	3	3	3	3	3	2	2	3	1	61
4	Brand5	5	3	3	3	3	3	3	3	3	3	2	2	3	1	61
4	Brand6	6	3	3	3	3	3	3	3	3	3	3	3	3	1	61
4	Brand4	7	3	3	3	3	3	3	3	3	3	2	3	3	1	61
4	Brand8	8	3	3	3	3	3	3	3	3	2	3	3	3	1	61
4	Brand9	9	2	3	3	3	3	3	3	3	2	3	3	3	1	61
5	Brand1	1	6	6	6	6	5	5	6	5	1	1	1	1	1	48
5	Brand2	2	5	5	4	4	4	4	5	3	1	1	1	1	1	48
5	Brand3	3	5	5	4	5	5	4	4	3	1	1	1	1	1	48
5	Brand4	4	1	4	2	1	3	1	2	1	1	1	1	1	1	48
5	Brand5	5	2	2	2	1	1	1	1	1	1	1	1	1	1	48
5	Brand6	6	2	2	1	1	1	1	2	1	1	1	1	1	1	48
5	Brand4	7	1	2	2	1	2	1	1	1	1	1	1	1	1	48
5	Brand8	8	1	3	2	1	1	1	2	1	1	1	1	1	1	48
5	Brand9	9	2	2	2	2	2	2	2	1	1	1	1	1	1	48
6	Brand1	1	4	3	3	3	3	3	4	3	4	3	2	2	1	38
6	Brand2	2	5	5	5	5	5	4	5	5	5	4	3	4	1	38
6	Brand3	3	4	5	5	5	5	2	3	4	4	2	2	2	1	38
6	Brand4	4	2	2	2	2	2	2	2	2	4	1	1	1	1	38
6	Brand5	5	4	3	3	3	3	3	3	3	5	4	4	4	1	38
6	Brand6	6	2	2	2	2	2	2	2	2	4	2	2	2	1	38

6	Brand4	7	6	6	5	6	6	5	6	6	4	2	2	2	1	38
6	Brand8	8	2	3	3	2	2	2	3	3	4	2	2	2	1	38
6	Brand9	9	2	2	2	3	2	3	2	2	4	2	2	2	1	38
7	Brand1	1	2	4	5	4	4	3	5	3	4	3	2	3	2	48
7	Brand2	2	4	5	5	4	4	3	6	5	3	3	2	3	2	48
7	Brand3	3	4	5	5	4	4	3	4	5	3	3	2	3	2	48
7	Brand4	4	2	4	5	1	4	2	3	3	3	3	2	2	2	48
7	Brand5	5	2	5	3	3	4	3	3	2	4	3	2	2	2	48
7	Brand6	6	3	5	4	3	4	3	6	3	3	3	2	4	2	48
7	Brand4	7	3	5	3	3	4	3	5	3	4	2	2	3	2	48
7	Brand8	8	3	5	3	3	4	3	5	3	3	3	2	3	2	48
7	Brand9	9	3	5	3	3	4	3	5	2	3	3	2	3	2	48
8	Brand1	1	5	6	5	5	5	6	4	4	4	4	4	4	1	51
8	Brand2	2	5	4	4	4	5	4	5	3	3	4	4	4	1	51
8	Brand3	3	4	4	4	4	4	3	4	3	3	4	4	4	1	51
8	Brand4	4	1	2	3	2	3	3	1	1	3	2	3	2	1	51
8	Brand5	5	5	4	4	4	4	4	4	3	4	5	5	3	1	51
8	Brand6	6	5	5	4	4	3	4	5	3	4	4	4	4	1	51
8	Brand4	7	3	3	3	3	4	3	3	2	3	3	3	3	1	51
8	Brand8	8	5	4	4	4	3	3	4	4	4	4	4	4	1	51
8	Brand9	9	3	3	3	4	3	4	4	3	4	4	4	4	1	51
9	Brand1	1	7	6	6	7	6	6	6	7	4	5	5	5	2	59
9	Brand2	2	6	6	6	6	6	6	6	6	5	5	5	5	2	59
9	Brand3	3	5	5	5	5	5	5	5	5	4	5	4	4	2	59
9	Brand4	4	3	3	3	3	3	3	3	2	3	3	3	3	2	59
9	Brand5	5	5	4	4	4	4	4	4	3	4	4	4	4	2	59
9	Brand6	6	5	6	6	6	6	6	6	4	6	6	6	6	2	59
9	Brand4	7	5	5	5	5	5	5	6	5	4	5	4	5	2	59
9	Brand8	8	5	5	4	5	4	5	5	5	4	4	4	4	2	59
9	Brand9	9	7	6	6	7	6	6	6	6	5	5	6	6	2	59
10	Brand1	1	5	4	4	5	6	4	4	5	5	5	3	5	1	57
10	Brand2	2	4	4	3	4	3	5	4	4	4	4	1	1	1	57
10	Brand3	3	4	4	4	5	4	3	4	4	4	4	4	4	1	57
10	Brand4	4	4	4	4	3	3	5	4	4	4	4	3	4	1	57
10	Brand5	5	4	4	3	2	1	5	3	4	4	4	2	1	1	57
10	Brand6	6	4	4	2	2	2	4	4	4	5	5	3	1	1	57
10	Brand4	7	4	4	4	5	4	4	5	4	5	5	3	5	1	57
10	Brand8	8	4	4	2	3	3	5	5	3	4	3	1	4	1	57
10	Brand9	9	4	7	2	3	2	5	5	3	4	3	1	3	1	57
11	Brand1	1	3	3	3	3	3	3	3	3	3	3	3	3	1	51
11	Brand2	2	3	3	3	3	3	3	3	3	3	3	3	3	1	51
11	Brand3	3	3	3	3	3	3	3	3	3	3	3	3	3	1	51
11	Brand4	4	3	3	3	3	3	3	3	3	3	3	3	3	1	51
11	Brand5	5	3	3	3	3	3	3	3	3	3	3	3	3	1	51
11	Brand6	6	3	3	3	3	3	3	3	3	3	3	3	3	1	51
11	Brand4	7	3	3	3	3	3	3	3	3	3	3	3	3	1	51
11	Brand8	8	3	3	3	3	3	3	3	3	3	3	3	3	1	51
11	Brand9	9	3	3	3	3	3	3	3	3	3	3	3	3	1	51
12	Brand1	1	4	4	4	4	4	4	4	4	4	4	4	4	2	61
12	Brand2	2	4	4	4	4	4	4	4	4	4	4	4	4	2	61
12	Brand3	3	4	4	3	4	4	3	4	4	4	4	4	4	2	61
12	Brand4	4	3	4	4	4	4	3	4	3	3	4	4	3	2	61
12	Brand5	5	4	4	4	4	4	3	4	4	4	4	4	4	2	61
12	Brand6	6	4	4	4	4	4	4	4	4	4	4	4	4	2	61

12	Brand4	7	3	4	4	3	4	4	4	3	3	4	4	4	2	61
12	Brand8	8	4	4	4	4	4	4	4	4	3	4	4	4	2	61
12	Brand9	9	4	4	4	4	4	4	4	4	4	4	4	4	2	61
13	Brand1	1	5	5	5	5	5	5	5	5	5	5	5	5	1	52
13	Brand2	2	5	5	5	5	5	5	5	6	6	5	6	5	1	52
13	Brand3	3	5	5	5	5	5	5	5	5	5	5	5	5	1	52
13	Brand4	4	5	5	5	5	5	6	6	6	5	5	5	5	1	52
13	Brand5	5	5	5	5	5	5	5	5	5	5	5	5	5	1	52
13	Brand6	6	5	5	5	5	5	6	5	5	5	5	5	5	1	52
13	Brand4	7	5	5	5	5	5	5	5	5	5	5	5	5	1	52
13	Brand8	8	5	6	6	5	5	5	5	5	5	5	5	5	1	52
13	Brand9	9	5	6	5	5	5	5	5	5	5	5	5	5	1	52
14	Brand1	1	4	4	4	4	4	3	3	3	4	4	5	4	2	43
14	Brand2	2	3	3	3	3	3	3	3	2	4	4	5	4	2	43
14	Brand3	3	3	3	3	3	3	3	3	2	4	4	5	4	2	43
14	Brand4	4	4	4	4	4	4	4	5	3	4	4	5	4	2	43
14	Brand5	5	4	4	4	4	4	4	4	3	4	4	5	4	2	43
14	Brand6	6	4	4	4	4	4	4	4	3	4	4	5	4	2	43
14	Brand4	7	4	4	4	4	4	4	4	3	4	4	5	4	2	43
14	Brand8	8	4	4	4	4	4	4	4	3	4	4	5	4	2	43
14	Brand9	9	4	4	4	4	4	4	4	3	4	4	5	4	2	43
15	Brand1	1	4	4	4	3	3	3	4	3	1	1	1	1	2	42
15	Brand2	2	3	4	3	4	3	2	2	2	1	1	1	1	2	42
15	Brand3	3	1	1	1	1	1	1	1	1	1	1	1	1	2	42
15	Brand4	4	2	2	2	2	4	3	2	3	1	1	1	1	2	42
15	Brand5	5	4	4	4	4	4	3	4	4	1	1	1	1	2	42
15	Brand6	6	5	5	3	5	4	3	6	2	1	1	1	1	2	42
15	Brand4	7	5	5	5	4	4	3	5	3	1	2	1	2	2	42
15	Brand8	8	4	4	4	4	3	4	5	2	1	1	1	1	2	42
15	Brand9	9	3	4	3	5	4	3	5	2	1	2	1	1	2	42
16	Brand1	1	4	4	4	4	4	4	4	4	4	3	5	4	1	39
16	Brand2	2	4	4	4	4	4	4	4	4	4	4	4	4	1	39
16	Brand3	3	4	4	4	4	4	4	4	5	4	4	4	4	1	39
16	Brand4	4	4	4	4	4	4	4	4	4	4	4	4	4	1	39
16	Brand5	5	4	4	4	4	4	4	4	4	4	4	4	4	1	39
16	Brand6	6	5	4	4	5	5	4	4	4	4	4	4	4	1	39
16	Brand4	7	4	4	4	4	4	4	4	4	4	4	4	4	1	39
16	Brand8	8	4	4	4	4	4	4	4	4	4	4	4	4	1	39
16	Brand9	9	4	4	4	4	4	4	4	4	3	4	4	4	1	39
17	Brand1	1	6	7	6	7	7	7	7	4	2	2	2	2	2	61
17	Brand2	2	6	6	6	6	6	6	6	1	1	1	1	1	2	61
17	Brand3	3	5	5	5	4	5	5	5	1	1	1	1	1	2	61
17	Brand4	4	4	4	4	4	4	4	4	1	1	1	1	1	2	61
17	Brand5	5	4	4	4	4	4	4	4	1	1	1	1	1	2	61
17	Brand6	6	4	4	4	4	4	4	4	1	1	1	2	1	2	61
17	Brand4	7	4	4	4	4	4	4	4	1	1	1	1	1	2	61
17	Brand8	8	4	4	4	4	4	4	4	1	1	1	1	1	2	61
17	Brand9	9	4	4	4	4	4	4	4	1	1	1	1	1	2	61
18	Brand1	1	3	2	3	2	2	3	1	2	3	3	3	3	1	65
18	Brand2	2	3	3	3	3	3	4	4	3	3	4	4	4	1	65
18	Brand3	3	4	4	4	3	3	2	2	2	4	3	4	4	1	65
18	Brand4	4	3	3	3	3	3	3	4	3	5	5	4	5	1	65
18	Brand5	5	3	3	3	3	3	3	3	3	3	3	3	3	1	65
18	Brand6	6	6	5	4	5	5	5	6	6	5	5	4	4	1	65

18	Brand4	7	5	5	5	5	5	5	6	6	3	3	3	3	1	65
18	Brand8	8	4	5	4	4	5	5	6	6	3	4	3	4	1	65
18	Brand9	9	5	5	5	4	5	3	4	4	4	4	4	4	1	65
19	Brand1	1	3	3	2	3	3	3	3	4	4	3	3	3	1	57
19	Brand2	2	4	4	3	3	3	2	2	1	5	3	4	3	1	57
19	Brand3	3	3	2	2	2	2	2	2	3	4	4	3	3	1	57
19	Brand4	4	1	1	1	1	1	1	1	2	3	1	1	1	1	57
19	Brand5	5	3	3	2	2	2	2	2	2	4	3	3	3	1	57
19	Brand6	6	3	3	2	3	3	3	3	3	4	3	3	3	1	57
19	Brand4	7	2	2	2	3	2	2	3	3	4	2	2	2	1	57
19	Brand8	8	4	3	4	3	3	3	3	4	3	2	2	3	1	57
19	Brand9	9	3	3	2	2	2	2	2	2	3	2	2	3	1	57
20	Brand1	1	5	5	5	5	4	4	6	5	5	5	5	5	1	54
20	Brand2	2	6	5	6	5	4	4	6	6	5	5	4	5	1	54
20	Brand3	3	5	5	4	4	4	4	3	4	5	5	4	5	1	54
20	Brand4	4	3	5	3	3	3	2	3	3	3	3	2	3	1	54
20	Brand5	5	3	6	5	5	3	3	3	4	5	5	4	5	1	54
20	Brand6	6	5	5	5	5	5	4	4	5	5	5	5	5	1	54
20	Brand4	7	5	5	5	5	5	4	4	4	4	4	4	4	1	54
20	Brand8	8	5	5	5	5	4	4	6	4	2	4	3	4	1	54
20	Brand9	9	5	5	5	5	5	4	5	5	3	4	4	5	1	54
21	Brand1	1	5	5	5	5	6	5	5	5	5	4	5	5	1	61
21	Brand2	2	3	4	5	4	3	3	4	4	3	5	3	4	1	61
21	Brand3	3	4	3	4	4	5	4	4	4	4	3	4	5	1	61
21	Brand4	4	4	3	5	4	4	4	4	3	4	3	4	3	1	61
21	Brand5	5	3	4	4	3	4	3	4	4	4	4	5	4	1	61
21	Brand6	6	3	4	3	3	4	4	3	4	4	3	3	3	1	61
21	Brand4	7	4	4	3	4	3	4	3	4	3	3	4	4	1	61
21	Brand8	8	4	5	4	4	3	5	4	4	4	4	4	4	1	61
21	Brand9	9	3	3	3	2	3	3	3	4	4	4	3	3	1	61
22	Brand1	1	5	4	4	4	5	5	4	4	1	3	5	4	2	47
22	Brand2	2	6	4	4	3	2	3	3	4	3	2	3	3	2	47
22	Brand3	3	1	1	1	1	1	1	1	1	1	1	1	1	2	47
22	Brand4	4	1	1	1	1	1	1	1	1	1	1	1	1	2	47
22	Brand5	5	7	7	7	7	7	7	7	4	7	7	7	7	2	47
22	Brand6	6	7	4	4	7	7	7	7	4	1	1	1	1	2	47
22	Brand4	7	6	6	6	6	6	6	6	6	6	5	6	6	2	47
22	Brand8	8	7	4	4	2	7	7	7	4	1	1	1	1	2	47
22	Brand9	9	6	7	6	6	6	6	6	4	7	7	7	7	2	47
23	Brand1	1	7	7	7	7	7	7	7	7	7	6	6	6	2	35
23	Brand2	2	7	7	7	7	7	7	7	7	7	6	6	6	2	35
23	Brand3	3	4	4	5	5	4	3	2	5	5	5	5	5	2	35
23	Brand4	4	1	1	1	1	1	1	1	1	5	6	5	5	2	35
23	Brand5	5	2	1	1	1	1	1	1	1	5	6	5	6	2	35
23	Brand6	6	1	1	2	1	1	1	2	2	4	4	2	3	2	35
23	Brand4	7	3	2	3	3	2	3	2	3	5	4	4	3	2	35
23	Brand8	8	1	1	1	1	1	1	1	1	5	4	3	3	2	35
23	Brand9	9	1	1	1	1	1	1	1	1	6	6	6	6	2	35
24	Brand1	1	1	3	1	1	1	1	1	1	1	1	1	1	1	54
24	Brand2	2	7	7	7	7	7	7	7	7	7	7	7	7	1	54
24	Brand3	3	1	3	1	1	1	1	1	1	1	1	1	1	1	54
24	Brand4	4	1	1	1	1	1	1	1	1	1	1	1	1	1	54
24	Brand5	5	1	3	1	1	1	1	1	1	1	1	1	1	1	54
24	Brand6	6	1	1	1	1	1	1	3	1	1	1	1	1	1	54

24	Brand4	7	1	1	1	1	1	1	1	1	1	1	1	1	1	54
24	Brand8	8	1	3	1	1	1	1	1	1	1	1	1	1	1	54
24	Brand9	9	1	3	3	1	1	1	1	1	1	1	1	1	1	54
25	Brand1	1	4	3	1	4	4	5	4	3	4	3	5	4	1	48
25	Brand2	2	5	3	2	2	7	7	4	3	4	3	5	4	1	48
25	Brand3	3	5	1	1	1	3	2	3	2	4	2	3	2	1	48
25	Brand4	4	3	1	2	1	2	2	2	2	1	2	4	2	1	48
25	Brand5	5	4	3	4	5	6	4	3	3	6	3	2	1	1	48
25	Brand6	6	4	3	2	5	5	4	3	4	6	2	1	4	1	48
25	Brand4	7	6	3	2	2	6	5	5	3	5	3	4	4	1	48
25	Brand8	8	5	3	4	3	3	4	3	2	5	1	7	4	1	48
25	Brand9	9	5	1	2	4	3	1	5	3	7	5	2	2	1	48
26	Brand1	1	7	7	7	7	7	7	7	7	6	6	5	6	2	57
26	Brand2	2	6	6	5	6	6	6	6	5	5	5	5	5	2	57
26	Brand3	3	6	6	6	6	6	7	7	6	6	5	6	6	2	57
26	Brand4	4	4	4	4	4	4	4	4	4	4	3	4	4	2	57
26	Brand5	5	5	5	5	4	5	5	5	5	5	5	4	4	2	57
26	Brand6	6	7	7	7	7	7	7	7	7	7	6	6	7	2	57
26	Brand4	7	7	6	6	6	6	6	6	5	6	5	5	6	2	57
26	Brand8	8	6	7	7	7	6	6	6	6	6	6	6	7	2	57
26	Brand9	9	7	7	7	7	7	6	7	7	6	6	5	6	2	57
27	Brand1	1	5	5	3	5	5	5	5	5	4	3	3	3	1	40
27	Brand2	2	4	5	5	5	5	3	5	4	3	4	3	4	1	40
27	Brand3	3	2	3	4	2	3	2	3	3	3	4	4	3	1	40
27	Brand4	4	2	3	3	4	3	3	3	3	2	2	3	3	1	40
27	Brand5	5	2	3	3	3	3	4	3	3	3	3	2	3	1	40
27	Brand6	6	4	3	4	3	3	3	3	4	3	3	2	3	1	40
27	Brand4	7	1	5	4	2	3	3	3	3	3	3	2	4	1	40
27	Brand8	8	3	3	4	3	2	3	3	3	2	2	3	3	1	40
27	Brand9	9	3	3	4	2	4	4	4	4	2	2	2	3	1	40
28	Brand1	1	6	6	6	6	5	5	5	4	5	5	5	5	1	66
28	Brand2	2	5	5	4	4	5	4	5	3	4	4	4	4	1	66
28	Brand3	3	4	4	3	3	3	3	3	3	4	5	4	4	1	66
28	Brand4	4	3	3	3	3	3	3	3	2	3	3	3	3	1	66
28	Brand5	5	4	4	4	4	4	4	4	4	3	4	3	4	1	66
28	Brand6	6	5	5	3	4	4	4	4	3	4	4	4	4	1	66
28	Brand4	7	3	3	3	4	3	3	3	3	3	3	3	3	1	66
28	Brand8	8	4	4	4	4	4	4	6	2	3	3	3	3	1	66
28	Brand9	9	3	3	3	3	3	3	3	3	3	3	3	3	1	66
29	Brand1	1	6	5	5	6	5	4	3	2	3	4	2	5	1	38
29	Brand2	2	3	1	3	4	3	6	4	3	1	4	4	2	1	38
29	Brand3	3	6	5	5	2	4	2	1	4	4	5	5	5	1	38
29	Brand4	4	6	6	5	4	3	2	2	5	5	4	2	7	1	38
29	Brand5	5	2	7	6	5	4	3	2	7	5	4	2	4	1	38
29	Brand6	6	6	5	4	3	2	4	3	2	5	3	5	6	1	38
29	Brand4	7	7	6	5	4	3	2	4	3	7	5	4	3	1	38
29	Brand8	8	6	5	5	4	2	3	3	6	6	5	2	5	1	38
29	Brand9	9	5	6	5	4	3	3	4	3	5	3	2	6	1	38
30	Brand1	1	5	5	5	5	6	5	6	5	6	6	3	4	2	46
30	Brand2	2	5	6	6	6	6	5	5	6	5	5	4	5	2	46
30	Brand3	3	5	6	5	4	7	6	6	4	4	4	4	6	2	46
30	Brand4	4	5	5	5	5	6	5	5	5	5	5	3	6	2	46
30	Brand5	5	5	6	6	6	5	4	5	5	5	6	5	5	2	46
30	Brand6	6	5	6	5	5	5	5	5	5	6	5	4	5	2	46

30	Brand4	7	5	5	6	6	5	6	5	4	4	4	3	4	2	46
30	Brand8	8	5	6	4	5	6	6	5	5	5	4	4	5	2	46
30	Brand9	9	5	5	5	5	6	5	5	5	5	5	4	5	2	46
31	Brand1	1	3	3	4	4	4	3	3	3	3	2	2	2	1	55
31	Brand2	2	4	4	4	4	5	3	5	5	3	3	4	3	1	55
31	Brand3	3	4	4	5	5	5	4	4	5	4	5	4	4	1	55
31	Brand4	4	2	2	3	3	3	2	4	2	2	3	3	3	1	55
31	Brand5	5	5	5	4	4	4	5	5	4	3	4	4	3	1	55
31	Brand6	6	2	4	4	4	4	4	4	3	3	4	4	4	1	55
31	Brand4	7	3	5	5	3	2	3	4	3	4	3	3	4	1	55
31	Brand8	8	4	3	4	2	4	3	4	4	4	4	4	4	1	55
31	Brand9	9	3	4	4	4	3	4	4	4	3	4	3	4	1	55
32	Brand1	1	7	7	5	7	7	7	7	4	4	4	4	4	2	50
32	Brand2	2	6	6	5	5	5	5	6	4	4	4	4	4	2	50
32	Brand3	3	5	6	4	5	5	4	4	4	4	4	3	4	2	50
32	Brand4	4	4	4	4	4	4	4	4	3	4	4	3	4	2	50
32	Brand5	5	4	4	4	5	5	4	5	4	4	4	4	4	2	50
32	Brand6	6	6	6	5	6	6	6	7	4	4	4	4	4	2	50
32	Brand4	7	4	4	4	4	4	4	5	4	4	4	4	4	2	50
32	Brand8	8	4	4	4	4	4	4	4	4	4	4	4	4	2	50
32	Brand9	9	4	4	4	4	4	4	4	4	4	4	4	4	2	50
33	Brand1	1	6	6	6	6	5	5	6	6	2	2	2	3	1	59
33	Brand2	2	6	6	6	6	6	5	6	6	2	2	2	3	1	59
33	Brand3	3	6	6	6	6	5	4	6	5	2	2	2	3	1	59
33	Brand4	4	4	5	4	4	4	3	5	4	2	2	2	3	1	59
33	Brand5	5	5	7	5	5	5	4	5	4	2	2	2	3	1	59
33	Brand6	6	7	7	6	6	7	4	7	5	2	2	2	3	1	59
33	Brand4	7	6	6	6	5	5	5	6	5	2	2	2	3	1	59
33	Brand8	8	7	7	5	5	5	4	6	4	2	2	2	3	1	59
33	Brand9	9	6	6	5	6	6	3	6	4	2	2	2	4	1	59
34	Brand1	1	5	5	5	5	5	5	5	5	5	5	5	5	1	55
34	Brand2	2	2	2	3	3	2	2	2	2	2	2	2	2	1	55
34	Brand3	3	5	5	5	5	5	5	5	5	5	5	6	6	1	55
34	Brand4	4	1	1	1	1	1	2	1	1	2	2	2	2	1	55
34	Brand5	5	3	3	3	3	3	3	2	1	2	2	2	3	1	55
34	Brand6	6	3	3	3	3	3	3	3	3	3	3	3	3	1	55
34	Brand4	7	3	3	3	2	3	4	6	6	4	4	4	4	1	55
34	Brand8	8	4	4	4	4	4	5	5	6	3	4	4	4	1	55
34	Brand9	9	3	3	3	3	4	5	4	3	4	3	4	4	1	55
35	Brand1	1	4	4	4	5	3	4	4	3	4	4	4	3	2	55
35	Brand2	2	5	5	4	5	5	4	4	5	4	4	4	5	2	55
35	Brand3	3	1	1	1	1	1	1	1	1	1	1	1	1	2	55
35	Brand4	4	1	1	1	1	1	1	1	1	1	1	1	1	2	55
35	Brand5	5	4	4	4	4	4	3	5	4	4	4	3	4	2	55
35	Brand6	6	4	4	5	4	4	4	4	5	4	4	4	4	2	55
35	Brand4	7	3	4	5	4	4	4	4	4	4	4	4	4	2	55
35	Brand8	8	4	4	4	5	4	4	3	3	4	4	4	4	2	55
35	Brand9	9	3	5	5	4	3	4	4	5	3	4	5	4	2	55
36	Brand1	1	6	6	6	6	6	5	6	4	5	5	5	5	2	41
36	Brand2	2	5	4	4	5	4	4	5	4	4	4	4	4	2	41
36	Brand3	3	5	5	4	5	5	5	5	4	4	4	4	4	2	41
36	Brand4	4	2	3	3	3	3	3	3	3	2	2	2	2	2	41
36	Brand5	5	4	4	4	3	3	4	4	4	3	3	3	3	2	41
36	Brand6	6	4	4	4	3	4	3	4	3	3	3	3	3	2	41

36	Brand4	7	3	3	3	3	3	3	4	3	3	3	3	3	2	41
36	Brand8	8	4	4	4	3	4	3	4	3	3	3	3	3	2	41
36	Brand9	9	3	3	4	3	3	3	4	3	3	3	3	3	2	41
37	Brand1	1	5	5	5	5	5	5	5	5	5	5	4	5	2	55
37	Brand2	2	4	4	4	4	4	4	4	4	4	4	4	4	2	55
37	Brand3	3	4	4	4	4	4	4	4	3	3	3	3	3	2	55
37	Brand4	4	5	4	4	4	4	4	4	4	4	4	4	4	2	55
37	Brand5	5	4	4	4	4	4	4	4	4	4	4	4	4	2	55
37	Brand6	6	5	4	4	4	4	4	4	4	4	4	4	4	2	55
37	Brand4	7	4	4	4	4	4	4	5	4	4	4	4	4	2	55
37	Brand8	8	4	4	4	4	4	4	4	3	4	4	4	4	2	55
37	Brand9	9	5	4	4	4	4	4	4	4	4	4	4	4	2	55
38	Brand1	1	7	6	6	6	6	6	7	6	3	3	3	3	1	56
38	Brand2	2	5	5	6	6	6	6	7	4	3	3	3	4	1	56
38	Brand3	3	5	6	6	6	6	6	6	5	2	4	3	3	1	56
38	Brand4	4	4	4	4	4	4	4	4	4	4	3	3	3	1	56
38	Brand5	5	4	4	4	4	4	4	4	4	3	3	3	3	1	56
38	Brand6	6	6	6	6	6	6	6	7	6	5	5	5	5	1	56
38	Brand4	7	4	4	4	5	4	4	6	5	3	3	3	3	1	56
38	Brand8	8	5	5	5	7	5	5	6	5	3	3	3	4	1	56
38	Brand9	9	5	5	4	4	4	5	5	4	3	3	3	3	1	56
39	Brand1	1	5	5	5	5	5	5	5	5	4	4	4	4	1	67
39	Brand2	2	4	4	4	4	4	4	5	4	1	1	1	1	1	67
39	Brand3	3	4	4	4	4	4	4	4	4	3	3	3	2	1	67
39	Brand4	4	2	2	2	2	2	2	2	2	2	2	1	1	1	67
39	Brand5	5	4	4	4	4	4	4	4	4	1	1	1	1	1	67
39	Brand6	6	5	5	5	5	5	4	5	5	4	4	4	4	1	67
39	Brand4	7	4	4	4	4	4	4	4	4	2	2	1	2	1	67
39	Brand8	8	4	4	4	4	4	4	4	4	3	2	2	2	1	67
39	Brand9	9	4	4	4	4	4	4	4	4	3	3	3	3	1	67
40	Brand1	1	4	4	4	4	4	4	4	4	4	4	4	4	1	69
40	Brand2	2	4	4	4	4	4	4	4	4	4	4	3	4	1	69
40	Brand3	3	4	4	4	4	4	4	4	2	4	4	3	4	1	69
40	Brand4	4	4	4	3	4	3	4	4	3	4	4	4	4	1	69
40	Brand5	5	4	4	4	3	4	4	4	4	4	4	4	3	1	69
40	Brand6	6	4	4	4	4	3	4	4	4	4	4	4	4	1	69
40	Brand4	7	4	4	4	4	4	4	4	4	4	4	4	4	1	69
40	Brand8	8	4	4	4	4	4	4	4	4	4	4	4	4	1	69
40	Brand9	9	4	4	4	4	4	3	4	4	4	4	4	4	1	69
41	Brand1	1	5	6	5	5	5	5	5	5	5	5	5	5	2	34
41	Brand2	2	6	6	6	7	6	5	6	6	4	5	4	5	2	34
41	Brand3	3	6	6	6	5	6	5	6	5	5	4	5	5	2	34
41	Brand4	4	2	2	2	3	3	3	2	2	2	2	3	2	2	34
41	Brand5	5	4	4	3	4	3	4	4	3	3	3	3	3	2	34
41	Brand6	6	3	3	3	3	3	3	2	2	2	3	3	3	2	34
41	Brand4	7	4	4	4	4	4	4	3	3	3	3	3	3	2	34
41	Brand8	8	4	5	5	5	5	5	5	5	4	5	4	4	2	34
41	Brand9	9	5	5	5	5	5	5	4	4	4	4	5	5	2	34
42	Brand1	1	5	6	5	5	4	4	5	4	5	5	5	4	1	56
42	Brand2	2	4	5	4	3	3	2	5	3	5	5	4	4	1	56
42	Brand3	3	4	4	4	4	3	2	4	3	5	4	4	4	1	56
42	Brand4	4	4	5	6	4	3	2	4	3	5	5	5	5	1	56
42	Brand5	5	5	5	5	5	4	5	5	4	4	5	5	5	1	56
42	Brand6	6	6	6	4	5	5	5	6	4	6	6	6	5	1	56

42	Brand4	7	5	6	6	5	5	4	6	5	5	5	5	5	1	56
42	Brand8	8	5	5	4	4	5	4	6	4	5	5	5	5	1	56
42	Brand9	9	7	6	6	7	6	4	7	6	4	5	5	5	1	56
43	Brand1	1	4	4	4	4	4	4	4	4	3	2	2	2	1	47
43	Brand2	2	4	4	4	4	4	4	4	4	3	2	2	2	1	47
43	Brand3	3	3	3	3	3	3	3	2	1	2	2	3	2	1	47
43	Brand4	4	2	2	3	1	1	1	1	1	1	1	1	1	1	47
43	Brand5	5	4	4	4	4	4	4	4	3	3	2	2	2	1	47
43	Brand6	6	4	4	4	4	4	4	4	4	3	2	2	2	1	47
43	Brand4	7	4	4	4	4	4	4	4	4	3	2	2	2	1	47
43	Brand8	8	4	4	4	4	4	4	4	4	3	2	2	2	1	47
43	Brand9	9	4	4	4	4	4	4	4	3	2	2	2	2	1	47
44	Brand1	1	5	5	5	5	5	5	5	5	5	5	5	5	2	34
44	Brand2	2	5	5	5	5	5	5	5	5	5	5	5	5	2	34
44	Brand3	3	5	5	5	5	5	5	5	5	5	5	5	5	2	34
44	Brand4	4	5	5	5	5	5	5	5	5	5	5	5	5	2	34
44	Brand5	5	5	5	5	5	5	5	5	5	5	5	5	5	2	34
44	Brand6	6	5	5	5	5	5	5	5	5	5	5	5	5	2	34
44	Brand4	7	5	5	5	5	5	5	5	5	5	5	5	5	2	34
44	Brand8	8	5	5	5	5	5	5	5	5	5	5	5	5	2	34
44	Brand9	9	5	5	5	5	5	5	5	5	5	5	5	5	2	34
45	Brand1	1	5	6	5	5	5	5	6	5	5	5	5	4	2	57
45	Brand2	2	4	4	4	4	4	4	4	4	4	4	3	4	2	57
45	Brand3	3	4	4	4	4	4	4	4	4	4	4	4	4	2	57
45	Brand4	4	1	1	1	1	4	3	1	1	1	2	2	2	2	57
45	Brand5	5	2	2	2	3	2	2	3	2	2	1	2	2	2	57
45	Brand6	6	4	4	4	4	4	2	3	2	1	4	4	3	2	57
45	Brand4	7	3	3	3	3	3	3	3	3	3	3	2	3	2	57
45	Brand8	8	4	4	4	4	4	4	4	4	4	4	4	3	2	57
45	Brand9	9	2	3	3	3	3	4	4	3	2	2	1	2	2	57
46	Brand1	1	5	6	5	6	7	5	6	4	4	4	4	4	1	47
46	Brand2	2	5	5	4	5	4	4	5	4	4	3	4	4	1	47
46	Brand3	3	2	2	2	1	4	4	2	4	4	4	4	4	1	47
46	Brand4	4	4	4	4	4	4	4	4	4	1	4	4	4	1	47
46	Brand5	5	4	4	4	4	4	4	4	4	4	4	4	4	1	47
46	Brand6	6	3	3	3	2	2	4	3	4	4	4	4	4	1	47
46	Brand4	7	4	4	2	2	2	4	2	4	4	4	4	4	1	47
46	Brand8	8	2	4	4	2	2	4	2	2	4	4	4	4	1	47
46	Brand9	9	4	2	2	2	2	4	4	4	4	4	4	4	1	47
47	Brand1	1	5	4	4	4	4	5	4	4	4	4	4	4	1	61
47	Brand2	2	5	4	4	4	4	4	4	4	4	4	4	4	1	61
47	Brand3	3	5	5	4	4	4	5	4	4	4	4	4	4	1	61
47	Brand4	4	4	4	4	4	4	4	4	4	4	4	4	4	1	61
47	Brand5	5	4	4	4	4	4	4	4	4	4	4	4	4	1	61
47	Brand6	6	4	4	4	4	4	4	4	4	4	4	4	4	1	61
47	Brand4	7	4	4	4	4	4	4	4	4	4	4	4	4	1	61
47	Brand8	8	4	4	4	4	4	4	4	4	4	4	4	4	1	61
47	Brand9	9	4	4	4	4	4	4	4	4	4	4	4	4	1	61
48	Brand1	1	4	4	4	4	4	4	4	4	4	4	4	4	2	36
48	Brand2	2	4	4	4	4	4	4	4	4	4	4	3	4	2	36
48	Brand3	3	4	4	4	4	4	4	4	4	4	4	4	4	2	36
48	Brand4	4	4	4	4	4	4	4	4	4	4	4	4	4	2	36
48	Brand5	5	4	4	4	4	4	4	4	4	4	4	4	4	2	36
48	Brand6	6	4	4	4	4	4	4	4	4	4	4	4	4	2	36

48	Brand4	7	4	4	4	4	4	4	4	4	4	4	4	4	2	36
48	Brand8	8	4	4	4	4	4	4	4	4	4	4	4	4	2	36
48	Brand9	9	4	4	4	4	4	4	4	4	4	4	4	4	2	36
49	Brand1	1	4	4	4	4	4	4	4	4	5	5	5	6	2	43
49	Brand2	2	4	4	4	4	4	4	4	4	4	4	4	4	2	43
49	Brand3	3	5	5	4	5	4	4	5	5	5	5	6	6	2	43
49	Brand4	4	4	5	5	3	3	3	3	3	4	5	5	5	2	43
49	Brand5	5	4	4	4	4	4	4	4	4	4	4	4	4	2	43
49	Brand6	6	4	4	4	4	4	4	4	4	4	6	6	6	2	43
49	Brand4	7	4	5	4	4	4	4	4	4	5	5	5	5	2	43
49	Brand8	8	4	4	4	4	4	4	4	4	4	5	4	5	2	43
49	Brand9	9	4	4	4	4	4	4	4	4	4	4	4	4	2	43
50	Brand1	1	5	5	6	6	6	6	6	7	5	4	4	5	2	50
50	Brand2	2	5	5	5	6	6	5	5	5	5	5	5	5	2	50
50	Brand3	3	4	4	4	4	4	4	4	4	4	4	4	4	2	50
50	Brand4	4	3	3	4	3	3	2	1	2	3	3	3	4	2	50
50	Brand5	5	4	4	4	4	4	4	4	4	3	3	3	3	2	50
50	Brand6	6	5	5	5	6	6	6	6	5	5	5	4	4	2	50
50	Brand4	7	3	4	4	4	3	4	3	3	4	3	4	4	2	50
50	Brand8	8	5	5	5	5	4	5	5	4	5	5	5	5	2	50
50	Brand9	9	4	4	4	4	4	4	4	3	4	4	3	4	2	50
51	Brand1	1	4	4	4	4	4	4	4	4	3	4	4	4	1	67
51	Brand2	2	4	4	4	4	4	4	4	4	3	4	4	4	1	67
51	Brand3	3	4	4	4	4	4	4	4	4	3	4	4	4	1	67
51	Brand4	4	4	4	4	4	4	4	4	4	3	4	4	4	1	67
51	Brand5	5	4	4	4	4	4	4	4	4	3	4	4	4	1	67
51	Brand6	6	4	4	4	4	4	4	4	4	3	4	4	4	1	67
51	Brand4	7	4	4	4	4	4	4	4	4	3	4	4	4	1	67
51	Brand8	8	4	4	4	4	4	4	4	4	3	4	4	4	1	67
51	Brand9	9	4	4	4	4	4	4	4	4	3	4	4	4	1	67
52	Brand1	1	3	3	3	2	2	2	3	3	5	3	4	4	1	39
52	Brand2	2	6	3	4	6	6	4	2	3	3	4	2	1	1	39
52	Brand3	3	5	3	4	5	5	3	3	2	5	2	2	2	1	39
52	Brand4	4	3	6	4	4	4	2	1	1	7	3	5	2	1	39
52	Brand5	5	5	3	2	2	4	7	4	1	4	2	5	2	1	39
52	Brand6	6	5	2	3	4	2	1	2	1	3	3	2	2	1	39
52	Brand4	7	5	2	5	5	1	3	2	4	5	2	2	3	1	39
52	Brand8	8	5	4	6	6	3	4	3	6	4	7	2	4	1	39
52	Brand9	9	5	2	3	4	2	1	3	2	3	3	1	1	1	39
53	Brand1	1	7	7	6	7	6	6	7	6	2	2	2	2	2	57
53	Brand2	2	7	7	6	6	6	6	6	6	2	2	2	2	2	57
53	Brand3	3	5	5	5	5	5	5	4	4	2	2	2	2	2	57
53	Brand4	4	4	4	4	4	4	4	4	4	3	2	2	3	2	57
53	Brand5	5	7	5	5	5	4	4	4	4	2	2	2	2	2	57
53	Brand6	6	7	5	5	5	5	5	6	4	2	2	2	2	2	57
53	Brand4	7	7	7	5	5	5	5	6	4	2	3	2	2	2	57
53	Brand8	8	7	7	5	5	5	6	6	4	2	2	2	2	2	57
53	Brand9	9	7	6	6	6	5	5	5	4	2	2	2	2	2	57
54	Brand1	1	4	4	4	5	4	4	4	5	6	5	6	6	2	48
54	Brand2	2	4	4	3	4	4	4	4	4	4	4	4	4	2	48
54	Brand3	3	3	4	4	4	4	2	4	4	4	4	4	4	2	48
54	Brand4	4	3	3	4	3	3	4	4	5	4	4	5	4	2	48
54	Brand5	5	3	4	3	3	4	3	4	3	4	3	3	3	2	48
54	Brand6	6	4	4	4	3	4	4	4	4	3	3	3	4	2	48

54	Brand4	7	5	5	5	4	4	5	4	4	4	4	5	4	2	48
54	Brand8	8	5	5	5	4	3	4	4	4	4	4	4	4	2	48
54	Brand9	9	4	3	3	3	4	3	5	4	4	3	3	3	2	48
55	Brand1	1	4	4	4	4	4	4	4	4	3	3	3	3	1	48
55	Brand2	2	4	4	4	4	4	4	4	4	3	3	3	3	1	48
55	Brand3	3	4	4	4	4	4	4	4	3	3	3	3	3	1	48
55	Brand4	4	1	1	1	1	1	1	1	1	1	1	1	1	1	48
55	Brand5	5	2	2	2	2	2	2	2	2	2	2	2	2	1	48
55	Brand6	6	2	2	2	2	2	2	2	2	2	2	2	2	1	48
55	Brand4	7	2	2	2	2	2	2	2	2	2	2	2	2	1	48
55	Brand8	8	2	2	2	2	2	2	2	2	2	2	2	2	1	48
55	Brand9	9	2	2	2	2	2	2	2	2	2	1	2	2	1	48
56	Brand1	1	5	5	4	4	5	5	4	4	1	3	2	2	1	66
56	Brand2	2	4	4	4	4	4	3	4	3	3	3	3	3	1	66
56	Brand3	3	3	4	3	4	4	3	4	3	3	3	3	3	1	66
56	Brand4	4	2	2	2	2	2	2	2	2	2	2	2	2	1	66
56	Brand5	5	3	3	3	3	2	3	3	2	3	3	3	3	1	66
56	Brand6	6	3	4	3	4	4	3	4	2	3	3	3	3	1	66
56	Brand4	7	3	3	3	3	3	3	3	3	3	3	3	3	1	66
56	Brand8	8	3	4	3	4	4	4	4	2	3	3	3	3	1	66
56	Brand9	9	3	4	3	3	3	3	4	3	3	3	3	3	1	66
57	Brand1	1	5	6	5	5	4	4	5	4	3	5	5	4	1	43
57	Brand2	2	6	6	4	6	6	6	7	5	5	5	3	2	1	43
57	Brand3	3	2	3	3	3	4	3	2	2	4	2	3	1	1	43
57	Brand4	4	1	1	2	2	2	1	2	1	4	3	1	4	1	43
57	Brand5	5	3	4	4	3	3	2	2	2	4	3	2	2	1	43
57	Brand6	6	4	3	3	4	3	4	4	2	4	3	1	5	1	43
57	Brand4	7	4	4	2	3	4	3	3	3	4	4	2	3	1	43
57	Brand8	8	2	3	2	2	2	3	2	2	3	3	4	2	1	43
57	Brand9	9	4	4	3	1	3	4	4	3	4	1	2	2	1	43
58	Brand1	1	2	2	2	2	2	2	2	2	2	2	2	2	2	53
58	Brand2	2	2	2	2	2	2	2	2	2	2	2	3	2	2	53
58	Brand3	3	1	1	1	1	1	1	1	1	1	1	1	1	2	53
58	Brand4	4	2	2	2	2	2	2	2	2	2	2	1	1	2	53
58	Brand5	5	1	3	2	1	2	1	3	3	1	1	1	1	2	53
58	Brand6	6	2	2	2	2	2	2	2	2	1	2	2	2	2	53
58	Brand4	7	2	2	2	2	2	2	2	2	1	1	1	1	2	53
58	Brand8	8	1	1	1	1	1	1	1	1	1	1	1	1	2	53
58	Brand9	9	1	1	1	1	1	1	1	1	1	1	1	1	2	53
59	Brand1	1	3	4	4	4	4	3	3	3	4	4	4	4	2	37
59	Brand2	2	4	5	5	5	4	3	3	5	5	5	4	4	2	37
59	Brand3	3	4	5	5	4	4	4	4	4	3	4	3	3	2	37
59	Brand4	4	5	4	5	5	4	4	5	4	4	4	4	3	2	37
59	Brand5	5	4	5	5	5	3	3	3	5	5	5	4	3	2	37
59	Brand6	6	5	5	5	4	3	4	4	3	5	4	4	3	2	37
59	Brand4	7	4	5	5	5	3	3	3	5	5	5	4	3	2	37
59	Brand8	8	3	4	5	6	4	4	3	4	5	3	4	4	2	37
59	Brand9	9	4	4	4	4	4	4	4	4	4	4	3	4	2	37
60	Brand1	1	5	6	5	5	6	5	5	5	6	5	5	5	1	55
60	Brand2	2	6	5	6	5	6	5	6	5	5	6	5	5	1	55
60	Brand3	3	5	4	5	5	5	4	4	4	5	5	4	4	1	55
60	Brand4	4	5	5	5	5	4	4	4	5	4	5	4	5	1	55
60	Brand5	5	5	4	4	4	4	4	4	4	3	3	4	3	1	55
60	Brand6	6	4	5	5	5	5	5	4	3	4	3	4	4	1	55

60	Brand4	7	3	4	4	3	4	4	4	4	4	3	3	3	1	55
60	Brand8	8	5	4	5	4	4	4	3	4	4	4	4	4	1	55
60	Brand9	9	4	3	3	3	3	4	3	3	3	3	3	3	1	55
61	Brand1	1	6	7	6	5	7	6	7	7	3	3	3	3	2	42
61	Brand2	2	6	6	6	6	6	6	6	6	2	2	2	3	2	42
61	Brand3	3	1	2	2	2	2	1	1	3	3	3	3	3	2	42
61	Brand4	4	3	3	3	3	3	3	3	3	2	2	3	3	2	42
61	Brand5	5	6	6	6	6	6	6	6	6	6	6	6	6	2	42
61	Brand6	6	5	5	6	6	6	6	6	6	2	2	3	3	2	42
61	Brand4	7	7	6	6	7	7	7	7	5	1	1	1	1	2	42
61	Brand8	8	6	6	6	6	5	6	6	5	2	2	2	3	2	42
61	Brand9	9	6	6	6	6	6	5	5	4	3	3	3	3	2	42
62	Brand1	1	6	6	6	6	6	6	6	6	3	3	3	4	1	42
62	Brand2	2	4	4	4	4	4	5	4	4	4	4	4	4	1	42
62	Brand3	3	2	2	2	2	2	2	2	2	2	3	3	3	1	42
62	Brand4	4	2	2	2	2	2	2	2	2	2	2	2	2	1	42
62	Brand5	5	4	4	4	4	4	4	4	4	3	3	3	3	1	42
62	Brand6	6	5	5	5	5	5	5	5	4	3	3	3	3	1	42
62	Brand4	7	4	4	4	4	4	4	4	3	3	3	3	3	1	42
62	Brand8	8	4	4	4	4	4	4	4	5	3	3	3	3	1	42
62	Brand9	9	4	4	4	5	4	4	4	4	3	3	3	3	1	42
63	Brand1	1	5	5	5	5	5	5	5	5	5	5	5	5	1	61
63	Brand2	2	7	7	7	7	7	7	7	7	5	7	7	7	1	61
63	Brand3	3	4	4	3	4	4	4	4	4	4	4	5	4	1	61
63	Brand4	4	4	4	4	4	4	4	4	4	5	4	4	4	1	61
63	Brand5	5	4	4	4	3	4	4	4	4	4	4	4	3	1	61
63	Brand6	6	4	4	4	4	4	4	4	4	4	4	4	4	1	61
63	Brand4	7	4	4	4	3	4	4	4	4	4	4	4	4	1	61
63	Brand8	8	5	5	5	5	5	5	5	5	5	5	5	5	1	61
63	Brand9	9	4	4	4	5	4	4	4	4	4	4	4	4	1	61
64	Brand1	1	5	5	4	4	5	4	4	4	3	3	4	3	2	29
64	Brand2	2	3	3	4	4	4	4	4	4	3	3	4	4	2	29
64	Brand3	3	4	4	4	4	3	4	3	4	3	3	4	3	2	29
64	Brand4	4	4	4	4	4	4	3	4	4	4	4	4	4	2	29
64	Brand5	5	4	4	4	4	4	4	4	4	3	3	4	3	2	29
64	Brand6	6	4	4	4	4	4	4	4	4	3	3	4	3	2	29
64	Brand4	7	4	4	4	4	4	4	4	4	3	3	4	4	2	29
64	Brand8	8	4	4	4	4	5	4	4	4	3	3	4	3	2	29
64	Brand9	9	4	5	4	4	4	4	4	4	3	3	4	3	2	29
65	Brand1	1	5	5	4	4	5	6	5	4	5	4	3	3	2	46
65	Brand2	2	5	4	4	5	5	4	4	2	4	3	1	4	2	46
65	Brand3	3	5	5	4	4	4	3	5	4	5	4	3	3	2	46
65	Brand4	4	4	5	4	4	3	2	5	5	4	4	4	5	2	46
65	Brand5	5	4	5	4	4	5	4	4	3	5	3	2	4	2	46
65	Brand6	6	5	4	2	1	4	4	3	5	4	3	2	1	2	46
65	Brand4	7	4	5	5	5	5	4	4	2	5	4	3	3	2	46
65	Brand8	8	5	4	4	4	3	4	6	4	5	4	3	4	2	46
65	Brand9	9	4	4	4	5	5	3	4	4	4	3	3	2	2	46
66	Brand1	1	7	7	7	7	7	7	7	7	7	7	7	7	1	51
66	Brand2	2	7	7	7	7	7	7	7	7	7	7	7	7	1	51
66	Brand3	3	1	1	1	1	2	1	1	1	1	1	1	1	1	51
66	Brand4	4	1	1	1	1	1	1	2	1	1	1	1	2	1	51
66	Brand5	5	1	1	1	1	1	1	1	1	1	1	1	1	1	51
66	Brand6	6	1	1	1	1	1	1	1	1	1	1	1	1	1	51

66	Brand4	7	1	1	1	1	1	1	1	1	1	1	1	1	1	51
66	Brand8	8	1	1	1	1	1	1	1	1	1	1	1	1	1	51
66	Brand9	9	1	1	1	1	1	1	1	1	1	1	1	1	1	51
67	Brand1	1	5	5	5	5	5	4	5	4	5	5	4	5	1	60
67	Brand2	2	5	5	5	5	5	4	5	5	5	5	5	5	1	60
67	Brand3	3	3	3	3	3	3	3	2	1	3	3	3	3	1	60
67	Brand4	4	4	4	4	4	4	4	4	1	4	4	4	4	1	60
67	Brand5	5	4	4	4	4	4	4	4	4	4	4	4	4	1	60
67	Brand6	6	4	4	4	4	4	4	4	4	4	4	4	4	1	60
67	Brand4	7	4	4	4	4	4	4	4	4	4	4	4	4	1	60
67	Brand8	8	4	4	4	4	4	4	4	4	4	4	4	4	1	60
67	Brand9	9	4	4	4	4	4	4	4	4	4	4	4	4	1	60
68	Brand1	1	6	6	5	5	6	5	6	5	5	5	5	5	2	51
68	Brand2	2	5	5	5	5	5	5	5	5	4	4	4	4	2	51
68	Brand3	3	4	4	4	4	5	4	5	4	5	5	4	4	2	51
68	Brand4	4	5	4	5	5	5	5	5	5	4	5	5	5	2	51
68	Brand5	5	5	5	5	5	4	5	5	5	4	5	5	5	2	51
68	Brand6	6	5	6	5	6	5	5	5	5	4	4	4	4	2	51
68	Brand4	7	5	5	5	5	4	4	4	5	4	5	3	5	2	51
68	Brand8	8	5	4	4	5	5	5	5	4	5	5	5	4	2	51
68	Brand9	9	4	4	4	5	4	4	5	3	4	4	4	4	2	51
69	Brand1	1	4	4	4	3	4	4	3	3	4	4	3	3	2	33
69	Brand2	2	4	4	5	4	4	4	3	3	5	4	3	3	2	33
69	Brand3	3	4	5	5	4	4	4	5	4	4	4	3	4	2	33
69	Brand4	4	3	3	5	5	4	3	2	4	3	3	3	4	2	33
69	Brand5	5	5	4	5	4	4	5	5	3	4	3	5	4	2	33
69	Brand6	6	4	4	4	3	3	4	4	3	4	3	3	3	2	33
69	Brand4	7	4	4	4	4	3	4	4	3	4	3	3	4	2	33
69	Brand8	8	4	4	4	4	3	4	4	3	4	4	2	3	2	33
69	Brand9	9	5	4	4	3	4	3	4	4	3	3	4	4	2	33
70	Brand1	1	4	4	4	4	4	4	4	4	4	4	4	4	1	48
70	Brand2	2	4	4	4	4	4	4	4	4	4	4	4	4	1	48
70	Brand3	3	4	4	4	4	4	4	4	4	4	4	4	4	1	48
70	Brand4	4	4	4	4	4	4	4	4	4	4	4	4	4	1	48
70	Brand5	5	4	4	4	4	4	4	4	4	4	4	4	4	1	48
70	Brand6	6	4	4	4	4	4	4	4	4	4	4	4	4	1	48
70	Brand4	7	4	4	4	4	4	4	4	4	4	4	4	4	1	48
70	Brand8	8	4	4	4	4	4	4	4	4	4	4	4	4	1	48
70	Brand9	9	4	4	4	4	4	4	4	4	4	4	4	4	1	48
71	Brand1	1	6	6	6	6	6	6	6	6	6	6	6	6	1	64
71	Brand2	2	5	5	5	5	5	5	5	5	5	5	5	5	1	64
71	Brand3	3	5	5	6	5	5	5	5	5	3	4	4	4	1	64
71	Brand4	4	3	3	3	3	3	3	3	3	3	3	3	3	1	64
71	Brand5	5	3	3	3	3	3	3	3	3	3	3	3	3	1	64
71	Brand6	6	4	4	4	4	4	4	4	4	4	4	4	4	1	64
71	Brand4	7	4	4	4	4	4	4	4	4	3	3	3	3	1	64
71	Brand8	8	4	4	4	4	4	4	4	4	4	4	4	4	1	64
71	Brand9	9	4	4	4	4	4	4	4	4	4	4	4	4	1	64
72	Brand1	1	5	5	4	4	4	3	5	3	4	5	3	3	2	40
72	Brand2	2	5	5	4	5	5	4	5	3	4	4	4	3	2	40
72	Brand3	3	4	4	3	3	3	2	2	1	5	4	4	3	2	40
72	Brand4	4	4	3	3	3	2	3	3	1	4	4	4	3	2	40
72	Brand5	5	4	4	4	4	4	3	4	3	5	5	5	5	2	40
72	Brand6	6	7	7	5	7	7	6	6	5	6	6	6	5	2	40

72	Brand4	7	5	5	4	5	4	4	5	3	5	4	4	3	2	40
72	Brand8	8	6	7	4	6	6	6	7	4	5	5	5	5	2	40
72	Brand9	9	7	7	7	7	6	6	7	3	7	6	6	5	2	40
73	Brand1	1	6	6	7	7	7	6	6	6	4	4	5	5	1	64
73	Brand2	2	4	4	4	4	5	5	4	5	4	4	4	4	1	64
73	Brand3	3	1	1	1	1	1	1	1	1	2	2	2	2	1	64
73	Brand4	4	2	3	3	3	3	2	2	2	3	3	3	2	1	64
73	Brand5	5	2	2	3	2	3	2	2	3	3	3	4	3	1	64
73	Brand6	6	4	4	4	4	5	4	5	5	4	5	4	4	1	64
73	Brand4	7	4	4	4	4	4	4	5	4	3	4	4	3	1	64
73	Brand8	8	4	5	5	4	5	4	5	5	4	4	4	4	1	64
73	Brand9	9	5	5	5	4	4	4	5	4	4	5	4	4	1	64
74	Brand1	1	5	6	6	6	6	7	7	7	2	3	3	4	2	30
74	Brand2	2	5	5	4	5	4	5	6	4	3	3	3	2	2	30
74	Brand3	3	4	5	6	6	7	7	6	6	4	4	5	5	2	30
74	Brand4	4	4	4	5	2	4	1	4	4	2	3	1	3	2	30
74	Brand5	5	3	4	4	5	5	6	7	7	3	2	3	3	2	30
74	Brand6	6	5	4	4	5	2	4	3	2	3	1	3	2	2	30
74	Brand4	7	4	3	3	4	4	6	3	4	3	3	3	3	2	30
74	Brand8	8	2	2	3	4	5	3	6	4	2	3	3	3	2	30
74	Brand9	9	3	3	4	1	2	4	3	3	3	1	3	3	2	30
75	Brand1	1	5	5	4	4	4	4	5	4	3	5	4	4	2	33
75	Brand2	2	5	5	5	6	6	5	6	5	3	5	4	4	2	33
75	Brand3	3	5	4	4	5	4	4	5	4	3	4	4	4	2	33
75	Brand4	4	2	2	2	2	2	2	2	2	2	2	2	2	2	33
75	Brand5	5	3	3	3	3	3	3	4	3	3	4	4	4	2	33
75	Brand6	6	4	5	4	3	4	4	5	4	3	4	4	4	2	33
75	Brand4	7	4	5	4	4	4	4	4	3	3	3	3	4	2	33
75	Brand8	8	4	4	4	4	4	4	4	4	3	3	3	3	2	33
75	Brand9	9	4	4	4	4	4	4	4	4	3	4	4	4	2	33
76	Brand1	1	6	6	6	6	6	6	6	6	5	5	3	5	2	40
76	Brand2	2	5	5	5	5	4	5	5	4	4	4	4	4	2	40
76	Brand3	3	5	5	5	5	5	4	5	4	4	4	3	4	2	40
76	Brand4	4	4	4	4	4	4	4	4	4	1	1	1	1	2	40
76	Brand5	5	5	5	5	5	5	5	5	4	4	4	4	4	2	40
76	Brand6	6	4	5	5	4	4	3	5	3	3	3	3	3	2	40
76	Brand4	7	5	5	5	5	4	4	5	4	4	4	4	4	2	40
76	Brand8	8	5	5	5	5	5	4	5	3	4	4	4	4	2	40
76	Brand9	9	5	5	5	5	4	4	5	4	4	4	4	4	2	40
77	Brand1	1	2	1	1	1	1	2	1	1	1	1	1	1	1	34
77	Brand2	2	1	1	1	1	1	1	1	1	1	1	1	1	1	34
77	Brand3	3	1	1	1	1	1	1	1	1	1	1	1	1	1	34
77	Brand4	4	1	1	1	1	1	1	1	1	1	1	1	1	1	34
77	Brand5	5	1	1	1	1	1	1	1	1	1	1	1	1	1	34
77	Brand6	6	1	1	1	1	1	1	1	1	1	1	1	1	1	34
77	Brand4	7	1	1	1	1	1	1	1	1	1	1	1	1	1	34
77	Brand8	8	1	1	1	1	1	1	1	1	1	1	1	1	1	34
77	Brand9	9	1	1	1	1	1	1	1	1	1	1	1	1	1	34
78	Brand1	1	2	3	3	3	3	4	3	2	2	1	2	2	2	69
78	Brand2	2	2	2	2	2	4	3	4	2	2	2	1	3	2	69
78	Brand3	3	3	3	4	3	3	4	4	4	3	2	4	3	2	69
78	Brand4	4	2	3	3	5	5	4	4	4	3	3	4	4	2	69
78	Brand5	5	3	3	4	4	5	3	3	3	3	2	4	3	2	69
78	Brand6	6	5	5	5	5	5	6	4	5	3	4	4	5	2	69

78	Brand4	7	4	4	5	4	3	4	4	4	1	4	2	3	2	69
78	Brand8	8	3	3	4	4	4	3	5	4	3	3	3	4	2	69
78	Brand9	9	2	4	3	3	4	3	3	3	1	2	3	2	2	69
79	Brand1	1	2	2	2	2	3	2	3	3	2	2	2	2	2	58
79	Brand2	2	3	3	3	2	3	3	4	3	1	2	1	2	2	58
79	Brand3	3	2	2	3	2	2	2	3	2	2	2	2	2	2	58
79	Brand4	4	2	1	3	4	3	2	2	2	2	2	1	2	2	58
79	Brand5	5	2	2	1	2	2	2	2	2	2	1	2	2	2	58
79	Brand6	6	4	2	2	3	4	3	3	2	1	2	2	2	2	58
79	Brand4	7	2	2	3	2	3	3	3	2	2	3	1	2	2	58
79	Brand8	8	4	4	2	4	3	2	4	2	1	1	1	1	2	58
79	Brand9	9	3	3	4	3	3	3	3	3	2	2	2	2	2	58
80	Brand1	1	4	5	4	2	3	2	4	4	4	3	3	3	1	42
80	Brand2	2	4	4	4	3	3	2	2	3	3	3	3	3	1	42
80	Brand3	3	5	4	4	3	3	3	2	5	3	3	3	3	1	42
80	Brand4	4	4	4	4	3	3	3	4	4	4	3	3	3	1	42
80	Brand5	5	4	4	4	3	3	3	2	4	4	4	3	3	1	42
80	Brand6	6	3	3	3	1	2	1	3	3	4	4	3	3	1	42
80	Brand4	7	3	3	3	3	3	3	4	3	4	3	2	3	1	42
80	Brand8	8	3	5	4	3	2	1	2	4	4	3	2	2	1	42
80	Brand9	9	5	4	4	4	3	3	5	3	5	4	3	2	1	42
81	Brand1	1	4	4	4	5	4	5	5	4	4	5	3	3	1	45
81	Brand2	2	3	6	3	4	6	4	2	4	4	4	3	4	1	45
81	Brand3	3	2	3	5	6	2	5	6	3	3	5	4	5	1	45
81	Brand4	4	3	5	5	4	4	5	4	4	3	5	6	4	1	45
81	Brand5	5	4	5	4	4	4	5	4	4	4	4	5	4	1	45
81	Brand6	6	5	6	6	3	3	3	4	4	2	4	4	5	1	45
81	Brand4	7	3	5	4	6	4	6	2	3	4	4	6	4	1	45
81	Brand8	8	5	4	4	6	6	4	5	6	4	5	2	4	1	45
81	Brand9	9	5	5	6	4	5	5	4	6	3	3	3	7	1	45
82	Brand1	1	5	6	4	5	5	6	5	4	5	4	4	5	2	51
82	Brand2	2	4	6	4	3	5	4	6	4	3	5	3	4	2	51
82	Brand3	3	5	4	4	3	5	4	5	3	5	4	6	4	2	51
82	Brand4	4	4	6	5	4	5	5	5	5	5	3	5	4	2	51
82	Brand5	5	5	4	5	4	5	5	5	4	5	4	3	4	2	51
82	Brand6	6	5	5	4	6	5	6	4	5	5	4	5	4	2	51
82	Brand4	7	5	4	6	5	4	5	4	4	5	3	3	6	2	51
82	Brand8	8	4	5	5	4	5	5	5	5	5	5	4	5	2	51
82	Brand9	9	5	4	5	5	4	5	5	3	4	6	4	4	2	51
83	Brand1	1	2	3	2	1	3	3	3	2	3	3	4	3	2	44
83	Brand2	2	3	2	3	2	3	4	3	3	3	4	4	4	2	44
83	Brand3	3	3	3	3	2	3	3	3	4	2	3	3	4	2	44
83	Brand4	4	4	3	3	4	4	2	5	4	3	3	4	4	2	44
83	Brand5	5	3	4	3	3	5	5	4	3	1	2	1	1	2	44
83	Brand6	6	4	4	4	3	4	3	3	3	3	3	2	3	2	44
83	Brand4	7	3	3	3	3	3	3	3	3	2	3	3	3	2	44
83	Brand8	8	4	4	3	3	5	5	4	4	3	2	3	4	2	44
83	Brand9	9	3	4	3	4	3	3	4	4	3	2	3	4	2	44
84	Brand1	1	6	7	4	5	4	5	5	5	1	5	5	5	2	38
84	Brand2	2	5	5	5	5	4	5	7	6	1	4	4	4	2	38
84	Brand3	3	5	4	5	5	4	6	5	5	1	3	4	4	2	38
84	Brand4	4	4	4	4	4	4	4	4	4	1	4	4	3	2	38
84	Brand5	5	4	4	4	4	4	4	4	4	1	4	4	4	2	38
84	Brand6	6	4	4	4	4	4	4	4	4	1	1	1	1	2	38

84	Brand4	7	4	4	4	4	4	4	4	4	1	4	4	3	2	38
84	Brand8	8	4	4	4	4	4	4	4	4	1	3	3	3	2	38
84	Brand9	9	4	4	4	4	4	4	4	4	1	3	3	3	2	38
85	Brand1	1	4	5	5	5	5	6	5	5	6	6	6	6	2	34
85	Brand2	2	5	4	5	5	5	6	5	5	5	4	4	5	2	34
85	Brand3	3	5	5	5	4	4	5	4	5	5	5	5	5	2	34
85	Brand4	4	5	5	3	4	5	5	5	3	5	4	4	3	2	34
85	Brand5	5	4	5	4	4	5	4	5	5	5	5	5	5	2	34
85	Brand6	6	5	5	5	5	4	4	5	4	5	5	5	6	2	34
85	Brand4	7	5	4	4	4	4	4	4	4	5	5	5	4	2	34
85	Brand8	8	5	5	5	5	4	4	4	3	5	5	3	4	2	34
85	Brand9	9	4	5	4	4	5	5	4	5	4	4	4	5	2	34
86	Brand1	1	5	5	5	5	5	5	6	5	4	3	3	4	2	52
86	Brand2	2	5	5	5	5	5	5	6	4	4	3	3	3	2	52
86	Brand3	3	3	5	5	5	4	4	5	5	3	3	3	3	2	52
86	Brand4	4	4	4	3	4	4	4	3	3	3	3	3	3	2	52
86	Brand5	5	5	6	6	6	5	5	6	5	4	3	3	4	2	52
86	Brand6	6	6	7	7	7	7	6	7	7	3	5	4	5	2	52
86	Brand4	7	5	5	4	4	4	4	5	5	3	3	4	4	2	52
86	Brand8	8	6	6	6	6	6	6	7	6	3	4	3	4	2	52
86	Brand9	9	7	7	7	7	7	7	7	7	4	4	3	3	2	52
87	Brand1	1	5	5	5	5	5	5	5	5	4	4	4	4	1	58
87	Brand2	2	6	6	6	6	6	6	6	6	4	4	4	4	1	58
87	Brand3	3	5	5	5	5	5	5	5	5	4	4	4	4	1	58
87	Brand4	4	4	4	4	4	4	4	4	4	4	4	4	4	1	58
87	Brand5	5	4	4	4	4	4	4	4	4	4	4	4	4	1	58
87	Brand6	6	4	4	4	4	4	4	4	4	4	4	4	4	1	58
87	Brand4	7	4	4	4	4	4	4	4	4	4	4	4	4	1	58
87	Brand8	8	4	4	4	4	4	4	4	4	4	4	4	4	1	58
87	Brand9	9	4	4	4	4	4	4	4	4	4	4	4	4	1	58
88	Brand1	1	5	4	5	5	5	5	4	4	5	5	5	5	1	41
88	Brand2	2	4	4	4	4	4	4	4	4	5	5	5	5	1	41
88	Brand3	3	3	3	3	3	4	4	3	3	5	5	5	5	1	41
88	Brand4	4	4	4	4	4	4	4	4	4	5	5	5	5	1	41
88	Brand5	5	4	4	5	3	4	4	3	3	5	4	5	5	1	41
88	Brand6	6	4	5	5	4	4	4	4	4	5	5	5	5	1	41
88	Brand4	7	4	4	4	4	4	4	4	4	5	5	5	5	1	41
88	Brand8	8	4	4	4	4	4	5	4	4	5	5	5	5	1	41
88	Brand9	9	4	5	4	4	4	4	4	4	5	5	5	5	1	41
89	Brand1	1	4	4	4	4	4	5	4	4	2	2	2	2	1	45
89	Brand2	2	4	5	4	4	5	4	4	4	2	2	2	3	1	45
89	Brand3	3	4	4	4	4	4	4	4	4	2	3	2	1	1	45
89	Brand4	4	2	2	2	2	2	2	2	2	2	2	2	2	1	45
89	Brand5	5	4	4	4	4	4	4	4	4	1	3	3	2	1	45
89	Brand6	6	4	4	4	4	4	4	4	4	2	2	2	1	1	45
89	Brand4	7	4	5	4	4	4	4	4	5	2	2	2	2	1	45
89	Brand8	8	4	4	4	4	4	4	5	4	2	2	1	1	1	45
89	Brand9	9	4	4	4	4	4	4	4	4	2	2	2	2	1	45
90	Brand1	1	1	1	1	1	1	1	1	1	1	1	1	1	1	64
90	Brand2	2	7	7	7	7	7	5	7	7	5	3	4	3	1	64
90	Brand3	3	1	1	1	1	1	1	1	1	3	2	2	2	1	64
90	Brand4	4	1	1	1	1	1	1	1	1	1	1	1	1	1	64
90	Brand5	5	1	1	1	1	1	1	1	1	1	1	1	1	1	64
90	Brand6	6	1	1	1	1	1	1	1	1	1	1	1	1	1	64

90	Brand4	7	1	1	1	1	1	1	1	1	1	1	1	1	1	64
90	Brand8	8	1	1	1	1	1	1	1	1	1	1	1	1	1	64
90	Brand9	9	7	7	7	7	7	7	7	7	3	3	3	3	1	64
91	Brand1	1	6	7	6	7	6	5	6	7	4	5	5	6	2	37
91	Brand2	2	4	6	5	5	5	6	6	5	4	5	5	5	2	37
91	Brand3	3	4	5	4	4	4	4	4	4	4	4	4	5	2	37
91	Brand4	4	2	3	5	4	4	3	3	2	4	3	3	2	2	37
91	Brand5	5	4	4	4	4	4	4	4	3	4	4	3	3	2	37
91	Brand6	6	4	4	4	3	5	4	4	3	4	3	3	2	2	37
91	Brand4	7	4	4	4	4	4	4	4	3	4	3	3	3	2	37
91	Brand8	8	4	4	4	4	4	4	3	2	4	3	3	3	2	37
91	Brand9	9	4	5	4	4	4	4	4	2	4	3	3	3	2	37
92	Brand1	1	5	4	4	4	5	5	5	4	4	4	4	4	2	27
92	Brand2	2	4	4	4	4	4	4	4	4	3	3	3	3	2	27
92	Brand3	3	4	4	4	4	4	4	4	4	4	4	4	4	2	27
92	Brand4	4	2	3	3	2	3	2	3	2	3	3	3	3	2	27
92	Brand5	5	3	4	3	3	4	3	3	3	3	3	3	3	2	27
92	Brand6	6	3	3	3	3	3	3	3	3	3	3	3	3	2	27
92	Brand4	7	3	3	3	3	3	3	3	3	3	3	3	3	2	27
92	Brand8	8	3	4	3	3	4	3	3	3	3	3	3	3	2	27
92	Brand9	9	3	4	3	3	3	3	3	3	3	3	3	3	2	27
93	Brand1	1	5	6	4	5	4	5	4	5	1	4	4	4	1	36
93	Brand2	2	4	4	4	4	4	4	4	4	4	3	4	4	1	36
93	Brand3	3	2	4	4	2	4	3	1	1	1	3	4	3	1	36
93	Brand4	4	1	3	3	2	4	4	4	1	1	3	3	3	1	36
93	Brand5	5	1	4	1	1	4	4	4	1	1	4	4	4	1	36
93	Brand6	6	4	5	4	3	4	4	4	4	2	5	4	4	1	36
93	Brand4	7	4	4	4	1	4	4	2	1	4	4	4	4	1	36
93	Brand8	8	4	5	4	3	4	4	4	1	1	4	4	4	1	36
93	Brand9	9	2	3	4	2	4	4	3	1	1	3	3	4	1	36
94	Brand1	1	4	4	5	5	6	4	5	5	4	4	3	4	1	49
94	Brand2	2	5	6	6	5	6	4	6	5	5	3	4	2	1	49
94	Brand3	3	4	4	4	4	4	3	5	5	3	2	1	2	1	49
94	Brand4	4	2	2	2	2	1	2	2	1	3	4	3	3	1	49
94	Brand5	5	4	4	4	4	5	3	3	6	4	4	3	3	1	49
94	Brand6	6	5	4	4	4	4	4	3	2	5	3	3	3	1	49
94	Brand4	7	4	5	4	5	4	6	4	4	2	5	5	3	1	49
94	Brand8	8	5	5	4	4	4	3	2	1	4	3	2	2	1	49
94	Brand9	9	4	4	4	4	4	3	2	4	4	4	4	3	1	49
95	Brand1	1	5	5	4	5	5	5	5	4	4	4	4	4	1	49
95	Brand2	2	5	5	4	5	4	5	6	5	4	4	4	4	1	49
95	Brand3	3	5	5	4	4	4	4	4	4	3	3	3	3	1	49
95	Brand4	4	2	2	2	2	2	2	2	2	2	2	2	2	1	49
95	Brand5	5	3	3	3	3	3	3	3	3	3	3	3	3	1	49
95	Brand6	6	5	6	5	5	5	5	6	4	4	4	4	4	1	49
95	Brand4	7	4	4	4	4	4	4	6	4	3	3	3	3	1	49
95	Brand8	8	4	5	4	5	4	4	6	4	3	3	3	3	1	49
95	Brand9	9	5	5	5	6	5	4	6	4	4	3	3	3	1	49
96	Brand1	1	7	6	6	6	6	6	5	6	3	5	5	5	1	45
96	Brand2	2	5	6	5	5	5	5	6	5	3	4	4	4	1	45
96	Brand3	3	3	3	2	3	2	2	2	1	3	3	3	3	1	45
96	Brand4	4	5	6	4	5	5	4	5	3	3	5	4	4	1	45
96	Brand5	5	4	4	4	4	4	4	4	4	5	5	5	5	1	45
96	Brand6	6	7	7	7	7	7	7	7	7	2	5	6	6	1	45

96	Brand4	7	7	7	7	7	7	7	7	7	5	6	6	6	1	45
96	Brand8	8	4	4	4	4	4	4	4	4	4	4	4	4	1	45
96	Brand9	9	6	6	7	7	6	6	6	5	6	6	6	6	1	45
97	Brand1	1	6	6	6	6	6	6	6	7	6	6	6	6	2	46
97	Brand2	2	5	5	6	6	5	5	6	5	5	4	4	4	2	46
97	Brand3	3	4	5	5	5	4	4	4	4	5	5	4	5	2	46
97	Brand4	4	4	4	4	4	4	4	4	3	4	4	4	4	2	46
97	Brand5	5	5	5	5	6	6	4	5	4	5	5	5	5	2	46
97	Brand6	6	6	6	6	6	6	6	6	6	6	6	6	6	2	46
97	Brand4	7	5	5	4	5	5	5	5	5	4	5	5	5	2	46
97	Brand8	8	6	6	6	6	5	6	6	6	6	6	5	6	2	46
97	Brand9	9	5	5	5	5	5	5	5	5	4	5	5	5	2	46
98	Brand1	1	6	5	5	6	5	5	5	5	6	6	5	5	2	43
98	Brand2	2	5	6	5	5	5	5	6	6	6	5	5	5	2	43
98	Brand3	3	6	6	6	6	6	6	6	6	6	6	5	5	2	43
98	Brand4	4	5	6	7	5	5	5	6	6	6	6	6	5	2	43
98	Brand5	5	6	6	6	6	5	6	6	6	5	5	4	5	2	43
98	Brand6	6	6	5	5	6	6	5	5	5	6	5	5	6	2	43
98	Brand4	7	6	5	6	6	6	6	6	6	6	5	6	5	2	43
98	Brand8	8	6	6	6	5	6	6	6	5	5	5	6	5	2	43
98	Brand9	9	6	6	7	5	7	6	6	5	6	6	5	6	2	43
99	Brand1	1	4	4	4	4	4	4	4	4	4	4	4	4	2	27
99	Brand2	2	5	4	4	4	3	5	4	4	4	4	4	4	2	27
99	Brand3	3	4	4	4	4	4	4	4	4	2	1	1	1	2	27
99	Brand4	4	4	4	4	4	4	4	4	4	4	4	4	4	2	27
99	Brand5	5	4	4	4	4	4	4	4	4	1	1	1	1	2	27
99	Brand6	6	4	4	4	4	4	4	4	5	1	1	1	1	2	27
99	Brand4	7	4	4	4	4	4	4	4	4	1	1	1	1	2	27
99	Brand8	8	4	4	4	4	4	4	4	4	1	1	1	1	2	27
99	Brand9	9	4	4	4	4	4	3	4	4	1	1	1	1	2	27
100	Brand1	1	6	5	5	5	6	6	6	7	3	2	2	3	2	31
100	Brand2	2	5	6	5	5	4	4	4	4	2	1	1	1	2	31
100	Brand3	3	3	4	5	5	4	4	4	4	2	1	1	1	2	31
100	Brand4	4	2	3	2	2	2	1	3	1	1	1	1	1	2	31
100	Brand5	5	4	4	4	4	3	5	5	5	1	1	1	1	2	31
100	Brand6	6	5	5	5	5	5	3	4	4	2	1	1	1	2	31
100	Brand4	7	4	4	5	5	5	5	5	5	1	1	1	1	2	31
100	Brand8	8	5	3	4	3	2	4	4	4	1	1	1	1	2	31
100	Brand9	9	5	4	5	5	4	5	6	6	1	1	1	1	2	31
101	Brand1	1	5	5	4	5	4	4	5	5	5	5	4	4	1	51
101	Brand2	2	3	4	3	3	3	3	4	3	4	4	5	5	1	51
101	Brand3	3	4	4	5	4	4	3	3	4	4	4	5	5	1	51
101	Brand4	4	4	4	5	4	4	4	4	4	4	5	5	4	1	51
101	Brand5	5	4	4	4	4	4	4	5	4	4	4	5	4	1	51
101	Brand6	6	4	4	4	4	4	4	4	3	5	5	5	4	1	51
101	Brand4	7	4	4	3	3	3	3	4	4	4	4	5	4	1	51
101	Brand8	8	4	5	4	4	4	4	5	4	4	5	5	4	1	51
101	Brand9	9	4	4	4	4	4	4	4	3	4	4	5	4	1	51
102	Brand1	1	5	5	5	4	5	6	6	6	5	5	5	4	2	46
102	Brand2	2	4	4	4	4	4	4	4	4	4	4	4	4	2	46
102	Brand3	3	4	5	4	5	4	3	4	5	4	4	4	4	2	46
102	Brand4	4	3	4	4	4	4	3	4	4	3	4	4	4	2	46
102	Brand5	5	5	4	5	5	4	5	5	5	4	4	4	3	2	46
102	Brand6	6	4	4	4	5	4	4	5	4	4	4	5	4	2	46

102	Brand4	7	5	5	4	4	4	4	5	4	4	4	3	4	2	46
102	Brand8	8	4	5	4	4	4	5	4	4	4	4	4	4	2	46
102	Brand9	9	4	4	4	4	4	4	4	3	4	4	4	3	2	46
103	Brand1	1	5	5	5	5	5	3	4	4	3	2	2	2	1	32
103	Brand2	2	5	4	4	3	4	4	4	4	2	2	2	2	1	32
103	Brand3	3	4	4	4	3	3	2	3	4	2	3	2	2	1	32
103	Brand4	4	3	3	3	2	3	2	3	3	3	2	3	2	1	32
103	Brand5	5	3	3	3	3	3	2	3	3	3	2	2	3	1	32
103	Brand6	6	4	4	6	4	4	4	4	4	3	2	3	2	1	32
103	Brand4	7	5	4	3	3	4	3	3	5	3	3	3	3	1	32
103	Brand8	8	4	4	6	3	4	4	4	3	3	3	2	2	1	32
103	Brand9	9	4	5	4	3	3	3	4	4	3	3	2	3	1	32
104	Brand1	1	1	1	1	1	1	1	1	1	1	1	1	1	1	52
104	Brand2	2	1	1	1	1	1	1	1	1	1	1	1	1	1	52
104	Brand3	3	1	1	1	1	1	1	1	1	1	1	1	1	1	52
104	Brand4	4	1	1	1	1	1	1	1	1	1	1	1	1	1	52
104	Brand5	5	1	1	1	1	1	1	1	1	1	1	1	1	1	52
104	Brand6	6	1	1	1	1	1	1	1	1	1	1	1	1	1	52
104	Brand4	7	1	1	1	1	1	1	1	1	1	1	1	1	1	52
104	Brand8	8	1	1	1	1	1	1	1	1	1	1	1	1	1	52
104	Brand9	9	1	1	1	1	1	1	1	1	1	1	1	1	1	52
105	Brand1	1	4	4	4	4	4	4	4	4	4	4	4	4	2	39
105	Brand2	2	4	4	4	4	4	4	4	4	4	4	4	4	2	39
105	Brand3	3	6	6	6	4	4	4	4	4	4	4	4	3	2	39
105	Brand4	4	2	2	2	2	2	2	2	2	4	4	4	4	2	39
105	Brand5	5	4	4	4	4	4	4	4	4	4	4	4	4	2	39
105	Brand6	6	4	4	4	4	4	4	4	4	4	4	4	4	2	39
105	Brand4	7	4	4	4	4	4	4	4	4	4	4	4	4	2	39
105	Brand8	8	5	5	5	5	5	5	5	5	4	4	4	4	2	39
105	Brand9	9	3	3	3	3	3	3	3	3	4	4	4	4	2	39
106	Brand1	1	6	6	5	7	7	7	7	7	4	5	3	4	2	28
106	Brand2	2	6	6	6	7	7	6	7	7	4	4	4	2	2	28
106	Brand3	3	5	5	5	6	6	6	6	6	3	4	4	4	2	28
106	Brand4	4	4	6	5	4	4	4	5	6	2	2	2	1	2	28
106	Brand5	5	4	4	4	4	4	4	5	5	3	2	2	1	2	28
106	Brand6	6	5	6	5	5	5	5	7	7	2	2	2	1	2	28
106	Brand4	7	5	6	5	5	5	4	6	7	2	2	2	1	2	28
106	Brand8	8	7	7	6	7	6	6	7	7	3	3	2	1	2	28
106	Brand9	9	5	4	3	4	3	3	4	5	3	2	2	1	2	28
107	Brand1	1	5	5	6	6	6	6	6	5	6	5	6	6	2	40
107	Brand2	2	5	6	5	6	6	5	5	5	5	5	5	5	2	40
107	Brand3	3	6	4	5	5	6	5	4	5	6	5	5	5	2	40
107	Brand4	4	5	5	5	5	5	5	5	5	5	5	5	5	2	40
107	Brand5	5	5	5	5	6	6	5	5	5	5	6	6	5	2	40
107	Brand6	6	5	6	5	5	6	5	5	6	6	5	5	6	2	40
107	Brand4	7	5	5	5	5	5	5	5	5	5	5	5	5	2	40
107	Brand8	8	5	6	6	6	5	5	6	6	5	5	5	5	2	40
107	Brand9	9	6	6	5	6	6	5	5	5	5	5	6	6	2	40
108	Brand1	1	6	6	4	4	5	5	6	5	6	6	6	5	1	51
108	Brand2	2	6	6	6	7	5	4	7	7	6	6	5	6	1	51
108	Brand3	3	5	6	4	4	3	2	4	5	6	6	6	5	1	51
108	Brand4	4	5	5	5	4	5	4	6	5	4	5	4	4	1	51
108	Brand5	5	6	6	5	6	6	5	5	5	5	5	5	5	1	51
108	Brand6	6	6	7	5	5	5	5	6	4	5	5	4	5	1	51

108	Brand4	7	6	7	6	6	6	5	6	5	5	6	6	6	1	51
108	Brand8	8	7	7	6	7	6	6	7	6	5	5	5	6	1	51
108	Brand9	9	5	5	4	5	4	4	5	5	5	5	6	5	1	51
109	Brand1	1	7	7	4	6	6	2	6	4	4	4	2	2	1	60
109	Brand2	2	7	7	6	7	6	2	7	3	4	3	2	4	1	60
109	Brand3	3	6	6	4	6	6	2	6	4	4	4	2	2	1	60
109	Brand4	4	2	2	2	2	2	2	2	2	2	2	2	2	1	60
109	Brand5	5	3	4	3	3	2	2	3	3	4	4	2	2	1	60
109	Brand6	6	6	7	4	6	6	2	6	4	4	4	2	2	1	60
109	Brand4	7	4	5	4	4	4	2	4	4	4	4	2	2	1	60
109	Brand8	8	4	4	4	4	4	2	4	4	4	4	2	2	1	60
109	Brand9	9	4	5	4	5	4	2	4	4	4	4	2	2	1	60
110	Brand1	1	7	7	5	7	7	6	7	7	4	4	4	4	2	33
110	Brand2	2	7	7	6	6	7	6	7	6	4	4	4	4	2	33
110	Brand3	3	6	6	7	7	7	5	6	6	4	4	4	4	2	33
110	Brand4	4	5	5	4	4	4	5	4	2	4	4	4	3	2	33
110	Brand5	5	6	6	5	5	5	5	6	2	4	4	4	4	2	33
110	Brand6	6	7	7	4	4	4	4	4	1	3	3	4	4	2	33
110	Brand4	7	7	7	5	4	4	4	5	2	4	4	4	4	2	33
110	Brand8	8	5	5	6	6	6	6	6	6	4	4	4	4	2	33
110	Brand9	9	3	3	3	3	3	3	4	1	4	4	4	4	2	33
111	Brand1	1	6	6	6	7	6	6	6	6	4	4	4	5	2	41
111	Brand2	2	2	2	2	2	2	2	2	3	2	2	2	2	2	41
111	Brand3	3	3	2	2	2	2	2	2	2	2	2	2	2	2	41
111	Brand4	4	3	2	2	2	2	2	2	2	2	2	3	2	2	41
111	Brand5	5	4	3	4	4	4	4	4	4	3	4	4	4	2	41
111	Brand6	6	3	3	3	3	4	3	4	3	3	3	3	3	2	41
111	Brand4	7	4	4	4	4	4	4	4	4	4	4	4	4	2	41
111	Brand8	8	3	3	3	3	3	3	3	3	3	3	3	3	2	41
111	Brand9	9	4	4	3	3	3	3	3	3	3	3	3	3	2	41
112	Brand1	1	4	5	5	4	3	3	4	3	3	5	5	4	2	23
112	Brand2	2	3	3	3	3	4	4	5	3	3	4	3	4	2	23
112	Brand3	3	1	1	1	2	2	1	1	1	3	2	3	3	2	23
112	Brand4	4	5	4	3	3	2	1	2	2	2	3	2	4	2	23
112	Brand5	5	3	4	3	4	3	2	3	4	3	2	3	4	2	23
112	Brand6	6	4	4	5	4	5	4	5	4	4	5	4	4	2	23
112	Brand4	7	3	2	2	3	3	2	3	2	2	3	2	3	2	23
112	Brand8	8	2	3	3	3	3	4	3	2	2	3	3	3	2	23
112	Brand9	9	4	4	3	4	4	4	4	4	3	4	4	4	2	23
113	Brand1	1	5	5	4	7	7	4	7	4	5	5	4	4	1	52
113	Brand2	2	5	7	4	5	4	4	7	4	4	4	5	5	1	52
113	Brand3	3	3	7	4	5	7	3	7	3	6	3	4	3	1	52
113	Brand4	4	3	3	3	3	3	3	5	1	3	3	3	3	1	52
113	Brand5	5	4	4	4	3	4	1	7	4	1	4	3	3	1	52
113	Brand6	6	3	3	3	3	3	1	7	3	3	3	3	2	1	52
113	Brand4	7	5	5	5	4	5	4	7	4	4	4	4	4	1	52
113	Brand8	8	3	3	3	2	2	1	7	3	4	4	4	3	1	52
113	Brand9	9	5	5	5	7	4	4	7	4	4	4	4	4	1	52
114	Brand1	1	5	6	6	6	6	6	6	6	4	5	4	4	2	37
114	Brand2	2	5	7	6	6	6	7	6	6	5	5	5	5	2	37
114	Brand3	3	3	4	3	4	4	4	4	4	4	4	3	3	2	37
114	Brand4	4	3	4	4	3	3	4	4	4	4	4	4	4	2	37
114	Brand5	5	5	6	5	6	5	6	6	6	5	5	5	5	2	37
114	Brand6	6	6	6	7	5	6	6	6	6	5	5	5	6	2	37

114	Brand4	7	5	5	6	5	6	6	6	6	6	6	5	6	2	37
114	Brand8	8	5	6	5	6	6	6	6	4	4	4	4	5	2	37
114	Brand9	9	5	5	5	5	5	6	5	5	5	6	6	6	2	37
115	Brand1	1	7	7	7	7	7	5	7	6	1	3	3	3	1	50
115	Brand2	2	7	7	7	7	7	7	7	7	3	3	3	3	1	50
115	Brand3	3	1	4	1	1	1	1	1	1	3	3	3	3	1	50
115	Brand4	4	4	4	4	4	4	4	4	4	3	3	3	3	1	50
115	Brand5	5	5	5	7	5	4	4	6	5	3	3	3	3	1	50
115	Brand6	6	5	6	5	7	5	4	7	5	3	3	3	3	1	50
115	Brand4	7	5	6	5	5	4	4	7	4	3	3	3	3	1	50
115	Brand8	8	5	6	6	6	5	4	7	5	3	3	3	3	1	50
115	Brand9	9	7	7	7	7	5	4	5	5	3	3	3	3	1	50
116	Brand1	1	5	5	4	4	5	4	4	4	3	4	3	3	2	46
116	Brand2	2	4	4	4	4	4	3	4	4	3	3	3	3	2	46
116	Brand3	3	2	3	3	3	3	3	1	2	1	2	2	3	2	46
116	Brand4	4	3	4	3	3	4	3	3	3	1	3	3	3	2	46
116	Brand5	5	4	4	3	3	3	4	2	2	1	2	3	3	2	46
116	Brand6	6	3	4	3	3	3	3	1	2	1	3	3	3	2	46
116	Brand4	7	4	4	4	4	4	4	4	4	2	3	3	3	2	46
116	Brand8	8	4	4	3	3	3	4	2	3	1	3	3	3	2	46
116	Brand9	9	4	5	4	3	3	4	3	3	1	3	3	3	2	46
117	Brand1	1	6	6	6	6	5	7	6	5	4	5	5	5	2	30
117	Brand2	2	6	6	7	6	6	6	7	6	5	6	6	6	2	30
117	Brand3	3	3	3	3	3	3	3	3	4	3	3	3	3	2	30
117	Brand4	4	5	4	3	4	5	4	4	4	3	3	3	3	2	30
117	Brand5	5	3	4	4	3	4	4	4	4	2	2	3	3	2	30
117	Brand6	6	4	4	4	4	4	5	4	3	4	4	3	4	2	30
117	Brand4	7	4	4	4	4	4	4	4	4	3	3	3	4	2	30
117	Brand8	8	3	4	4	4	4	4	4	4	3	4	4	4	2	30
117	Brand9	9	3	4	4	4	5	4	4	4	4	3	4	3	2	30
118	Brand1	1	3	4	3	4	4	3	4	4	3	3	3	4	1	62
118	Brand2	2	3	3	3	3	3	4	4	3	4	4	3	4	1	62
118	Brand3	3	4	4	5	5	4	4	4	4	4	4	4	4	1	62
118	Brand4	4	3	3	3	3	3	3	3	3	3	2	3	3	1	62
118	Brand5	5	3	3	3	4	5	4	4	4	3	3	3	3	1	62
118	Brand6	6	2	2	2	2	2	2	4	3	4	4	3	4	1	62
118	Brand4	7	3	3	3	3	3	3	3	3	2	2	2	2	1	62
118	Brand8	8	3	3	3	3	3	3	3	3	3	3	3	3	1	62
118	Brand9	9	4	4	4	4	4	4	4	3	2	2	2	2	1	62
119	Brand1	1	4	4	4	4	4	4	4	4	3	2	3	3	2	46
119	Brand2	2	4	4	4	4	4	4	4	4	4	3	2	2	2	46
119	Brand3	3	4	4	4	4	4	4	4	4	2	2	2	2	2	46
119	Brand4	4	3	3	3	4	3	3	3	3	2	2	2	2	2	46
119	Brand5	5	5	4	4	4	4	4	4	4	2	2	2	2	2	46
119	Brand6	6	4	4	4	4	4	4	4	4	3	3	3	4	2	46
119	Brand4	7	4	4	4	4	4	4	4	4	3	3	2	3	2	46
119	Brand8	8	4	3	3	4	5	4	4	4	2	2	2	2	2	46
119	Brand9	9	4	4	4	4	4	4	4	4	4	3	2	2	2	46
120	Brand1	1	4	4	4	5	4	4	4	4	3	4	4	4	1	53
120	Brand2	2	3	3	3	3	3	3	3	3	2	3	3	3	1	53
120	Brand3	3	3	3	3	3	4	2	3	3	3	3	3	3	1	53
120	Brand4	4	3	3	2	2	1	2	3	3	3	3	3	3	1	53
120	Brand5	5	2	2	2	2	2	2	1	2	2	2	2	2	1	53
120	Brand6	6	4	4	4	4	4	4	5	4	5	5	5	5	1	53

120	Brand4	7	3	3	3	3	3	3	3	3	3	3	3	3	1	53
120	Brand8	8	4	4	4	4	4	5	5	5	4	3	3	3	1	53
120	Brand9	9	4	4	4	4	4	4	3	3	4	4	4	4	1	53
121	Brand1	1	4	5	5	5	5	5	5	4	4	4	4	4	2	38
121	Brand2	2	5	5	5	5	5	5	5	4	4	4	4	4	2	38
121	Brand3	3	5	4	5	5	4	4	4	4	4	4	4	4	2	38
121	Brand4	4	4	4	4	4	4	4	1	1	4	4	4	4	2	38
121	Brand5	5	4	4	4	4	4	4	4	1	4	4	4	4	2	38
121	Brand6	6	4	4	4	4	4	4	3	1	4	4	4	4	2	38
121	Brand4	7	4	4	4	4	4	4	4	1	4	4	4	4	2	38
121	Brand8	8	4	4	4	4	4	4	4	1	4	4	4	4	2	38
121	Brand9	9	4	4	4	4	4	4	4	1	4	4	4	4	2	38
122	Brand1	1	7	7	7	7	6	5	7	5	3	3	3	3	1	49
122	Brand2	2	5	4	4	4	4	3	4	5	3	3	3	3	1	49
122	Brand3	3	3	3	3	3	3	3	3	3	3	3	3	3	1	49
122	Brand4	4	2	2	2	2	2	2	2	2	2	2	2	2	1	49
122	Brand5	5	3	3	3	3	3	3	3	3	3	2	2	2	1	49
122	Brand6	6	5	5	5	5	5	5	5	4	3	3	3	3	1	49
122	Brand4	7	4	3	3	3	3	3	3	3	5	3	3	2	1	49
122	Brand8	8	4	4	4	3	4	4	4	4	3	3	3	3	1	49
122	Brand9	9	5	5	5	5	5	5	4	5	3	3	3	3	1	49
123	Brand1	1	5	5	4	5	4	4	4	4	5	5	5	5	2	42
123	Brand2	2	5	5	6	5	6	4	5	5	5	4	5	5	2	42
123	Brand3	3	4	4	5	4	4	4	5	4	4	5	5	5	2	42
123	Brand4	4	3	3	5	5	5	4	5	5	4	4	4	3	2	42
123	Brand5	5	4	3	4	4	4	4	4	4	5	4	5	5	2	42
123	Brand6	6	4	3	3	4	4	4	4	4	5	3	4	4	2	42
123	Brand4	7	5	4	4	4	4	4	5	5	4	5	5	5	2	42
123	Brand8	8	4	4	5	5	4	4	5	4	5	5	5	4	2	42
123	Brand9	9	4	4	5	5	6	5	4	4	5	4	4	4	2	42
124	Brand1	1	5	5	5	5	4	5	4	5	5	5	5	5	2	50
124	Brand2	2	4	5	4	4	4	4	4	4	5	4	4	4	2	50
124	Brand3	3	3	4	4	3	3	3	3	3	4	4	4	5	2	50
124	Brand4	4	3	3	3	3	4	3	3	3	4	4	4	4	2	50
124	Brand5	5	3	3	4	3	3	3	4	4	4	4	3	3	2	50
124	Brand6	6	6	5	5	4	5	4	6	4	4	4	3	4	2	50
124	Brand4	7	5	5	4	4	4	4	5	4	4	4	4	4	2	50
124	Brand8	8	6	4	5	4	5	4	6	4	4	4	4	4	2	50
124	Brand9	9	4	4	3	4	3	3	3	3	4	4	4	4	2	50
125	Brand1	1	5	5	5	5	5	5	5	4	3	3	3	3	2	67
125	Brand2	2	4	4	4	4	4	4	4	4	3	3	3	3	2	67
125	Brand3	3	5	5	4	4	4	4	4	4	3	3	3	3	2	67
125	Brand4	4	3	3	5	3	3	3	3	3	3	2	3	3	2	67
125	Brand5	5	4	4	4	4	4	4	4	4	3	3	3	3	2	67
125	Brand6	6	5	5	5	5	5	5	5	4	3	3	3	3	2	67
125	Brand4	7	4	4	4	4	4	4	4	3	3	3	3	3	2	67
125	Brand8	8	4	4	4	4	4	4	4	4	3	3	3	3	2	67
125	Brand9	9	4	4	3	4	4	4	4	3	3	3	3	3	2	67
126	Brand1	1	5	4	5	5	5	5	5	5	5	4	4	4	2	39
126	Brand2	2	5	5	5	5	4	5	4	4	5	5	5	5	2	39
126	Brand3	3	5	5	4	4	4	4	4	4	5	5	5	5	2	39
126	Brand4	4	3	4	4	3	4	4	4	1	4	4	4	4	2	39
126	Brand5	5	4	4	4	3	4	4	4	4	4	4	4	4	2	39
126	Brand6	6	4	4	4	4	4	4	3	3	4	4	4	4	2	39

126	Brand4	7	4	4	4	3	4	4	4	2	4	4	4	4	2	39
126	Brand8	8	3	3	2	3	4	4	4	2	4	4	4	4	2	39
126	Brand9	9	2	3	3	4	4	4	2	3	3	3	3	3	2	39
127	Brand1	1	6	6	6	6	6	5	6	4	4	4	4	4	2	33
127	Brand2	2	6	6	6	6	6	6	6	5	6	5	4	4	2	33
127	Brand3	3	5	5	5	5	5	4	4	4	4	4	4	4	2	33
127	Brand4	4	5	5	5	5	5	4	5	4	5	4	4	4	2	33
127	Brand5	5	6	6	6	6	6	5	6	5	4	4	4	4	2	33
127	Brand6	6	6	6	6	6	6	6	6	4	5	4	4	4	2	33
127	Brand4	7	6	6	6	6	6	5	6	5	5	5	4	4	2	33
127	Brand8	8	6	6	6	6	6	5	6	5	5	4	4	4	2	33
127	Brand9	9	5	5	5	5	5	5	5	5	4	4	4	4	2	33
128	Brand1	1	7	7	7	7	7	7	7	7	5	6	6	6	1	47
128	Brand2	2	6	6	7	7	7	7	7	7	4	4	6	6	1	47
128	Brand3	3	7	7	7	7	7	7	7	7	7	7	7	7	1	47
128	Brand4	4	5	5	5	4	4	4	4	3	3	3	4	3	1	47
128	Brand5	5	3	4	4	5	5	4	4	4	4	4	2	4	1	47
128	Brand6	6	6	6	6	6	7	7	7	7	7	7	5	5	1	47
128	Brand4	7	4	5	4	5	4	4	4	4	3	3	3	4	1	47
128	Brand8	8	5	4	4	4	4	4	4	4	2	2	2	4	1	47
128	Brand9	9	4	5	5	6	6	5	5	6	4	5	5	4	1	47
129	Brand1	1	5	4	4	4	5	5	6	5	5	4	3	4	2	22
129	Brand2	2	5	5	4	4	4	4	5	4	4	2	4	2	2	22
129	Brand3	3	4	4	3	3	4	4	3	4	4	2	3	4	2	22
129	Brand4	4	4	3	2	4	4	4	3	2	4	3	2	1	2	22
129	Brand5	5	3	3	4	4	4	5	4	4	3	2	3	4	2	22
129	Brand6	6	4	3	3	5	4	4	5	4	4	3	1	1	2	22
129	Brand4	7	4	4	3	3	4	4	4	4	4	3	1	1	2	22
129	Brand8	8	4	4	4	5	6	6	5	4	5	4	3	4	2	22
129	Brand9	9	4	4	3	2	4	4	4	3	5	3	1	2	2	22
130	Brand1	1	7	7	7	7	6	5	7	7	1	4	3	4	1	50
130	Brand2	2	5	5	5	5	4	4	6	5	1	3	3	3	1	50
130	Brand3	3	1	1	2	1	1	1	1	1	1	2	2	2	1	50
130	Brand4	4	3	3	3	3	3	3	3	3	1	2	2	2	1	50
130	Brand5	5	5	5	5	5	5	4	7	5	1	3	3	3	1	50
130	Brand6	6	5	6	6	5	5	5	7	5	1	3	3	3	1	50
130	Brand4	7	4	4	4	4	3	4	4	4	1	3	3	3	1	50
130	Brand8	8	7	7	7	7	7	6	7	7	1	5	5	5	1	50
130	Brand9	9	7	7	5	6	6	5	7	5	1	4	3	3	1	50
131	Brand1	1	7	7	7	7	7	7	7	7	7	7	7	7	1	45
131	Brand2	2	4	4	4	4	4	4	5	5	4	4	5	5	1	45
131	Brand3	3	1	1	1	1	1	1	1	1	1	1	1	1	1	45
131	Brand4	4	5	5	6	5	4	5	4	4	1	1	1	1	1	45
131	Brand5	5	3	4	4	4	4	4	4	4	3	5	4	4	1	45
131	Brand6	6	5	4	4	4	4	4	4	5	4	4	3	4	1	45
131	Brand4	7	4	4	4	4	4	4	4	4	4	5	4	4	1	45
131	Brand8	8	4	4	4	5	4	4	4	4	4	5	4	4	1	45
131	Brand9	9	4	4	3	4	4	4	4	4	4	4	3	4	1	45
132	Brand1	1	5	5	5	5	5	4	5	5	4	4	4	4	1	44
132	Brand2	2	4	4	4	4	4	4	4	4	4	4	4	4	1	44
132	Brand3	3	4	4	4	4	4	4	4	4	4	4	4	4	1	44
132	Brand4	4	4	4	4	4	4	4	4	4	4	4	4	4	1	44
132	Brand5	5	4	4	4	4	4	4	4	4	4	4	4	4	1	44
132	Brand6	6	4	4	4	4	4	4	4	4	4	4	4	4	1	44

132	Brand4	7	4	4	4	4	4	4	4	4	4	4	4	4	1	44
132	Brand8	8	4	4	4	4	4	4	4	4	4	4	4	4	1	44
132	Brand9	9	4	4	4	4	4	4	4	4	4	4	4	4	1	44
133	Brand1	1	2	4	4	4	3	5	4	3	4	5	3	3	1	29
133	Brand2	2	3	4	3	5	5	5	5	5	5	4	3	5	1	29
133	Brand3	3	4	5	5	5	4	5	5	4	4	4	4	4	1	29
133	Brand4	4	5	6	4	4	3	5	4	3	3	5	3	5	1	29
133	Brand5	5	4	6	5	5	4	4	4	4	4	3	2	4	1	29
133	Brand6	6	5	5	4	4	3	5	5	3	4	4	2	5	1	29
133	Brand4	7	3	5	4	6	5	4	4	4	4	4	2	6	1	29
133	Brand8	8	4	5	4	5	5	3	4	5	3	4	3	4	1	29
133	Brand9	9	3	5	4	4	3	3	4	4	5	4	3	3	1	29
134	Brand1	1	5	5	5	6	6	7	6	6	5	6	5	5	2	28
134	Brand2	2	6	5	4	4	5	5	3	2	5	5	4	5	2	28
134	Brand3	3	2	5	4	2	2	1	1	2	5	5	4	5	2	28
134	Brand4	4	3	5	4	2	3	2	3	1	2	4	3	3	2	28
134	Brand5	5	2	3	2	1	4	2	2	1	3	4	3	3	2	28
134	Brand6	6	4	5	3	3	4	6	5	2	5	6	5	5	2	28
134	Brand4	7	5	5	5	6	6	6	6	3	5	6	5	5	2	28
134	Brand8	8	3	4	2	3	3	2	1	1	5	5	4	4	2	28
134	Brand9	9	2	3	2	1	4	4	2	1	2	3	2	2	2	28
135	Brand1	1	3	3	3	3	3	4	3	3	4	4	4	4	2	26
135	Brand2	2	4	4	4	4	4	4	4	4	4	4	4	4	2	26
135	Brand3	3	2	2	2	3	3	3	3	2	4	2	3	3	2	26
135	Brand4	4	3	3	3	3	3	3	3	3	3	3	3	3	2	26
135	Brand5	5	3	3	3	3	3	3	3	3	3	3	3	3	2	26
135	Brand6	6	4	4	4	4	3	3	3	3	3	3	3	3	2	26
135	Brand4	7	3	3	3	3	3	3	3	3	3	3	3	4	2	26
135	Brand8	8	3	3	3	3	3	3	3	3	3	3	3	3	2	26
135	Brand9	9	4	4	3	3	3	4	4	4	3	3	3	2	2	26
136	Brand1	1	6	7	7	6	6	5	6	5	5	6	6	6	1	32
136	Brand2	2	7	7	5	7	6	6	6	6	5	5	5	6	1	32
136	Brand3	3	5	5	4	5	5	5	5	5	6	5	5	6	1	32
136	Brand4	4	2	3	3	3	3	2	2	2	2	2	2	3	1	32
136	Brand5	5	5	5	6	6	5	5	4	4	4	5	5	5	1	32
136	Brand6	6	5	5	5	6	6	5	5	5	5	4	4	6	1	32
136	Brand4	7	3	3	3	5	3	4	4	4	5	5	4	6	1	32
136	Brand8	8	4	3	4	4	6	2	5	6	4	4	3	4	1	32
136	Brand9	9	4	4	3	3	4	4	4	4	4	5	4	5	1	32
137	Brand1	1	2	3	4	3	4	4	4	3	2	4	3	3	2	23
137	Brand2	2	2	4	3	3	3	3	3	4	3	4	2	3	2	23
137	Brand3	3	4	3	3	3	4	3	4	2	3	3	2	3	2	23
137	Brand4	4	3	3	2	3	5	3	3	2	3	3	3	3	2	23
137	Brand5	5	2	3	3	3	3	3	3	3	3	3	3	3	2	23
137	Brand6	6	3	4	3	3	3	4	4	3	3	4	1	2	2	23
137	Brand4	7	3	4	4	4	3	3	3	3	3	4	2	3	2	23
137	Brand8	8	3	3	2	3	3	4	4	4	3	2	1	2	2	23
137	Brand9	9	3	2	2	3	3	3	3	3	3	3	2	2	2	23
138	Brand1	1	6	6	6	6	7	5	6	6	5	5	4	5	1	45
138	Brand2	2	6	5	6	5	5	4	6	5	5	5	4	5	1	45
138	Brand3	3	1	4	4	1	2	1	1	3	3	3	3	4	1	45
138	Brand4	4	1	1	1	1	1	1	1	1	1	1	1	1	1	45
138	Brand5	5	2	3	2	2	3	3	4	3	3	3	2	3	1	45
138	Brand6	6	3	4	1	1	1	4	1	1	4	4	4	4	1	45

138	Brand4	7	1	3	2	4	3	3	4	3	4	3	4	4	1	45
138	Brand8	8	3	4	3	3	4	4	4	4	3	4	3	3	1	45
138	Brand9	9	2	2	3	3	3	4	3	4	3	4	2	3	1	45
139	Brand1	1	5	6	4	5	4	5	5	5	3	3	3	3	2	26
139	Brand2	2	5	4	5	4	4	4	4	5	4	3	3	3	2	26
139	Brand3	3	5	5	5	5	5	4	4	4	5	3	3	3	2	26
139	Brand4	4	4	4	4	4	3	3	3	3	3	3	3	3	2	26
139	Brand5	5	3	3	3	3	4	5	4	4	3	3	3	3	2	26
139	Brand6	6	5	5	5	5	5	5	5	4	3	3	3	3	2	26
139	Brand4	7	3	3	3	4	3	3	4	4	3	3	3	3	2	26
139	Brand8	8	3	3	4	4	3	3	4	3	4	4	3	3	2	26
139	Brand9	9	4	4	4	4	5	5	4	5	3	3	3	3	2	26
140	Brand1	1	6	6	5	6	5	5	6	4	1	3	3	2	1	44
140	Brand2	2	6	6	5	5	6	5	5	5	1	5	4	5	1	44
140	Brand3	3	5	5	6	6	5	5	4	4	1	4	4	2	1	44
140	Brand4	4	2	4	2	2	2	2	2	2	1	1	1	1	1	44
140	Brand5	5	4	5	5	4	4	4	5	4	1	3	3	3	1	44
140	Brand6	6	5	5	4	4	5	4	3	3	1	3	2	2	1	44
140	Brand4	7	3	5	3	3	4	3	3	3	1	2	2	1	1	44
140	Brand8	8	4	3	4	4	4	3	3	3	1	2	3	2	1	44
140	Brand9	9	3	4	4	3	3	4	3	3	1	2	2	2	1	44
141	Brand1	1	5	5	5	5	5	4	5	5	5	4	4	4	2	42
141	Brand2	2	5	5	5	5	5	5	5	5	5	4	4	4	2	42
141	Brand3	3	3	3	3	3	3	3	1	3	5	4	4	4	2	42
141	Brand4	4	3	2	3	3	3	3	4	2	4	4	4	4	2	42
141	Brand5	5	4	4	4	4	3	4	5	4	5	4	4	4	2	42
141	Brand6	6	3	4	4	3	3	4	4	4	5	4	4	4	2	42
141	Brand4	7	4	5	5	4	3	4	4	4	5	5	4	4	2	42
141	Brand8	8	3	3	4	4	2	3	3	3	5	4	4	4	2	42
141	Brand9	9	4	5	5	4	4	4	4	3	5	4	4	4	2	42
142	Brand1	1	6	7	7	7	7	7	7	7	7	7	7	7	1	44
142	Brand2	2	5	5	5	5	5	6	7	7	7	7	7	7	1	44
142	Brand3	3	4	4	4	5	4	4	4	4	3	3	2	3	1	44
142	Brand4	4	2	3	3	3	3	3	3	3	3	3	3	3	1	44
142	Brand5	5	3	4	3	4	3	3	4	3	2	3	2	3	1	44
142	Brand6	6	5	5	5	5	5	4	4	3	4	4	3	4	1	44
142	Brand4	7	4	5	4	4	4	4	4	4	4	4	5	4	1	44
142	Brand8	8	5	3	4	5	4	5	5	5	4	3	4	5	1	44
142	Brand9	9	3	5	5	5	4	4	4	4	3	5	4	5	1	44
143	Brand1	1	6	6	6	6	6	6	6	6	3	3	3	3	1	69
143	Brand2	2	7	6	6	7	4	5	7	6	3	4	3	3	1	69
143	Brand3	3	2	2	3	3	2	2	2	1	2	2	1	1	1	69
143	Brand4	4	2	3	2	2	2	1	2	1	2	2	2	2	1	69
143	Brand5	5	2	1	1	1	1	1	1	1	2	1	1	1	1	69
143	Brand6	6	5	6	6	5	5	6	5	4	3	2	2	2	1	69
143	Brand4	7	4	4	4	4	4	4	3	3	3	3	3	3	1	69
143	Brand8	8	4	4	4	4	4	4	4	4	2	3	2	2	1	69
143	Brand9	9	4	4	4	4	3	6	6	5	3	3	2	2	1	69
144	Brand1	1	5	4	4	5	5	3	4	5	4	4	4	4	2	32
144	Brand2	2	4	3	4	4	4	3	3	3	4	3	3	3	2	32
144	Brand3	3	4	4	4	3	3	3	4	3	4	4	4	4	2	32
144	Brand4	4	1	1	1	1	1	1	1	1	2	2	2	2	2	32
144	Brand5	5	3	3	3	3	3	3	3	3	2	2	2	2	2	32
144	Brand6	6	3	3	3	3	3	3	3	2	3	3	3	3	2	32

144	Brand4	7	2	2	2	2	2	2	2	2	2	2	2	2	2	32
144	Brand8	8	3	3	3	2	4	3	3	1	2	2	3	3	2	32
144	Brand9	9	2	3	2	2	2	2	2	2	2	2	2	2	2	32
145	Brand1	1	3	3	4	3	3	4	4	3	1	3	3	2	2	55
145	Brand2	2	4	4	4	4	4	4	4	4	3	3	2	3	2	55
145	Brand3	3	5	5	4	5	5	4	5	4	3	3	4	3	2	55
145	Brand4	4	2	2	3	3	3	3	3	2	3	3	2	2	2	55
145	Brand5	5	3	2	3	2	3	3	3	3	2	2	3	2	2	55
145	Brand6	6	3	3	3	4	4	3	4	3	1	2	2	2	2	55
145	Brand4	7	2	2	2	3	3	3	3	3	3	3	2	3	2	55
145	Brand8	8	3	3	2	3	3	2	4	3	3	4	3	4	2	55
145	Brand9	9	2	2	3	3	3	3	3	2	2	2	3	3	2	55
146	Brand1	1	7	7	7	7	7	6	7	6	4	5	5	6	2	36
146	Brand2	2	7	7	7	7	7	7	7	7	4	4	2	3	2	36
146	Brand3	3	4	4	4	3	5	5	4	4	4	3	3	4	2	36
146	Brand4	4	7	7	7	7	7	7	7	7	2	5	3	3	2	36
146	Brand5	5	7	6	6	6	6	6	6	6	3	3	4	4	2	36
146	Brand6	6	7	7	7	7	7	7	7	7	4	3	3	4	2	36
146	Brand4	7	3	2	4	4	4	4	5	7	3	4	4	5	2	36
146	Brand8	8	4	3	3	3	6	5	5	6	4	5	6	6	2	36
146	Brand9	9	3	3	3	4	3	3	3	3	3	3	4	4	2	36
147	Brand1	1	5	5	5	6	6	6	6	5	5	5	5	6	1	53
147	Brand2	2	4	4	4	4	4	4	4	3	4	4	4	4	1	53
147	Brand3	3	3	3	2	2	2	2	2	2	3	3	3	3	1	53
147	Brand4	4	2	2	2	2	2	2	2	2	2	2	2	2	1	53
147	Brand5	5	3	3	3	3	3	3	3	3	3	3	3	3	1	53
147	Brand6	6	4	4	4	4	4	4	4	3	4	4	6	4	1	53
147	Brand4	7	4	4	3	3	3	3	4	3	3	3	3	3	1	53
147	Brand8	8	4	4	4	4	4	4	4	3	3	3	3	3	1	53
147	Brand9	9	4	4	4	4	4	4	5	4	4	4	5	5	1	53
148	Brand1	1	6	6	6	7	6	6	6	6	1	2	2	2	1	68
148	Brand2	2	4	4	4	4	4	4	4	4	2	3	3	3	1	68
148	Brand3	3	2	2	2	2	3	2	1	1	1	2	2	2	1	68
148	Brand4	4	3	3	3	3	3	3	3	2	1	3	3	3	1	68
148	Brand5	5	3	3	3	3	3	3	3	1	1	2	2	2	1	68
148	Brand6	6	3	3	3	3	3	3	3	1	1	2	2	2	1	68
148	Brand4	7	3	3	3	3	3	3	3	1	2	2	2	2	1	68
148	Brand8	8	2	2	2	2	2	2	2	1	1	2	2	2	1	68
148	Brand9	9	3	4	3	4	3	3	3	1	1	1	3	2	1	68
149	Brand1	1	6	7	7	7	6	7	6	6	5	6	6	6	1	30
149	Brand2	2	6	6	6	7	5	6	7	6	5	6	6	6	1	30
149	Brand3	3	4	4	3	4	4	4	3	4	3	4	4	3	1	30
149	Brand4	4	4	4	3	3	3	3	3	2	3	3	3	2	1	30
149	Brand5	5	4	5	4	4	4	4	4	4	3	4	4	4	1	30
149	Brand6	6	6	5	5	6	6	5	6	5	4	6	6	5	1	30
149	Brand4	7	5	4	4	4	3	5	4	4	3	3	4	3	1	30
149	Brand8	8	5	5	5	6	6	5	6	6	4	5	5	4	1	30
149	Brand9	9	4	5	4	4	4	5	5	4	5	5	6	5	1	30
150	Brand1	1	4	4	4	5	4	4	3	3	4	5	4	4	1	40
150	Brand2	2	4	4	4	4	4	4	5	5	4	4	4	4	1	40
150	Brand3	3	4	4	4	3	3	4	3	4	4	4	5	4	1	40
150	Brand4	4	3	3	2	3	2	3	3	2	4	3	3	3	1	40
150	Brand5	5	3	4	4	4	4	4	3	3	4	4	3	4	1	40
150	Brand6	6	4	4	3	4	4	4	4	3	4	4	4	4	1	40

150	Brand4	7	4	4	3	4	4	4	4	3	3	4	4	4	1	40
150	Brand8	8	4	4	4	4	4	4	4	3	4	4	4	4	1	40
150	Brand9	9	3	3	3	3	3	4	4	3	4	4	3	4	1	40
151	Brand1	1	4	4	4	4	4	4	4	4	4	4	3	4	1	39
151	Brand2	2	3	3	3	3	4	4	4	4	3	4	3	3	1	39
151	Brand3	3	3	4	4	4	4	3	3	3	3	3	3	3	1	39
151	Brand4	4	3	4	3	5	3	3	3	3	3	3	4	3	1	39
151	Brand5	5	3	4	4	3	3	3	3	4	3	4	3	4	1	39
151	Brand6	6	3	3	3	3	3	3	3	3	3	3	3	3	1	39
151	Brand4	7	4	4	4	4	4	4	4	3	4	4	4	4	1	39
151	Brand8	8	4	4	3	3	4	4	5	4	4	4	4	3	1	39
151	Brand9	9	3	4	4	4	5	4	4	4	4	4	4	3	1	39
152	Brand1	1	7	7	7	7	6	6	7	7	7	7	7	7	2	19
152	Brand2	2	7	7	7	7	7	6	7	7	7	7	7	7	2	19
152	Brand3	3	5	6	7	6	7	6	5	6	7	7	5	6	2	19
152	Brand4	4	7	7	6	7	6	6	7	7	7	6	7	7	2	19
152	Brand5	5	5	5	6	7	7	6	7	6	6	7	6	6	2	19
152	Brand6	6	6	7	6	7	6	7	5	7	6	7	6	7	2	19
152	Brand4	7	6	7	6	7	6	7	7	6	6	7	6	6	2	19
152	Brand8	8	6	7	6	7	6	7	5	7	6	7	5	7	2	19
152	Brand9	9	6	7	6	7	6	6	5	7	7	6	7	6	2	19
153	Brand1	1	6	6	4	5	5	4	5	4	4	4	4	4	1	21
153	Brand2	2	5	4	4	5	5	6	4	4	4	4	4	4	1	21
153	Brand3	3	2	4	4	4	4	1	2	3	4	2	4	4	1	21
153	Brand4	4	4	4	4	4	4	5	4	4	3	4	4	4	1	21
153	Brand5	5	4	4	4	4	4	4	4	4	4	4	4	4	1	21
153	Brand6	6	4	4	4	4	4	4	4	4	4	4	4	4	1	21
153	Brand4	7	4	4	4	4	4	4	5	2	3	5	4	4	1	21
153	Brand8	8	4	5	4	4	4	4	5	4	4	4	4	4	1	21
153	Brand9	9	4	5	4	4	5	4	4	4	4	4	4	4	1	21
154	Brand1	1	6	6	6	6	6	6	6	6	4	6	6	6	2	53
154	Brand2	2	6	6	6	6	6	6	6	6	4	5	4	6	2	53
154	Brand3	3	5	5	5	5	5	5	5	4	4	4	4	4	2	53
154	Brand4	4	4	4	4	4	4	4	4	4	4	4	4	4	2	53
154	Brand5	5	4	4	4	4	4	4	4	4	4	4	4	4	2	53
154	Brand6	6	5	5	5	5	5	5	5	5	4	4	4	4	2	53
154	Brand4	7	4	4	4	4	4	4	5	4	4	4	4	4	2	53
154	Brand8	8	4	4	4	4	4	4	4	4	4	4	4	4	2	53
154	Brand9	9	6	6	6	6	6	6	6	6	5	5	5	5	2	53
155	Brand1	1	6	6	6	6	6	6	6	6	6	6	6	6	1	43
155	Brand2	2	5	5	5	5	5	4	5	5	5	5	5	5	1	43
155	Brand3	3	4	4	4	4	4	4	4	4	4	4	4	4	1	43
155	Brand4	4	3	3	3	3	3	3	3	3	3	3	3	3	1	43
155	Brand5	5	3	3	3	3	3	3	3	3	3	3	3	3	1	43
155	Brand6	6	4	4	4	4	4	4	4	4	4	4	4	4	1	43
155	Brand4	7	3	3	3	3	3	3	3	3	3	3	3	3	1	43
155	Brand8	8	4	4	4	3	3	3	3	3	3	3	3	3	1	43
155	Brand9	9	3	3	3	3	3	3	3	3	3	3	3	3	1	43
156	Brand1	1	3	2	3	2	1	1	1	1	3	2	1	1	2	45
156	Brand2	2	5	6	4	5	4	3	5	5	5	6	6	6	2	45
156	Brand3	3	3	2	1	1	1	1	1	1	2	2	1	1	2	45
156	Brand4	4	4	3	3	4	3	2	2	2	3	2	2	2	2	45
156	Brand5	5	5	4	4	5	3	3	3	4	4	4	5	4	2	45
156	Brand6	6	6	6	4	6	7	4	4	6	5	5	4	4	2	45

156	Brand4	7	5	5	6	6	5	5	6	6	4	5	5	5	2	45
156	Brand8	8	6	6	5	6	6	5	6	6	5	5	6	5	2	45
156	Brand9	9	7	6	4	5	4	4	6	4	4	6	6	5	2	45
157	Brand1	1	4	4	3	4	5	4	4	4	4	4	2	2	2	48
157	Brand2	2	4	4	4	3	2	1	4	2	2	3	2	1	2	48
157	Brand3	3	4	4	4	3	2	1	7	6	3	3	2	1	2	48
157	Brand4	4	3	3	3	3	2	1	5	3	2	3	2	4	2	48
157	Brand5	5	4	4	3	3	2	1	5	5	3	3	2	1	2	48
157	Brand6	6	4	5	4	3	2	1	7	7	3	3	2	1	2	48
157	Brand4	7	2	3	3	3	2	1	4	3	3	3	2	1	2	48
157	Brand8	8	3	3	3	3	2	1	3	3	2	2	2	4	2	48
157	Brand9	9	3	4	3	3	2	5	5	3	4	3	2	1	2	48
158	Brand1	1	4	4	4	4	4	4	4	4	4	4	4	4	2	27
158	Brand2	2	4	4	4	4	4	4	4	4	4	4	4	4	2	27
158	Brand3	3	4	4	4	4	4	4	4	3	3	3	3	3	2	27
158	Brand4	4	5	5	4	4	4	4	4	4	4	4	4	4	2	27
158	Brand5	5	4	3	3	3	3	3	3	3	3	3	4	4	2	27
158	Brand6	6	4	4	3	4	3	4	4	3	3	2	3	3	2	27
158	Brand4	7	4	4	4	4	4	4	4	4	4	4	4	4	2	27
158	Brand8	8	4	4	5	3	3	4	3	3	3	4	3	4	2	27
158	Brand9	9	3	3	3	3	3	3	4	3	3	4	4	3	2	27
159	Brand1	1	7	7	7	7	7	5	7	6	6	6	6	6	1	51
159	Brand2	2	6	6	6	6	6	5	7	5	5	5	5	5	1	51
159	Brand3	3	6	5	5	4	5	4	5	4	5	5	5	5	1	51
159	Brand4	4	4	4	5	3	4	3	5	4	3	2	2	2	1	51
159	Brand5	5	5	5	4	5	4	4	4	4	4	4	4	4	1	51
159	Brand6	6	6	7	6	6	6	5	7	6	5	5	5	5	1	51
159	Brand4	7	6	6	6	6	6	5	5	6	5	5	5	5	1	51
159	Brand8	8	6	6	6	6	6	5	7	6	5	5	5	5	1	51
159	Brand9	9	5	6	6	6	6	5	6	5	5	5	5	5	1	51
160	Brand1	1	6	6	6	7	7	6	6	6	2	3	3	3	2	39
160	Brand2	2	4	4	4	4	4	4	4	4	3	3	3	3	2	39
160	Brand3	3	6	6	6	6	6	6	6	6	3	3	3	3	2	39
160	Brand4	4	4	4	4	4	4	4	4	4	3	3	3	3	2	39
160	Brand5	5	5	5	5	5	4	5	5	4	3	3	3	3	2	39
160	Brand6	6	5	4	4	4	4	4	5	4	2	2	3	3	2	39
160	Brand4	7	4	4	4	4	4	4	4	4	2	2	3	3	2	39
160	Brand8	8	4	4	3	3	4	4	4	2	3	3	3	3	2	39
160	Brand9	9	5	5	5	5	5	5	5	5	2	2	2	1	2	39
161	Brand1	1	6	7	7	7	7	6	7	3	5	7	7	7	2	45
161	Brand2	2	5	6	6	6	7	6	6	2	5	6	7	7	2	45
161	Brand3	3	6	7	7	7	7	4	7	2	5	7	7	7	2	45
161	Brand4	4	3	3	4	4	4	4	4	2	4	5	5	5	2	45
161	Brand5	5	4	4	5	4	4	4	5	2	5	6	6	6	2	45
161	Brand6	6	5	4	4	4	4	4	5	2	4	5	6	6	2	45
161	Brand4	7	4	4	4	4	3	4	4	2	4	5	5	5	2	45
161	Brand8	8	4	5	5	3	3	4	4	2	5	6	6	6	2	45
161	Brand9	9	4	4	4	3	3	3	3	3	4	5	5	6	2	45
162	Brand1	1	3	3	3	4	3	3	4	3	6	5	4	4	2	29
162	Brand2	2	6	5	6	5	4	3	5	5	5	5	3	4	2	29
162	Brand3	3	4	4	3	4	3	2	3	3	4	4	3	4	2	29
162	Brand4	4	3	3	4	3	4	2	4	4	4	4	3	5	2	29
162	Brand5	5	3	4	4	4	3	3	4	3	4	4	5	4	2	29
162	Brand6	6	4	5	4	4	4	3	3	3	4	4	3	4	2	29

162	Brand4	7	4	5	4	4	4	4	5	4	5	4	4	4	2	29
162	Brand8	8	3	4	3	4	4	2	4	4	3	3	4	4	2	29
162	Brand9	9	4	4	4	4	5	2	4	4	6	5	5	4	2	29
163	Brand1	1	4	4	4	5	5	4	4	4	3	3	2	3	2	51
163	Brand2	2	4	6	5	5	5	4	7	6	4	4	4	4	2	51
163	Brand3	3	3	3	2	3	3	2	3	3	2	2	1	2	2	51
163	Brand4	4	2	2	2	2	2	2	3	2	2	2	2	2	2	51
163	Brand5	5	2	1	2	1	2	1	2	1	1	1	1	1	2	51
163	Brand6	6	2	2	2	2	2	2	2	2	2	2	2	2	2	51
163	Brand4	7	3	3	3	3	3	3	3	3	2	2	2	2	2	51
163	Brand8	8	3	3	3	3	3	3	4	3	3	3	3	3	2	51
163	Brand9	9	3	3	4	3	3	2	2	2	2	2	2	2	2	51
164	Brand1	1	5	5	5	5	5	5	5	5	4	4	3	4	2	37
164	Brand2	2	7	7	7	7	7	7	7	7	6	6	7	7	2	37
164	Brand3	3	7	7	5	5	4	7	6	5	1	3	3	2	2	37
164	Brand4	4	2	1	2	2	2	2	2	2	2	2	2	2	2	37
164	Brand5	5	4	4	4	4	4	4	4	4	2	2	2	3	2	37
164	Brand6	6	2	2	2	2	2	2	2	2	3	3	3	3	2	37
164	Brand4	7	3	3	3	3	3	3	3	3	2	2	2	2	2	37
164	Brand8	8	5	5	5	5	5	5	5	4	4	4	4	4	2	37
164	Brand9	9	1	1	1	1	1	1	1	1	2	2	2	2	2	37
165	Brand1	1	4	4	4	4	5	4	5	5	4	4	4	4	2	47
165	Brand2	2	5	5	6	5	5	6	6	5	4	4	4	4	2	47
165	Brand3	3	2	2	2	2	1	1	1	1	2	1	2	2	2	47
165	Brand4	4	2	3	3	3	3	4	5	3	3	3	3	3	2	47
165	Brand5	5	3	2	3	2	3	2	3	3	3	2	3	2	2	47
165	Brand6	6	2	3	2	3	3	2	2	2	3	2	2	3	2	47
165	Brand4	7	5	5	5	5	5	5	5	3	5	5	5	4	2	47
165	Brand8	8	3	3	3	3	5	4	4	4	2	2	2	2	2	47
165	Brand9	9	2	2	3	3	3	2	3	3	4	4	4	4	2	47
166	Brand1	1	6	6	6	6	6	6	6	6	5	5	6	6	2	57
166	Brand2	2	7	7	6	6	6	6	6	6	4	5	5	5	2	57
166	Brand3	3	6	6	5	6	6	6	6	6	4	4	4	4	2	57
166	Brand4	4	3	3	3	3	3	3	3	2	3	3	3	3	2	57
166	Brand5	5	4	4	4	4	4	4	4	4	4	4	4	4	2	57
166	Brand6	6	4	4	4	4	4	4	4	4	3	4	4	4	2	57
166	Brand4	7	4	4	4	4	4	4	4	4	4	4	4	4	2	57
166	Brand8	8	4	4	4	4	4	4	5	4	4	3	2	3	2	57
166	Brand9	9	4	4	4	4	4	4	4	4	3	3	3	3	2	57
167	Brand1	1	7	6	6	7	7	7	7	7	5	5	4	4	1	21
167	Brand2	2	5	5	5	5	5	5	5	4	3	4	3	3	1	21
167	Brand3	3	5	5	5	5	4	4	4	4	4	4	3	3	1	21
167	Brand4	4	3	4	4	3	4	3	4	3	3	3	2	2	1	21
167	Brand5	5	5	5	5	5	5	5	5	4	5	5	4	4	1	21
167	Brand6	6	5	5	5	4	4	4	4	3	4	4	4	3	1	21
167	Brand4	7	5	5	5	5	5	5	6	6	3	3	2	2	1	21
167	Brand8	8	4	4	5	4	5	5	5	4	4	4	3	3	1	21
167	Brand9	9	4	4	4	4	4	4	4	3	4	4	3	3	1	21
168	Brand1	1	4	3	3	2	3	4	3	3	3	3	3	4	2	46
168	Brand2	2	7	3	3	3	3	3	3	2	3	3	3	3	2	46
168	Brand3	3	3	5	6	6	2	4	5	6	2	1	4	2	2	46
168	Brand4	4	4	4	4	4	4	4	4	4	4	5	4	3	2	46
168	Brand5	5	3	4	5	4	7	1	3	3	3	3	4	3	2	46
168	Brand6	6	4	7	7	7	7	7	7	7	4	3	5	6	2	46

168	Brand4	7	1	1	1	1	1	1	3	2	1	1	1	1	2	46
168	Brand8	8	3	3	3	3	5	4	3	2	3	1	2	1	2	46
168	Brand9	9	1	1	1	3	7	4	4	6	1	1	1	1	2	46
169	Brand1	1	1	6	6	5	6	5	6	4	3	6	4	5	1	23
169	Brand2	2	3	3	3	4	4	4	3	3	5	5	3	4	1	23
169	Brand3	3	4	3	5	5	5	4	5	3	4	3	1	5	1	23
169	Brand4	4	5	5	5	5	5	4	5	5	5	4	3	2	1	23
169	Brand5	5	6	4	5	6	6	6	5	4	3	3	2	1	1	23
169	Brand6	6	4	3	6	6	6	6	5	3	4	5	4	4	1	23
169	Brand4	7	5	5	5	5	5	5	4	4	6	5	4	3	1	23
169	Brand8	8	4	3	3	4	4	4	5	4	4	5	4	5	1	23
169	Brand9	9	5	4	4	4	6	6	6	5	5	3	2	5	1	23
170	Brand1	1	5	4	5	6	5	4	5	4	6	5	3	5	1	29
170	Brand2	2	6	5	6	6	5	6	5	4	6	6	5	6	1	29
170	Brand3	3	6	5	4	4	5	5	5	6	6	7	4	6	1	29
170	Brand4	4	6	6	5	5	4	5	5	4	6	5	5	6	1	29
170	Brand5	5	6	6	5	4	6	5	6	6	6	5	3	5	1	29
170	Brand6	6	5	6	5	5	6	5	5	4	7	5	3	6	1	29
170	Brand4	7	7	6	4	5	6	5	4	5	5	5	6	5	1	29
170	Brand8	8	6	6	6	5	5	5	5	5	6	4	5	6	1	29
170	Brand9	9	6	5	5	6	5	6	5	4	6	4	4	4	1	29
171	Brand1	1	6	6	5	5	6	5	6	6	6	6	5	5	2	32
171	Brand2	2	5	5	4	4	4	4	4	4	6	4	5	5	2	32
171	Brand3	3	4	3	3	3	3	3	3	3	3	3	4	4	2	32
171	Brand4	4	3	3	3	3	3	2	2	2	4	3	3	3	2	32
171	Brand5	5	5	6	5	5	5	5	5	6	5	4	5	5	2	32
171	Brand6	6	5	5	5	6	6	5	5	6	5	5	5	4	2	32
171	Brand4	7	5	5	5	5	5	5	5	5	5	5	5	4	2	32
171	Brand8	8	6	6	5	5	6	5	5	6	6	5	5	5	2	32
171	Brand9	9	6	6	5	5	5	5	6	6	5	5	4	5	2	32
172	Brand1	1	4	3	4	4	4	3	4	4	2	2	1	2	1	62
172	Brand2	2	5	5	5	4	5	4	4	3	3	2	1	3	1	62
172	Brand3	3	3	3	3	3	4	3	4	3	2	2	1	3	1	62
172	Brand4	4	1	1	1	1	1	1	1	1	1	1	1	1	1	62
172	Brand5	5	2	2	2	2	2	2	2	2	2	2	2	2	1	62
172	Brand6	6	2	2	2	2	2	2	2	1	2	2	2	1	1	62
172	Brand4	7	1	1	1	1	1	1	1	1	1	1	1	1	1	62
172	Brand8	8	2	2	2	2	2	2	2	1	1	1	1	1	1	62
172	Brand9	9	2	2	2	2	2	2	2	1	1	1	1	1	1	62
173	Brand1	1	4	5	4	4	4	4	4	4	4	4	5	4	1	59
173	Brand2	2	4	4	5	4	4	5	5	4	5	4	4	4	1	59
173	Brand3	3	2	2	2	1	1	1	1	1	3	2	2	2	1	59
173	Brand4	4	4	4	4	4	4	5	4	4	4	4	4	5	1	59
173	Brand5	5	6	6	6	6	5	4	6	6	5	5	5	5	1	59
173	Brand6	6	5	6	5	5	5	5	5	4	4	4	3	4	1	59
173	Brand4	7	6	6	5	6	6	5	6	5	5	4	4	4	1	59
173	Brand8	8	4	5	4	4	4	5	4	4	4	5	5	4	1	59
173	Brand9	9	4	4	4	5	4	5	4	4	4	5	4	4	1	59
174	Brand1	1	6	6	7	7	7	7	6	4	5	5	5	5	1	58
174	Brand2	2	6	6	6	6	6	5	6	4	4	4	5	5	1	58
174	Brand3	3	6	5	5	5	6	6	5	5	6	6	6	6	1	58
174	Brand4	4	4	4	1	4	1	2	3	1	2	2	2	2	1	58
174	Brand5	5	3	3	3	2	3	3	4	2	3	3	3	4	1	58
174	Brand6	6	3	3	3	3	2	2	3	2	2	3	3	3	1	58

174	Brand4	7	3	2	3	3	2	1	2	2	2	2	2	2	1	58
174	Brand8	8	6	6	6	7	7	5	7	7	6	6	6	7	1	58
174	Brand9	9	1	2	2	1	2	2	1	1	2	2	2	1	1	58
175	Brand1	1	6	5	5	5	5	5	5	5	2	3	3	3	2	31
175	Brand2	2	4	4	4	4	4	4	4	4	2	2	2	2	2	31
175	Brand3	3	5	5	4	5	5	5	4	4	3	3	3	3	2	31
175	Brand4	4	3	3	3	3	3	3	3	3	3	3	3	3	2	31
175	Brand5	5	4	4	3	3	3	4	3	4	3	3	3	3	2	31
175	Brand6	6	4	4	4	4	4	4	4	4	3	3	3	3	2	31
175	Brand4	7	4	5	3	3	2	3	3	4	1	2	3	2	2	31
175	Brand8	8	3	3	3	3	3	3	3	3	3	3	3	3	2	31
175	Brand9	9	3	3	3	3	3	3	3	3	3	3	3	3	2	31
176	Brand1	1	4	3	4	3	3	2	2	4	4	4	1	2	1	50
176	Brand2	2	4	4	2	3	3	3	4	4	4	3	5	4	1	50
176	Brand3	3	4	4	4	5	2	3	3	4	6	4	3	4	1	50
176	Brand4	4	4	4	3	3	6	4	5	4	3	4	4	4	1	50
176	Brand5	5	4	4	3	6	4	4	3	2	4	4	4	4	1	50
176	Brand6	6	4	3	4	4	4	2	3	3	4	3	3	4	1	50
176	Brand4	7	3	4	4	3	3	3	3	3	3	4	3	4	1	50
176	Brand8	8	3	4	3	4	4	3	4	4	3	3	4	3	1	50
176	Brand9	9	4	4	3	4	2	2	2	3	3	3	3	4	1	50
177	Brand1	1	7	7	7	7	7	6	6	5	4	6	5	6	2	33
177	Brand2	2	5	6	6	7	6	6	7	6	5	5	6	5	2	33
177	Brand3	3	2	2	1	1	1	2	1	1	4	5	5	3	2	33
177	Brand4	4	4	4	5	4	3	4	4	3	3	3	5	3	2	33
177	Brand5	5	5	4	5	4	5	4	6	5	3	3	4	5	2	33
177	Brand6	6	5	5	4	4	4	4	5	5	4	4	4	4	2	33
177	Brand4	7	4	4	4	4	4	4	4	4	3	4	4	4	2	33
177	Brand8	8	4	5	5	5	4	4	5	4	4	4	4	5	2	33
177	Brand9	9	3	2	3	3	3	4	3	2	3	4	4	4	2	33
178	Brand1	1	1	1	1	1	1	4	1	1	1	1	4	1	1	44
178	Brand2	2	1	1	1	1	1	1	1	1	1	1	1	1	1	44
178	Brand3	3	1	1	1	1	1	1	1	1	3	3	1	1	1	44
178	Brand4	4	1	1	1	1	1	1	1	1	1	1	1	1	1	44
178	Brand5	5	1	1	1	1	1	1	1	1	1	1	1	1	1	44
178	Brand6	6	1	1	1	1	1	1	1	1	1	1	1	1	1	44
178	Brand4	7	1	1	1	1	1	1	1	1	1	1	1	1	1	44
178	Brand8	8	1	1	1	1	1	1	1	1	1	1	1	1	1	44
178	Brand9	9	1	1	1	1	1	1	1	1	1	1	1	1	1	44
179	Brand1	1	4	3	4	3	3	4	3	4	4	3	4	5	1	37
179	Brand2	2	3	4	3	4	3	4	4	3	4	4	3	4	1	37
179	Brand3	3	4	3	4	4	3	5	4	3	3	4	3	4	1	37
179	Brand4	4	4	4	5	4	3	3	4	4	3	4	3	4	1	37
179	Brand5	5	3	4	3	4	3	4	4	4	4	3	4	4	1	37
179	Brand6	6	3	4	4	3	4	4	3	5	3	4	3	4	1	37
179	Brand4	7	4	3	4	4	4	5	3	3	4	4	2	4	1	37
179	Brand8	8	4	3	1	1	1	1	1	1	4	2	3	3	1	37
179	Brand9	9	4	3	4	4	4	3	5	3	4	4	3	4	1	37
180	Brand1	1	6	6	6	6	6	5	6	5	2	2	2	2	2	48
180	Brand2	2	5	5	4	5	4	4	5	4	2	2	2	2	2	48
180	Brand3	3	5	5	5	5	4	4	5	4	2	2	2	2	2	48
180	Brand4	4	3	3	3	2	3	3	3	2	2	2	2	2	2	48
180	Brand5	5	4	4	3	3	4	4	4	3	2	2	2	2	2	48
180	Brand6	6	4	4	4	4	3	4	4	2	2	2	2	2	2	48

180	Brand4	7	4	4	4	4	4	4	5	3	2	2	2	2	2	48
180	Brand8	8	4	4	4	4	4	4	4	3	2	2	2	2	2	48
180	Brand9	9	3	3	3	3	3	3	3	2	2	2	2	2	2	48
181	Brand1	1	4	5	4	5	5	5	5	5	5	5	5	5	2	37
181	Brand2	2	6	6	6	6	5	5	6	6	6	5	5	6	2	37
181	Brand3	3	5	4	5	4	4	4	4	4	4	4	4	4	2	37
181	Brand4	4	6	6	6	6	6	4	7	6	5	6	4	6	2	37
181	Brand5	5	5	6	5	6	4	4	4	4	5	5	4	5	2	37
181	Brand6	6	5	6	5	6	5	5	5	5	5	6	4	6	2	37
181	Brand4	7	6	6	6	6	5	5	5	5	4	5	5	5	2	37
181	Brand8	8	5	5	5	6	5	4	5	5	5	6	4	5	2	37
181	Brand9	9	5	6	6	6	5	4	6	5	5	5	4	5	2	37
182	Brand1	1	4	4	4	3	3	3	4	3	3	3	3	3	2	28
182	Brand2	2	2	3	3	2	3	3	3	3	2	2	2	3	2	28
182	Brand3	3	2	3	2	2	2	3	3	3	1	2	2	2	2	28
182	Brand4	4	4	3	2	3	3	3	3	3	3	3	3	3	2	28
182	Brand5	5	3	2	3	2	2	2	3	2	2	3	3	3	2	28
182	Brand6	6	1	1	1	1	1	1	1	1	1	1	1	1	2	28
182	Brand4	7	4	4	4	4	4	4	4	4	3	4	4	4	2	28
182	Brand8	8	6	6	6	6	6	6	7	7	5	5	5	6	2	28
182	Brand9	9	3	3	3	3	3	3	3	3	2	3	2	3	2	28
183	Brand1	1	1	3	1	1	1	1	3	1	1	3	3	3	2	33
183	Brand2	2	4	4	1	1	1	3	3	1	1	3	3	3	2	33
183	Brand3	3	4	4	4	4	4	4	4	1	1	3	3	3	2	33
183	Brand4	4	1	1	1	1	1	1	1	1	1	3	3	3	2	33
183	Brand5	5	1	4	3	1	1	1	3	1	3	1	1	1	2	33
183	Brand6	6	1	5	1	1	1	1	4	1	1	3	3	3	2	33
183	Brand4	7	1	5	1	1	4	1	1	1	1	3	3	3	2	33
183	Brand8	8	3	5	4	4	5	5	5	1	1	3	3	3	2	33
183	Brand9	9	1	5	1	1	3	1	1	1	1	3	3	3	2	33
184	Brand1	1	3	3	3	3	3	3	3	2	2	2	2	3	2	36
184	Brand2	2	3	3	3	3	3	3	3	3	3	3	2	2	2	36
184	Brand3	3	2	2	2	2	2	2	2	2	2	2	2	2	2	36
184	Brand4	4	3	3	3	4	4	4	3	3	3	3	3	3	2	36
184	Brand5	5	3	3	3	3	3	3	3	3	3	2	2	3	2	36
184	Brand6	6	2	2	2	2	2	2	2	2	2	2	2	2	2	36
184	Brand4	7	4	4	4	3	3	3	3	4	3	3	3	3	2	36
184	Brand8	8	4	4	4	4	2	2	3	3	3	3	3	3	2	36
184	Brand9	9	3	4	3	3	3	4	3	3	3	3	3	3	2	36
185	Brand1	1	6	6	5	6	5	5	5	5	4	4	4	4	1	47
185	Brand2	2	5	5	4	5	4	4	5	4	3	3	3	4	1	47
185	Brand3	3	5	4	5	5	4	4	5	4	3	3	3	4	1	47
185	Brand4	4	4	4	4	4	4	4	4	4	3	3	3	4	1	47
185	Brand5	5	4	4	4	4	4	4	5	4	3	3	3	4	1	47
185	Brand6	6	5	5	4	5	4	4	5	4	3	3	3	4	1	47
185	Brand4	7	4	4	4	4	4	4	4	4	3	3	3	4	1	47
185	Brand8	8	4	4	4	4	4	4	4	4	3	3	3	4	1	47
185	Brand9	9	4	4	4	4	4	4	4	4	2	4	3	4	1	47
186	Brand1	1	3	4	4	4	5	4	4	1	4	2	3	2	1	40
186	Brand2	2	3	4	5	4	3	4	3	4	3	3	3	3	1	40
186	Brand3	3	2	2	2	2	2	3	4	5	3	3	3	2	1	40
186	Brand4	4	1	2	1	2	2	3	1	2	2	3	2	1	1	40
186	Brand5	5	5	5	6	5	6	6	5	6	4	5	3	5	1	40
186	Brand6	6	6	5	4	6	5	4	5	4	3	4	4	3	1	40

186	Brand4	7	4	5	5	5	7	7	7	5	4	5	4	5	1	40
186	Brand8	8	5	4	5	4	4	4	5	5	3	4	5	4	1	40
186	Brand9	9	4	5	4	4	5	4	5	5	3	5	4	3	1	40
187	Brand1	1	5	5	5	5	5	5	5	5	3	4	4	4	1	42
187	Brand2	2	4	5	4	5	4	4	5	4	3	4	3	3	1	42
187	Brand3	3	3	4	3	3	3	4	4	4	3	3	4	4	1	42
187	Brand4	4	4	4	4	3	4	4	4	3	3	3	2	3	1	42
187	Brand5	5	3	4	3	3	3	3	3	3	3	4	3	4	1	42
187	Brand6	6	4	4	4	3	3	4	4	4	3	3	3	3	1	42
187	Brand4	7	4	4	4	4	4	4	3	4	3	3	3	2	1	42
187	Brand8	8	3	5	4	4	5	4	5	4	4	3	3	3	1	42
187	Brand9	9	4	4	4	3	3	4	3	3	4	3	4	4	1	42
188	Brand1	1	5	5	5	6	6	6	6	5	3	3	4	3	1	41
188	Brand2	2	3	3	3	2	3	3	2	3	2	3	2	2	1	41
188	Brand3	3	3	3	2	2	2	3	2	3	2	3	2	3	1	41
188	Brand4	4	2	2	2	3	2	2	2	1	2	3	3	3	1	41
188	Brand5	5	3	3	3	2	3	2	3	2	2	2	2	2	1	41
188	Brand6	6	2	2	2	2	3	3	2	2	2	3	2	2	1	41
188	Brand4	7	2	2	2	2	2	2	2	2	2	2	2	2	1	41
188	Brand8	8	1	2	2	2	2	2	1	2	2	2	2	2	1	41
188	Brand9	9	4	5	4	3	4	3	3	3	3	3	4	3	1	41
189	Brand1	1	5	5	4	4	4	2	4	4	2	2	2	2	1	31
189	Brand2	2	2	3	2	2	2	2	2	2	2	2	2	1	1	31
189	Brand3	3	3	3	3	3	3	3	3	3	2	2	2	2	1	31
189	Brand4	4	2	2	2	2	2	2	2	2	2	2	2	2	1	31
189	Brand5	5	3	3	3	3	3	2	3	3	2	2	2	2	1	31
189	Brand6	6	5	4	4	4	4	3	4	4	2	2	2	2	1	31
189	Brand4	7	5	4	4	4	4	4	5	4	2	2	2	2	1	31
189	Brand8	8	4	4	4	4	4	3	4	4	2	2	2	2	1	31
189	Brand9	9	3	2	2	2	2	2	3	3	2	2	2	2	1	31
190	Brand1	1	6	6	5	6	6	4	5	6	6	5	5	5	2	29
190	Brand2	2	3	4	4	4	4	5	4	3	3	4	4	4	2	29
190	Brand3	3	4	4	4	4	4	4	4	4	4	4	4	4	2	29
190	Brand4	4	3	3	3	3	3	4	4	4	3	4	4	4	2	29
190	Brand5	5	4	4	4	4	4	4	4	4	4	4	4	4	2	29
190	Brand6	6	3	3	4	4	5	4	5	3	3	4	4	4	2	29
190	Brand4	7	4	4	4	4	4	4	4	4	3	4	4	4	2	29
190	Brand8	8	3	4	4	4	4	4	4	4	4	4	4	4	2	29
190	Brand9	9	4	4	4	4	4	4	4	4	3	4	4	4	2	29
191	Brand1	1	4	4	5	5	4	3	6	4	4	4	4	4	1	54
191	Brand2	2	3	3	3	2	2	2	2	2	3	2	3	3	1	54
191	Brand3	3	4	4	4	4	5	3	5	4	4	4	5	4	1	54
191	Brand4	4	4	4	4	4	3	2	2	1	3	5	4	3	1	54
191	Brand5	5	3	3	3	3	3	3	3	3	2	2	2	2	1	54
191	Brand6	6	3	3	3	4	3	3	4	3	3	3	3	3	1	54
191	Brand4	7	5	4	3	3	3	3	5	3	3	3	2	3	1	54
191	Brand8	8	2	2	2	2	3	2	5	2	3	3	3	3	1	54
191	Brand9	9	5	4	3	3	4	3	6	3	3	3	4	3	1	54
192	Brand1	1	5	4	4	4	7	5	4	4	2	4	3	4	2	27
192	Brand2	2	7	6	7	7	7	7	7	7	2	5	5	6	2	27
192	Brand3	3	2	2	1	2	1	2	1	1	2	2	1	2	2	27
192	Brand4	4	1	4	3	3	3	3	3	2	1	3	3	3	2	27
192	Brand5	5	3	4	3	4	3	2	3	3	2	2	3	2	2	27
192	Brand6	6	3	4	3	3	3	4	2	2	4	3	3	2	2	27

192	Brand4	7	5	5	5	3	4	3	3	2	1	3	5	3	2	27
192	Brand8	8	4	6	5	1	4	3	1	1	3	4	1	3	2	27
192	Brand9	9	4	4	3	5	4	4	4	3	3	4	3	3	2	27
193	Brand1	1	4	5	5	4	4	4	5	4	3	4	4	4	1	43
193	Brand2	2	5	5	5	4	5	4	5	4	3	3	4	4	1	43
193	Brand3	3	5	4	5	4	4	4	5	4	3	4	4	4	1	43
193	Brand4	4	4	4	5	4	4	4	3	4	3	4	4	3	1	43
193	Brand5	5	4	4	4	4	4	4	4	4	3	4	4	3	1	43
193	Brand6	6	4	4	4	4	4	4	5	4	3	4	4	3	1	43
193	Brand4	7	4	4	6	4	4	4	4	4	3	3	4	3	1	43
193	Brand8	8	4	4	4	4	4	4	4	4	3	4	4	3	1	43
193	Brand9	9	3	4	3	4	3	3	4	3	3	3	4	3	1	43
194	Brand1	1	4	4	4	4	4	3	4	4	3	3	3	3	2	33
194	Brand2	2	3	3	3	3	3	3	3	3	3	3	3	3	2	33
194	Brand3	3	4	4	4	4	3	3	4	3	3	3	3	3	2	33
194	Brand4	4	3	3	4	3	3	3	4	3	3	3	3	3	2	33
194	Brand5	5	1	4	4	4	4	4	4	4	3	3	3	4	2	33
194	Brand6	6	3	3	4	3	3	3	3	3	3	3	3	3	2	33
194	Brand4	7	4	4	4	4	4	4	4	4	3	3	3	3	2	33
194	Brand8	8	3	3	4	3	3	3	3	3	3	3	3	3	2	33
194	Brand9	9	4	3	3	3	4	3	3	3	3	3	3	3	2	33
195	Brand1	1	7	7	7	7	7	7	7	7	1	1	1	1	1	35
195	Brand2	2	4	4	4	4	4	4	4	4	1	1	1	1	1	35
195	Brand3	3	5	5	5	5	5	5	5	5	1	1	1	1	1	35
195	Brand4	4	1	1	1	1	1	1	1	1	1	1	1	1	1	35
195	Brand5	5	3	3	3	3	3	3	3	3	1	1	1	1	1	35
195	Brand6	6	7	7	7	7	7	7	7	7	1	1	1	1	1	35
195	Brand4	7	3	3	3	3	3	3	3	3	1	1	1	1	1	35
195	Brand8	8	3	3	3	3	3	3	3	3	1	1	1	1	1	35
195	Brand9	9	2	2	2	2	2	2	2	2	1	1	1	1	1	35
196	Brand1	1	4	3	3	3	3	3	4	3	3	4	3	3	2	37
196	Brand2	2	3	3	3	3	3	3	4	3	3	3	3	3	2	37
196	Brand3	3	4	3	4	4	4	3	4	3	3	3	3	3	2	37
196	Brand4	4	3	3	3	3	3	3	3	3	2	3	3	3	2	37
196	Brand5	5	3	3	3	3	3	3	4	3	3	3	3	3	2	37
196	Brand6	6	3	3	3	3	3	3	3	3	3	3	3	3	2	37
196	Brand4	7	2	3	3	3	3	3	3	3	3	3	3	3	2	37
196	Brand8	8	3	3	3	3	3	3	4	3	3	3	3	3	2	37
196	Brand9	9	3	3	3	3	3	3	3	3	3	3	3	3	2	37
197	Brand1	1	7	6	6	7	7	6	7	1	1	1	1	1	1	27
197	Brand2	2	5	5	5	6	7	5	5	1	1	1	1	1	1	27
197	Brand3	3	6	5	5	6	6	6	5	3	1	1	1	1	1	27
197	Brand4	4	1	1	1	1	1	1	1	1	1	1	1	1	1	27
197	Brand5	5	7	7	6	6	6	6	6	3	1	1	1	1	1	27
197	Brand6	6	1	1	1	1	1	1	1	1	1	1	1	2	1	27
197	Brand4	7	1	1	1	1	1	4	2	1	1	1	1	1	1	27
197	Brand8	8	6	6	6	7	7	5	6	4	1	1	1	2	1	27
197	Brand9	9	1	1	1	1	2	1	1	1	1	1	1	1	1	27
198	Brand1	1	7	7	6	7	7	7	7	7	6	5	5	5	1	31
198	Brand2	2	6	6	4	5	5	6	5	4	5	5	3	4	1	31
198	Brand3	3	4	5	3	5	4	1	1	4	5	5	4	5	1	31
198	Brand4	4	6	7	6	6	6	4	6	6	6	6	5	6	1	31
198	Brand5	5	4	4	3	4	3	3	5	3	2	2	1	2	1	31
198	Brand6	6	6	7	7	7	7	7	7	7	5	5	4	5	1	31

198	Brand4	7	5	6	6	6	6	6	6	7	4	4	4	4	1	31
198	Brand8	8	6	7	7	7	7	7	7	6	5	5	4	5	1	31
198	Brand9	9	4	2	1	2	3	3	3	2	5	3	2	3	1	31
199	Brand1	1	5	5	5	5	5	5	5	5	2	2	2	2	2	68
199	Brand2	2	5	4	4	4	4	4	4	4	2	2	2	2	2	68
199	Brand3	3	4	4	4	4	4	4	4	4	2	2	2	2	2	68
199	Brand4	4	3	3	3	3	3	3	3	3	2	2	2	2	2	68
199	Brand5	5	4	4	4	4	4	4	4	4	2	2	2	2	2	68
199	Brand6	6	6	6	6	6	6	6	6	6	2	2	2	2	2	68
199	Brand4	7	4	4	4	4	4	4	4	4	2	2	2	2	2	68
199	Brand8	8	5	5	5	5	5	5	5	5	2	2	2	2	2	68
199	Brand9	9	3	3	3	3	3	3	3	3	2	2	2	2	2	68
200	Brand1	1	5	6	6	6	4	6	6	5	6	6	6	5	2	24
200	Brand2	2	6	6	6	7	7	7	6	6	5	6	5	5	2	24
200	Brand3	3	7	6	7	6	7	7	7	6	5	6	6	6	2	24
200	Brand4	4	6	5	4	4	3	5	4	4	5	6	4	3	2	24
200	Brand5	5	7	7	6	7	7	7	7	7	6	6	5	5	2	24
200	Brand6	6	6	6	5	6	6	5	5	6	5	5	5	6	2	24
200	Brand4	7	6	5	6	5	5	5	5	5	5	6	5	4	2	24
200	Brand8	8	6	7	6	7	7	6	7	7	5	6	6	7	2	24
200	Brand9	9	5	5	6	4	4	4	5	4	5	4	5	5	2	24
201	Brand1	1	5	5	5	6	6	6	5	6	6	5	5	6	1	31
201	Brand2	2	7	7	7	7	7	6	6	5	6	6	7	7	1	31
201	Brand3	3	6	6	6	6	6	5	5	6	5	5	5	3	1	31
201	Brand4	4	6	6	6	7	6	6	7	5	6	6	6	5	1	31
201	Brand5	5	7	6	4	6	6	6	7	6	5	5	5	3	1	31
201	Brand6	6	5	6	6	6	7	6	6	6	6	5	6	5	1	31
201	Brand4	7	6	7	6	6	6	7	6	6	6	6	5	6	1	31
201	Brand8	8	7	6	4	6	6	6	6	6	6	6	4	6	1	31
201	Brand9	9	6	5	6	6	5	6	6	5	6	6	4	3	1	31
202	Brand1	1	5	6	4	5	5	5	4	4	3	5	5	5	1	31
202	Brand2	2	5	5	4	5	4	5	4	5	4	5	4	4	1	31
202	Brand3	3	2	2	2	2	2	2	2	2	2	1	2	4	1	31
202	Brand4	4	4	6	6	4	4	5	6	5	5	5	4	5	1	31
202	Brand5	5	4	3	3	3	4	3	5	3	4	3	3	3	1	31
202	Brand6	6	5	5	6	5	5	4	5	5	3	3	4	4	1	31
202	Brand4	7	4	3	5	4	3	5	4	4	3	3	3	3	1	31
202	Brand8	8	4	4	5	4	4	4	5	5	3	3	4	3	1	31
202	Brand9	9	4	3	3	4	3	4	5	3	3	3	3	3	1	31
203	Brand1	1	5	6	6	4	5	5	6	5	5	5	5	6	2	28
203	Brand2	2	5	5	5	7	5	5	4	5	5	4	5	5	2	28
203	Brand3	3	6	6	5	5	6	5	5	5	5	5	6	5	2	28
203	Brand4	4	5	6	6	5	5	5	4	4	5	5	5	3	2	28
203	Brand5	5	6	6	6	6	7	6	6	6	6	6	7	6	2	28
203	Brand6	6	6	6	6	6	7	5	5	5	6	6	6	6	2	28
203	Brand4	7	6	6	6	6	7	6	1	6	5	4	6	7	2	28
203	Brand8	8	4	5	5	6	5	5	4	5	4	5	5	5	2	28
203	Brand9	9	5	5	7	5	5	3	4	5	5	5	6	7	2	28
204	Brand1	1	5	5	4	5	4	4	5	4	4	4	5	4	2	29
204	Brand2	2	5	5	4	4	4	4	6	4	3	4	4	4	2	29
204	Brand3	3	4	4	4	3	4	3	3	3	3	5	4	4	2	29
204	Brand4	4	4	4	4	4	4	4	4	4	3	4	4	4	2	29
204	Brand5	5	4	4	4	4	3	5	4	4	4	4	4	4	2	29
204	Brand6	6	6	6	4	4	4	4	4	4	4	3	5	4	2	29

204	Brand4	7	4	5	4	4	4	4	4	4	3	4	4	4	2	29
204	Brand8	8	5	6	4	5	4	4	5	4	4	4	5	4	2	29
204	Brand9	9	3	4	4	4	4	5	4	5	4	3	4	5	2	29
205	Brand1	1	2	2	3	2	3	3	2	3	4	4	4	3	2	53
205	Brand2	2	5	6	6	6	5	2	6	6	4	4	4	4	2	53
205	Brand3	3	4	4	4	3	3	3	4	4	3	3	3	3	2	53
205	Brand4	4	3	3	3	2	2	2	3	3	2	2	2	2	2	53
205	Brand5	5	2	2	2	2	1	1	1	1	2	2	2	2	2	53
205	Brand6	6	2	2	2	2	1	1	2	1	1	1	1	1	2	53
205	Brand4	7	3	2	2	3	3	2	2	1	1	1	1	1	2	53
205	Brand8	8	2	2	2	2	1	1	1	1	2	2	2	2	2	53
205	Brand9	9	3	3	3	3	3	3	1	1	1	1	1	1	2	53
206	Brand1	1	4	4	3	3	3	3	3	2	5	3	3	3	2	44
206	Brand2	2	3	3	3	3	3	3	3	3	5	3	2	2	2	44
206	Brand3	3	4	3	3	3	3	3	2	2	5	3	3	2	2	44
206	Brand4	4	2	2	2	2	2	2	2	2	5	3	3	2	2	44
206	Brand5	5	2	2	2	2	2	2	2	2	5	3	3	2	2	44
206	Brand6	6	4	4	3	4	4	3	3	3	5	3	3	3	2	44
206	Brand4	7	3	3	2	2	2	3	3	2	3	3	2	2	2	44
206	Brand8	8	2	2	2	2	2	2	2	2	5	3	2	2	2	44
206	Brand9	9	2	2	2	2	2	2	2	2	5	3	3	2	2	44
