# Supplementary material for: Effects of brand and brand trust on initial trust in fully automated driving system
Source: PLoS One. 2023 May 4;18(5):e0284654. doi: 10.1371/journal.pone.0284654 (PMC10159113; doi:10.1371/journal.pone.0284654)
Supplement: Attachment — Submitted filename: PONE-D-22-29912_reviewer.pdf [file pone.0284654.s002.pdf]

PLOS ONE

Effects of brand and brand trust on initial trust in fully automated driving system

--Manuscript Draft--

**Manuscript Number:**	PONE-D-22-29912
**Article Type:**	Research Article
**Full Title:**	Effects of brand and brand trust on initial trust in fully automated driving system
**Short Title:**	Brand, brand trust and initial trust in fully automated driving system
**Corresponding Author:**	Zixin Cui<br>University of Tsukuba: Tsukuba Daigaku<br>tsukuba, JAPAN
**Keywords:**	trust in automation; initial trust; full driving automation; automobile brand; brand trust; cognitive structure; structural equation model; free word association test; word cloud visualization
**Abstract:**	Before fully automated driving system (FADS) is put into practical use, one issue is how to calibrate drivers' initial trust in FADS to an appropriate degree to avoid inappropriate disuse or improper use. This paper aims to identify the factors that affect drivers' initial trust in FADS. Two online surveys were conducted. One was to explore the effects of automobile brand and drivers' trust in automobile brand on drivers' initial trust in FADS with a structural equation model (SEM). The other was to identify drivers' cognitive structures on automobile brands with the free word association test (FWAT) and summary the characteristics of drivers' cognitive structures on automobile brands that can win drivers' higher initial trust in FADS. The results showed that 1) drivers' trust in automobile brand positively impacts drivers' initial trust in FADS; 2) the degree of drivers' initial trust in FADS was significantly different across different automobile brands; 3) For automobile brands highly trusted in, drivers possess more rich and various cognitive structures with some particular characteristics. These findings suggest the necessity of considering the influence of automobile brands in calibrating drivers' initial trust in driving automation.
**Order of Authors:**	Zixin Cui
	Nianzhi Tu
	Makoto Itoh
**Additional Information:**	
**Question**	**Response**
**Financial Disclosure**<br><br>Enter a financial disclosure statement that describes the sources of funding for the work included in this submission. Review the submission guidelines for detailed requirements. View published research articles from *PLOS ONE* for specific examples.<br><br>This statement is required for submission and **will appear in the published article** if the submission is accepted. Please make sure it is accurate.	This study was supported by the Japan Society for the Promotion of Science (https://www.jsps.go.jp/) KAKENHI [grant number 17KT0153] awarded to M.I. The funders had no role in study design, data collection and analysis, decision to publish, or preparation of the manuscript.

Powered by Editorial Manager® and ProduXion Manager® from Aries Systems Corporation

**Unfunded studies**<br>Enter: *The author(s) received no specific funding for this work.*<br><br>**Funded studies**<br>Enter a statement with the following details:<br>• Initials of the authors who received each award<br>• Grant numbers awarded to each author<br>• The full name of each funder<br>• URL of each funder website<br>• Did the sponsors or funders play any role in the study design, data collection and analysis, decision to publish, or preparation of the manuscript?<br>• **NO** - Include this sentence at the end of your statement: *The funders had no role in study design, data collection and analysis, decision to publish, or preparation of the manuscript.*<br>• **YES** - Specify the role(s) played.<br><br>* typeset	
**Competing Interests**<br><br>Use the instructions below to enter a competing interest statement for this submission. On behalf of all authors, disclose any competing interests that could be perceived to bias this work—acknowledging all financial support and any other relevant financial or non-financial competing interests.<br><br>This statement is required for submission and **will appear in the published article** if the submission is accepted. Please make sure it is accurate and that any funding sources listed in your Funding Information later in the submission form are also declared in your Financial Disclosure statement.<br><br>View published research articles from *PLOS ONE* for specific examples.	The authors have declared that no competing interests exist.

**NO authors have competing interests**<br><br>Enter: *The authors have declared that no competing interests exist.*<br><br>**Authors with competing interests**<br><br>Enter competing interest details beginning with this statement:<br><br>*I have read the journal's policy and the authors of this manuscript have the following competing interests: [insert competing interests here]*<br><br>* typeset	
**Ethics Statement**<br><br>Enter an ethics statement for this submission. This statement is required if the study involved:<br><br>• Human participants<br>• Human specimens or tissue<br>• Vertebrate animals or cephalopods<br>• Vertebrate embryos or tissues<br>• Field research<br><br>Write "N/A" if the submission does not require an ethics statement.<br><br>General guidance is provided below. Consult the submission guidelines for detailed instructions. **Make sure that all information entered here is included in the Methods section of the manuscript.**	This study was written approved by the ethical review board of the Faculty of Engineering, Information, and Systems at the University of Tsukuba (Approval number 2020R369) .

Powered by Editorial Manager® and ProduXion Manager® from Aries Systems Corporation

**Format for specific study types**<br><br>**Human Subject Research (involving human participants and/or tissue)**<br>• Give the name of the institutional review board or ethics committee that approved the study<br>• Include the approval number and/or a statement indicating approval of this research<br>• Indicate the form of consent obtained (written/oral) or the reason that consent was not obtained (e.g. the data were analyzed anonymously)<br><br>**Animal Research (involving vertebrate animals, embryos or tissues)**<br>• Provide the name of the Institutional Animal Care and Use Committee (IACUC) or other relevant ethics board that reviewed the study protocol, and indicate whether they approved this research or granted a formal waiver of ethical approval<br>• Include an approval number if one was obtained<br>• If the study involved *non-human primates*, add *additional details* about animal welfare and steps taken to ameliorate suffering<br>• If anesthesia, euthanasia, or any kind of animal sacrifice is part of the study, include briefly which substances and/or methods were applied<br><br>**Field Research**<br><br>Include the following details if this study involves the collection of plant, animal, or other materials from a natural setting:<br>• Field permit number<br>• Name of the institution or relevant body that granted permission	
**Data Availability**<br><br>Authors are required to make all data underlying the findings described fully available, without restriction, and from the time of publication. PLOS allows rare exceptions to address legal and ethical concerns. See the PLOS Data Policy and FAQ for detailed information.	No - some restrictions will apply

Powered by Editorial Manager® and ProduXion Manager® from Aries Systems Corporation

A Data Availability Statement describing where the data can be found is required at submission. Your answers to this question constitute the Data Availability Statement and **will be published in the article**, if accepted.<br><br>**Important:** Stating 'data available on request from the author' is not sufficient. If your data are only available upon request, select 'No' for the first question and explain your exceptional situation in the text box.<br><br>Do the authors confirm that all data underlying the findings described in their manuscript are fully available without restriction?	
**Describe where the data may be found in full sentences. If you are copying our sample text, replace any instances of XXX with the appropriate details.**<br><br>• If the data are **held or will be held in a public repository**, include URLs, accession numbers or DOIs. If this information will only be available after acceptance, indicate this by ticking the box below. For example: *All XXX files are available from the XXX database (accession number(s) XXX, XXX.).*<br>• If the data are all contained **within the manuscript and/or Supporting Information files**, enter the following: *All relevant data are within the manuscript and its Supporting Information files.*<br>• If neither of these applies but you are able to provide **details of access elsewhere**, with or without limitations, please do so. For example:<br><br>*Data cannot be shared publicly because of [XXX]. Data are available from the XXX Institutional Data Access / Ethics Committee (contact via XXX) for researchers who meet the criteria for access to confidential data.*<br><br>*The data underlying the results presented in the study are available from (include the name of the third party*	Some data in this study cannot be shared publicly because data contain potentially identifying or sensitive brand and consumer information. Data are available from the Laboratory for Cognitive Systems Science, Faculty of Engineering, Information, and Systems at the University of Tsukuba (contact via question@css.risk.tsukuba.ac.jp) for researchers who meet the criteria for access to confidential data.

and contact information or URL).<br>• This text is appropriate if the data are owned by a third party and authors do not have permission to share the data.<br><br>* typeset	
Additional data availability information:	

Manuscript

Click here to access/download;Manuscript;manuscript_submitted.docx

Effects of brand and brand trust on initial trust in fully automated driving system

Zixin Cui[1*], Nianzhi Tu[1], Makoto Itoh[2]

[1] Department of Risk Engineering, Graduate School of System and Information Engineering, University of Tsukuba, Tsukuba, Ibaraki, Japan,

[2] Institute of Systems and Information Engineering, University of Tsukuba, Tsukuba, Ibaraki, Japan

*cui@css.risk.tsukuba.ac.jp(ZC)

Abstract

Before fully automated driving system (FADS) is put into practical use, one issue is how to calibrate drivers' initial trust in FADS to an appropriate degree to avoid inappropriate disuse or improper use. This paper aims to identify the factors that affect drivers' initial trust in FADS. Two online surveys were conducted. One was to explore the effects of automobile brand and drivers' trust in automobile brand on drivers' initial trust in FADS with a structural equation model (SEM). The other was to identify drivers' cognitive structures on automobile brands with the free word association test (FWAT) and summary the characteristics of drivers' cognitive structures on automobile brands that can win drivers' higher initial trust in FADS. The results showed that 1) drivers' trust in automobile brand positively impacts drivers' initial trust in FADS; 2) the degree of drivers' initial trust in FADS was significantly different across different automobile brands; 3) For automobile brands highly trusted in, drivers possess more rich and various cognitive structures with some particular characteristics. These findings suggest the necessity of considering the influence of automobile brands in calibrating drivers' initial trust in driving automation.

Introduction

The practical application of Fully Automated Driving System (FADS) is expected, since it can optimize the traffic environment and help to decrease the risk rate of traffic accidents. According to the definition of full driving automation by the Society of Automotive Engineers [1], FADS can operate vehicles under all road conditions just as a skilled human driver and does not need any supervision from human drivers.

To widely realize the practical application of FADS in society, not only technical issues but also social and human issues should be solved, such as driver trust calibration. Based on Lee & See's definition of human-automation trust [2], driver trust in FADS is the attitude of the driver that driving automation will help achieve the driver's driving targets in uncertain and vulnerable driving situations. When the level of driver trust in FADS is comparable with the trustworthiness of driving automation itself, driver trust can be considered as an appropriate calibration of trust [3]. This means that driver trust matches the actual ability of driving automation. Otherwise, distrust or overtrust appears, when the level of driver trust is lower or higher than the trustworthiness of driving automation [2,4]. It has already been found that appropriate trust in FADS is essential because it has a positive relationship with the usage intention and the social acceptance of driving automation [5]. Instead, distrust can lead to disuse [6], and may therefore be a great obstacle to the social realization of driving automation [7,8]. Whereas, overtrust can lead to misuse [6], and cause fatal accidents. Although FADS can work under all road conditions, it will also face the problems of driver overtrust and misuse. Because there are still some unable driving situations of FADS such as glare ice, white-out snow storm, and flooded roads [1], and some emergencies where manual control recovery (MCR) is needed. Drivers'

performances in the emergency MCR of FADS have been examined in an empirical study, and the authors found that overtrust can lead to a long reaction time when drivers do not have enough experience with FADS. Therefore, appropriate calibration of driver trust in FADS should be noticed to ensure the appropriate use of FADS in the future.

Development of human trust in automation

Trust is dynamic because trust can develop as the relationship between the trustor and trustee develops [2,9]. According to the dynamic trust framework [10], trust in FADS develops successively via dispositional trust, initial trust, ongoing trust, and post-task trust. Dispositional trust can reflect a driver's propensity to trust [11,12], which depends on the driver’s culture, age, gender, and personality traits [12], and early trust-related experiences [13]. Since dispositional trust develops before the first interaction with driving automation, or even before the recognition of driving automation, it is not influenced by the characteristics of driving automation itself and driving contexts [12]. Initial trust refers to the trust developing from dispositional trust when the driver has just known about the driving automation but has not used it. Therefore, initial trust is influenced by both dispositional trust and drivers' indirect experiences related to driving automation [10], such as related information acquired from other people [2]. Unlike dispositional trust and initial trust, ongoing and post-task trust develop relying on direct contacts with driving automation. Ongoing trust is formed during the interaction, based on drivers' initial trust and perceived real-time characteristics of driving automation. Whereas, post-task trust is formed after completing the interaction with driving automation [10,11].

In the present study, we aim to focus on initial trust, because initial trust plays an important role in

the connection between dispositional trust, ongoing trust, and post-task trust [10,11], and can affect users' initial decision to engage in the automation [2]. In Merritt & Ilgen's study of operators' trust in Automatic Weapons Detector (AWD) [11], it has been found that operators' initial trust formed based on the first impression of the AWD has a positive relationship with operators' post-task trust measured immediately after the usage of AWD. That is, no matter whether the level of trust changed with the first experience of the automation, the higher the level of initial trust, the higher the level of post-task trust. Therefore, besides increasing the experiences of automation to adjust ongoing trust and post-task trust from the prior level of trust to a more appropriate level [14,15], adjusting the level of initial trust before the first experience may also be an effective way with less difficulty and cost. In addition, Merritt & Ilgen [11] have also found that initial trust is positively associated with operators' usage decisions on automation. According to this, it seems that excessive initial trust before experiencing the automation may lead to initial misuse, whereas inadequate initial trust before experiencing the automation may lead to initial disuse. Finally, vehicles adopted with FADS still do not yet come into the market. The public has known FADS, but still does not have any experience or even adequate understanding of FADS. Therefore, the development of public trust in FADS is now at the stage of *initial trust*.

Factors that influence the formation of the trust

According to Lee and See's conceptual model of the dynamic process of trust in automation [2], prior information about automation can form the basis of initial trust before interaction. On the one hand, analytic information helps trustors to develop trust in a cognitively demanding process with

rational assessments about whether the automation is trustworthy. Such as reliability information of the automation [16], and analytic information provides trustors with direct evidence about the trustworthiness of automation. On the other hand, analogical information helps trustors to develop trust in a less cognitively demanding process of assessing category membership. Such as observed interface features of the automation, acquired gossip related to the automation, and perceived brand reputation of the brand that developed the automation, analogical information governs the formation of trust in an indirect way. Many empirical studies have verified the influence of analytic information on trust formation [14,17]. For example, compared with low-reliability information, high-reliability information can lead to a higher level of Trust in FADS [16]. However, for analogical information, there was a lack of empirical evidence. Although Forster et al. [16] have focused on brand reputation, they did not find any effect of brand reputation on trust.

A brand is a name, symbol, trademark, and design to be used as the unique identity of an individual, organization, or company in commodity and services owned to differentiate from those of its competitors [18,19]. Just as a person has personality, a brand similarly has a brand personality (the set of human characteristics associated with a brand), including sincerity (down-to-earth, honest, wholesome, and cheerful), excitement (daring, spirited, imaginative, and up-to-date), competence (reliable, intelligent, and successful), sophistication (upper class and charming) and ruggedness (outdoorsy and tough) [20]. According to Celmer, Branaghan, & Chiou's framework for trust in branded autonomous vehicles [21], drivers' perceived brand personality will influence drivers' trust in and performance expectations for autonomous vehicles. In other words, before making a judgment of the actual trustworthiness of an automated driving system (ADS), drivers may make a prior judgment

about the identity and the brand personality of the automobile brand that developed the ADS. Therefore, analogous information from a brand that can influence the formation of initial trust in FADS should not be limited to brand reputation. Instead, the brand should be considered as a whole that can convey kinds of information about each aspect of the brand personality. However, empirical evidence is needed to verify the effect of automobile brands or perceived kinds of brand personality of automobile brands on drivers' trust in FADS, which is one of the aims of this study.

Besides the automobile brand itself, drivers' trust in the automobile brand that developed the driving automation may be another possible influence factor in the formation of initial trust in driving automation. Plenty of previous studies in terms of consumer goods have verified that consumers' purchase intention to a large extent depends on consumers' trust in the brand [22-24]. For consumer goods, for example, in the online shopping context, it was confirmed that website brands would influence consumers' trust and perceived risk towards the online retailers, and thus influence consumers' purchase intention in turn [25]. Likewise, equipped with driving automation or not, the automobile is originally one kind of consumer good - referred to be products purchased by consumers for their enjoyment and improvement of living standards, including automobiles, houses, food, clothing, and others [26]. It is thus easily found when consumers plan to purchase a new car equipped with ADS, they would consider whether it was developed by a trustworthy automobile brand. However, there is less direct evidence indicating the relationship between trust in automobile brands and trust in ADS products. Therefore, it needs to examine the existence and the extent of the impact of trust in automobile brands on trust in ADS.

Cognition of Automobile Brand

If drivers' trust in ADS depends on automobile brands that develop the ADS and can be influenced by drivers' trust in the automobile brands, it is necessary to discuss what kinds of automobile brands can win more trust in the brand itself and the ADS. Previous studies have found that consumers' trust in one brand depends on various factors, such as consumer satisfaction [27], perceived product quality of the brand [27,28], business behavior and business climate of employees and organization [27,29-31], and corporate credibility [32]. Besides, according to the salient value similarity (SVS) model proposed by Earle and Cvetkovich [33], people would trust in and entrust a person or an organization who possessed the same or similar viewpoints, feelings, and cognition of values. On these grounds, lots of factors that influence brand trust should be related to people’s prior knowledge and information in people’s cognition of the brand. In a study of interpersonal trust among managers and professionals in organizations, McAllister [34] addressed the nature of interpersonal trust - the extent of affect-based trust and the extent of cognition-based trust. The level of cognition-based trust is higher than affect-based trust and is to some extent necessary for the development of affect-based trust. Here, cognition-based trust refers to making a choice on whom we will trust based on "good reasons" constituting evidence of trustworthiness according to prior knowledge and information in our cognition [35]. Similarly, human-computer trust concludes both cognitive and affective components as well, although affective components became the stronger indicator of trust [36]. Accordingly, people’s cognition of a trustee plays an important role in people’s trust in the trustee of no matter person, organization, or machine. Furthermore, due to different experiences, people can have different cognitions of the same brand. To better understand the relationships among driver trust in FADS, driver trust in automobile brands, and automobile brands, clarifying drivers' cognition bases of the

automobile brands that develop the FADS is necessary.

Purpose of This Study and Hypotheses

As outlined, automobile brand and drivers’ trust in automobile brand have the potential to influence drivers’ initial trust in FADS. However, few empirical studies have provided direct evidence to verify whether and to what degree the influences exist.

In this study, we aim to test whether drivers' initial trust in FADS depends on automobile brands that develop the FADS as well as drivers’ trust in the automobile brands, and explore what kinds of automobile brands can win much more initial trust in FADS by clarifying drivers' cognitive structures of the automobile brands. To achieve these, we conduct two online surveys. The followings are our hypotheses.

Hypothesis 1 (H1): Drivers’ trust in automobile brands positively impacts drivers' initial trust in FADS.

Hypothesis 2 (H2): The levels of drivers' initial trust in FADS were different across different automobile brands.

Hypothesis 3 (H3): Drivers' cognitive structures of automobile brands with higher and less initial trust in FADS have distinctly different characteristics.

Survey 1

Methods

Procedure and participants

An online survey was conducted by commissioning an internet research company in June 2020 and 206 anonymous Japanese drivers (103 males and 103 females) aged 18-69 years (M = 44.6 years, SD = 11.8) with an ordinary license living in Japan took part in the survey (from June 8th to 9th, 2020).

In the survey, participants were asked to finish 9 questionnaires about 9 randomly chosen automobile brands that are developing driving automation. The 9 questionnaires were presented to participants in random orders to counterbalance the order of automobile brands. Besides, due to the ethical request according to the ethical review board of the Faculty of Engineering, Information, and Systems at the University of Tsukuba, the actual names of brands were used in questionnaires for data collecting but were hidden under the code name like Brand 1 in the current paper. Brand 1, Brand 2, and Brand 3 were famous automobile brands from Japan. Brand 4 was a famous automobile brand from the USA. Brand 5, Brand 6, Brand 7, and Brand 8 were famous automobile brands from Germany. Brand 9 was a famous automobile brand from Sweden. For each automobile brand, the questionnaire had two sections. The first section was a "brand trust scale" to measure drivers' trust in automobile brands. The second section was a "human-automation trust scale" to measure drivers' initial trust in the FADS developed by automobile brands, after instructing participants to imagine that the automobile brand would sell cars with FADS. Before starting all the questionnaires, necessary explanations for FADS were given to the participants following [1].

This survey was approved by the ethical review board of the Faculty of Engineering, Information,

and Systems at the University of Tsukuba (Approval number 2020R369-1). All participants were paid following the regulations of the internet research company.

Scales

The human-automation trust scale used in the current study was proposed by Muir and Moray [9], including four items of predictability, dependability, faith, and overall trust (Table 1). The brand trust scale used in the current study was the 8 items of the first factor "trust" of the "Japanese consumer-brand relationship scale" developed by Hatai [37] (Table 1). Both the two scales were used with a 7-point Likert scale (0: I don't think so, 6: I think so).

Table 1. Items in trust questionnaires [9,37].

Scale	Item
**Trust in brand**	TB1 I trust this brand.
	TB2 I can be in sympathy with the products and services of this brand.
	TB3 If the products come from this brand, I will be relieved.
	TB4 If someone asks about this brand, I will recommend it.
	TB5 I have a good image for this brand.
	TB6 There is no failure of this brand.
	TB7 This brand won't let me down.
	TB8 The quality of this brand's products and services is excellent.
**Trust in FADS**	TA1 To what extent can the behavior of the FADS be predicted from moment to moment? (Predictability)
	TA2 To what extent can you count on the FADS to do its job? (Dependability)
	TA3 To what extent will the FADS be able to cope with other system states in the future? (Faith)

	TA4 To what extent do you trust the FADS? (Overall trust)

Data analysis

To verify H1, structural equation modeling (SEM) was conducted in Amos 26.0. Before the analysis of the structural model to examine the impact of trust in automobile brand on trust in FADS, it is necessary to examine the goodness of fit (GOF) of the measurement model. Therefore, confirmatory factor analysis (CFA) on the two variables - trust in automobile brand and trust in FADS was performed first, and the two measurement models were then modified with modification indices [38] until the moderate GOF was obtained. After checking the reliability and validity of the two modified measurement models, structural model analysis was conducted, during which overall GOF was evaluated by recommended model fit indices. Commonly used fit indices include Chi-square/df, the goodness of fit index (GFI), adjusted goodness of fit index (AGFI), comparative fit index (CFI), and root mean square error of approximation (RMSEA). Chi-square/df should be less than 5 (3 if strictly), GFI, AGFI, and CFI should be greater than 0.9, and RMSEA should be less than 0.5 [39].

At last, to verify H2, a repeated measures ANOVA was employed in SPSS 26.0 to analyze the main effects of automobile brand on both trust in automobile brand and trust in FADS.

Results

CFA and evaluation of reliability and validity

The model fit indices of both the two original measurement models did not show moderate fit to data, trust in automobile brand：$\chi^2_{(20)}$ = 388.80, *GFI* = .949, *AGFI* = .908, *CFI* = .979, *RMSEA* = .100; trust in FADS：$\chi^2_{(2)}$ = 74.60, *GFI* = .979, *AGFI* = .896, *CFI* = .990, *RMSEA* = .140. Therefore, for trust in automobile brand, item TB6, TB8, TB5 and TB2 were then removed during

four rounds of modification successively so that moderate GOF was obtained, $\chi^2_{(2)}$ = 3.19, *GFI* = .999, *AGFI* = .996, *CFI* =1.00, *RMSEA* = .018. For trust in FADS, item TA1 (Predictability) was removed, and thus moderate GOF was obtained, $\chi^2_{(0)}$ = .000, *GFI* = 1.000.

Modified models showed moderate internal consistency of each factor because Composite Reliability (CR) values were greater than 0.6 (Table 2). Besides, all of the factor loads of items were greater than 0.5 and significant (p < .01); Average of Variance Extracted (AVE) values were greater than 0.5; Square Multiple Correlations (SMC) values were greater than 0.5 (Table 2). These met the evaluation criteria set up by Hair et al. [39], and thus reflected the good convergence validity of modified measurement models of trust in automobile brand and trust in FADS. Then a good discriminant validity was also considered because the square root of AVE for each factor was larger than the correlation coefficient between trust in automobile brand and trust in FADS (Table 3) [40].

Table 2. Reliability and convergence validity.

		**Parameter significance estimation**				**Convergence validity**			
		**United.**	**S.E.**	**T-value**	**P**	**Std. Estimate**	**SMC**	**C.R.**	**AVE**
**Trust in automobile brand**	**TB1**	1				.886	.785	.938	.791
	**TB3**	1.093	0.017	63.81	***	.944	.891		
	**TB4**	0.947	0.02	47.388	***	.819	.671		
	**TB7**	1.014	0.017	58.29	***	.904	.817		
**Trust in FADS**	**TA2**	1				.916	.839	.943	.846
	**TA3**	1.01	0.016	64.094	***	.913	.834		
	**TA4**	1.045	0.016	66.831	***	.930	.865		

Table 3. Discriminant validity.

	AVE	Trust in FADS	Trust in automobile brand
**Trust in FADS**	.791	.889	
**Trust in automobile brand**	.846	.652	.920

The numbers in bold on the diagonal are the square roots of AVE on the left.

0.652 is the correlation coefficient between trust in automobile brand and Trust in FADS.

SEM of driver trust

Fig 1 shows the final SEM of driver trust with standardized path coefficient, as well as the reported fit indices values. In the measurement model, faith, dependability, and overall trust are all significant factors positively influencing trust in FADS (factor load = 0.91, 0.92, and 0.93, *ps* < .001). In the structural model, trust in automobile brand positively influences trust in FADS (Std. Estimate = 0.652, *p* < .001). As for model fit, the value of Chi-square/df is around 5, the values of GFI, AGFI, and CFI are greater than 0.9, and the value of RMSEA is less than 0.05. These results indicate an acceptable model fit [39].

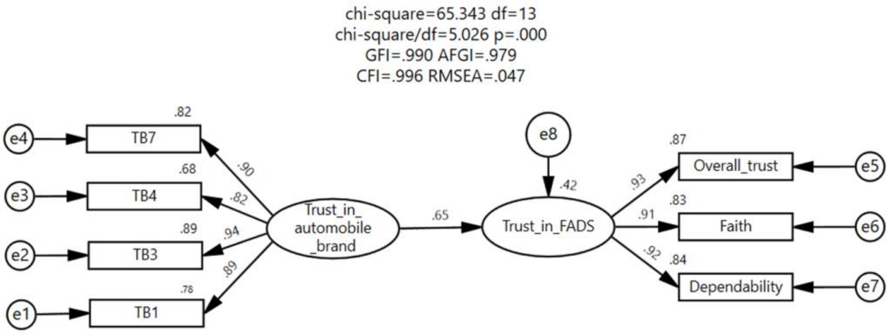

Fig 1. SEM of driver trust.

Differences in trust among automobile brands

The average scores of trust in automobile brand and trust in FADS for the nine automobile brands were plotted in Fig 2.

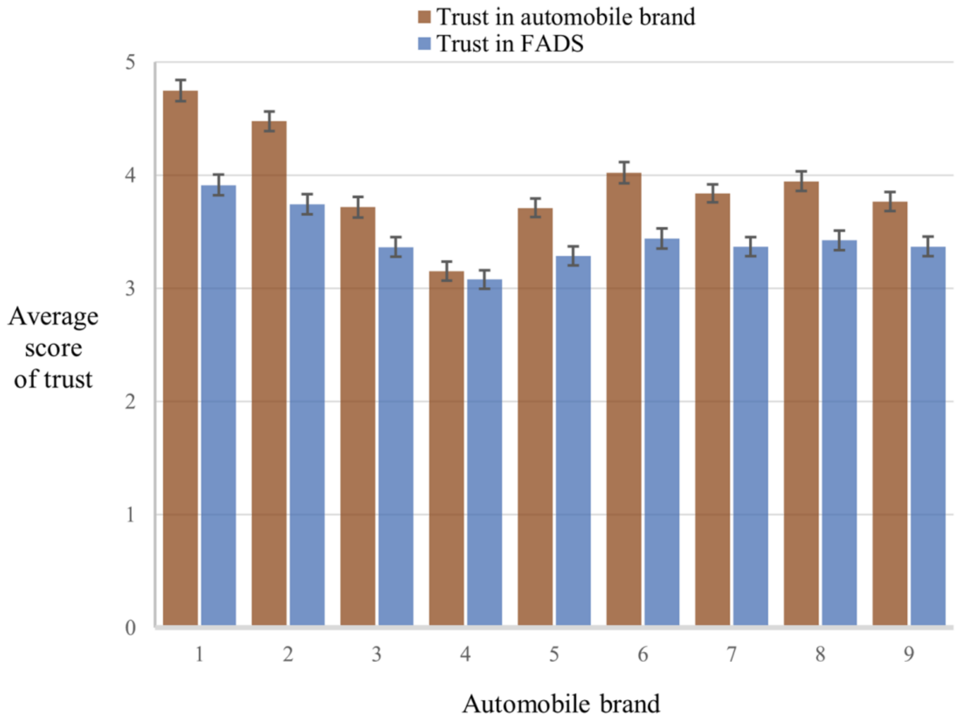

Fig 2. Average scores of trust in automobile brand and trust in FADS for nine automobile brands. Error bars are standard errors of means.

The results of a repeated measures ANOVA (after Greenhouse-Geisser correction) showed that the main effect of automobile brand was significant on both trust in automobile brand ($F_{(5.75,\ 1179.37)}$ = 53.17, p < .001, *partial* η^2 = 0.206) and trust in FADS ($F_{(6.21,\ 1272.96)}$ = 22.74, p < .001, *partial* η^2 = 0.100).

Post-hoc pairwise comparisons corrected by the Bonferroni method showed that 1) for both trust in automobile brand and trust in FADS, the average scores for Brand 1 and Brand 2 had no significant difference (p = .075; p = .538), but were significantly higher than the average scores for the other

seven brands (Brand 3-9) (*ps* $< .05$); 2) the average scores of both trust in automobile brand and trust in FADS for Brand 4 were significantly lower than the ones for almost all of the other eight brands (Brand 1-3, 5-9) (*ps* $< .01$), except from the comparable result with Brand 5 ($p = .225$) for trust in FADS; 3) among the average scores of both trust in automobile brand and trust in FADS for the rest of brands (Brand 3, 5-9), there was almost no significant difference (*ps* $> .05$), except from the significant differences between Brand 5 and 6 ($p = .000$), Brand 5 and 8 ($p = .015$), and Brand 6 and 9 ($p = .018$) for trust in automobile brand.

Survey 2

The above results of repeated measures ANOVA showed there were significant differences in trust in automobile brand and trust in FADS among different brands. Then based on the results of pairwise comparisons, we could divide Brand 1 and Brand 2 into a high trust group, divide Brand 4 into a low trust group, and divide the remaining six brands (Brand 3, 5-9) into a medium trust group. However, it is still not known why some brands were highly trusted and some were not. Consequently, survey 2 was conducted to get some cues from drivers' cognitive structures of those automobile brands.

Methods

Participants

An online survey was conducted by commissioning an internet research company in January 2021, and anonymous responses were received (from January 8th to 9th, 2021) from 103 Japanese drivers (58 males) aged 18-70 years (M = 50.7 years, SD = 12.5) with a normal license living in Japan. The

participants were paid following the regulations of the research company. This survey was approved by the ethical review board at the University of Tsukuba (Approval number 2020R369-1).

Data collection

In this survey, we chose Brand 1 and 2 in the high trust group, Brand 3 and 5 in the medium trust group, and Brand 4 in the low trust group from survey 1 as the research objects. We used Free Word Association Test (FWAT) as the data collection tool, which is one of the widely used methods for revealing and analyzing the cognitive structure of somebody or something [41-45]. It could reveal the type and number of concepts retrieved from long-term memory and the links among them [46].

The name of the above five brands has been presented one by one to participants as stimulus words. According to the previous application of FWAT, participants might write down the next word associated with the previous response words that they have written down rather than the stimulus word itself [41-44,46], which was called the chain reaction. To avoid the risk of chain reaction, each stimulus word (name of the brand) was written 5 times on one page just as the following example (Fig 3). Participants were asked to write down the first 5 words associated with a stimulus brand for an unlimited duration when each stimulant brand came out. The same as survey 1, the actual name of each brand was used to collect research data but was hidden under the code names like Brand 1 in the current paper.

Stimulus Concept: Brand 1
Brand 1: ______________
Brand 1: ______________
Brand 1: ______________
Brand 1: ______________
Brand 1: ______________

Fig 3. Example of FWAT data collecting.

Data analysis

To verify H3, we analyzed FWAT data based on the content analysis method that has been widely used in previous studies with high reliability [41-44,46]. Combined with the purpose of the current study, there were 6 analysis processes. 1) Count the valid word number for each brand, which refers to the total number of words written down by participants after excluding invalid words like "none" or "unknown"; 2) Calculate the effective rates of the valid words using the [valid word number / planned word number (5) * participant number (103)] formula; 3) Calculate different word number for each brand. Different word number is the number of words different from the others among valid words. Table 4 illustrated the difference between valid word number and different word number with an example; 4) Analyze the correlation between the level of trust and valid word number as well as different word number; 5) Categorize words with the same meaning or property after excluding words stated for only one time and not associated with other words. This process was checked one more time and some words were merged with other similar words or shifted to other categories; 6) Calculate the word number of each category and draw drivers' cognitive structures for each brand.

Table 4. An example for different word number and valid word number.

Category	Different word	Valid word number
**Brand personality**	1. First-class	3
	2. Number one in Japan	3
	3. Number one in the world	5
**The sum**	3	11

This is an example taken from a part of the data. For the category "Brand personality," there were 3 different words obtained (the medium column), the different word number thus was 3. The three different words were stated repeatedly for 3, 3, and 5 times respectively, thus the valid word number for the three different words were 3, 3, and 5, and the total valid word number for this category was 11.

To ensure the reliability of word categorizing, it was checked whether each word belonged to the current category by two other researchers in our department. The consistency between the checking results for each word given by the two researchers was marked as "Consensus" or "Divergence," and the reliability was calculated by the [Consensus / (Consensus + Divergence) X 100] formula [47]. Because no word was marked "consensus" that the word did not belong to the current category, no word was moved to other categories in this process. In the end, the intercourse reliability was 92%, which could be seen as reliable [48].

Results

Valid word number, different word number, and trust

As some participants were unable to come up with all five words related to some automobile brands, 540 (effective rate 20.97%) valid words were obtained for all automobile brands, among which there were 338 different words (Table 5). Both valid word number and different word number for high trust group (M = 142.5, M = 20.5) were more than medium trust group (M = 103.5, M = 13.5), and low trust group (M = 48, M = 6).

Table 5. Valid word number, different word number, and trust.

**Trust**	**Brand**	**Valid word numbers**	**Effective rate**	**Different word number**	**Trust in brand**	**Trust in FADS**
**High**	**1**	163	31.65%	106	4.75	3.91
	**2**	122	23.69%	77	4.48	3.74
**Medium**	**3**	132	25.63%	76	3.72	3.36
	**5**	75	14.56%	46	3.70	3.28
**Low**	**4**	49	9.51%	33	3.15	3.08
**Sum**		540	20.97%	338	/	/

To confirm the relationship between valid word number, different word number, and trust in automobile brand as well as trust in FADS, a correlation analysis was conducted. Table 6 showed 1) a significantly positive correlation between valid word number and different word number; 2) positive correlations between valid word number and both trust in automobile brand and trust in FADS, although the correlations were non-significant; 3) significantly positive correlations between different word number and both trust in automobile brand as well as trust in FADS. However, Brand 3 was an exception. Although valid word number and different word number were between the two brands in

the high trust group, it belonged to the medium trust group, in which both the levels of trust in automobile brand and trust in FADS were lower than brands in high trust group.

Table 6. The correlation coefficient of word number and trust.

	**Count of category**	**Valid word number**	**Different word number**	**Trust in automobile brand**
**Count of category**				
**Valid word number**	.647 (*sig* = .238)			
**Different word number**	.732 (*sig* = .159)	.989**		
**Trust in automobile brand**	.829 (*sig* = .082)	.857 (*sig* = .064)	.899*	
**Trust in FADS**	.825 (*sig* = .082)	.874 (*sig* = .053)	.920*	.995**

** The correlation was significant at 0.01 level; * The correlation was significant at 0.05 level.

Cognitive structure by categories

For each automobile brand, words listed only once were deleted, and the remaining words (338 words) were categorized according to the meaning or the property. Fig 4 showed the categories and the word number of each category for each automobile brand.

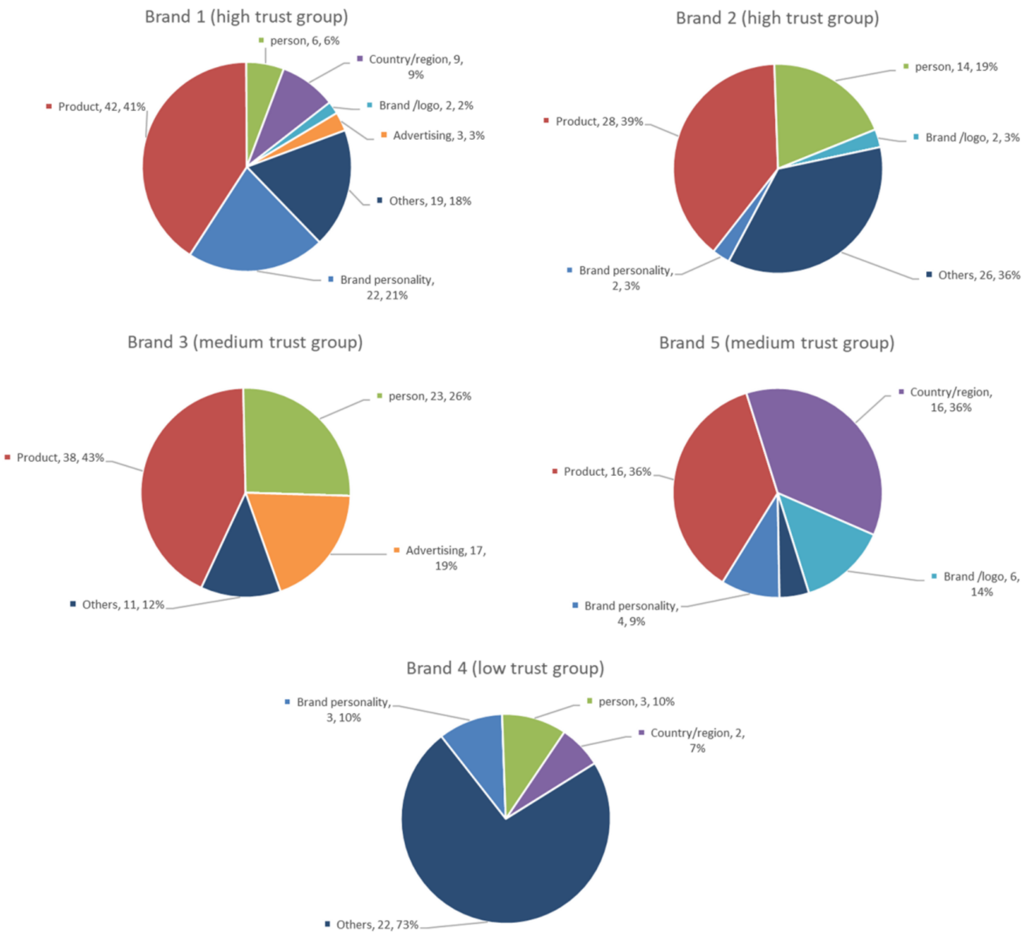

Fig 4. Categories and the word number of each category for each automobile brand.

On the whole, the remaining words were divided into 7 categories, "brand personality," "product," "person," "country/region," "brand/logo," "advertising" and "others." The category of "brand personality," contained words that described participants' perceived brand personality, such as "reliable" and "safe." The category of "product" contained words of the name of specific products. The category of "person" contained names of persons related to the brand, such as the CEO or the advertising spokesperson. Then in the category "country/region," there were names of countries or cities the brands were located in. In the category of "brand/logo," words related to the brand or the logo itself were associated, such as aliases or what the logo looked like. Finally, words from the advertising slogans were contained in the category "advertising," and words that do not belong to any

one of the above six categories were contained in the category "others." To clearly understand the drivers' cognitive structure of each brand, we drew word clouds with the remaining words (Figs 5-9). However, due to ethical reasons, we hid some words such as the name of the product that may easily lead readers to know what these brands are with mosaic. In Figs 5-9, black words in the periphery are the names of the categories, and words in different colors belong to different categories. For example, red words are the names of specific products and green words are the name of a person. For these words in the word clouds, the more frequently the word was associated, the larger the font size of the word.

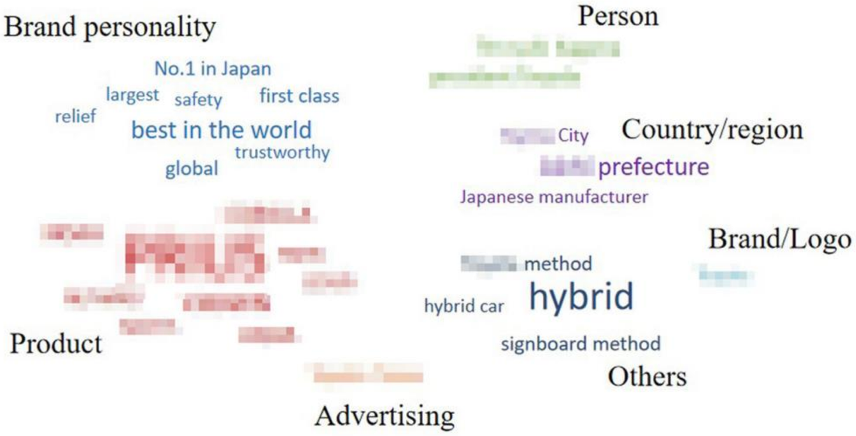

Fig 5. Drivers' cognitive structure of Brand 1 in high trust group.

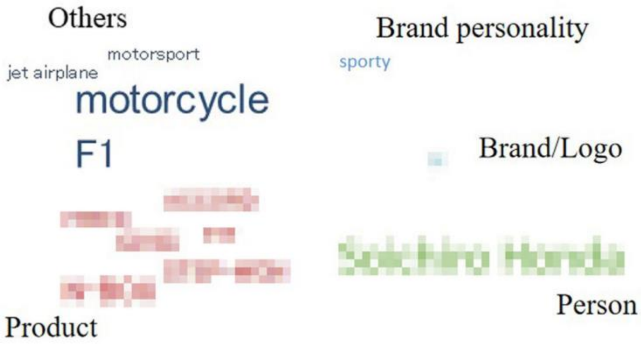

Fig 6. Drivers' cognitive structure of Brand 2 in high trust group.

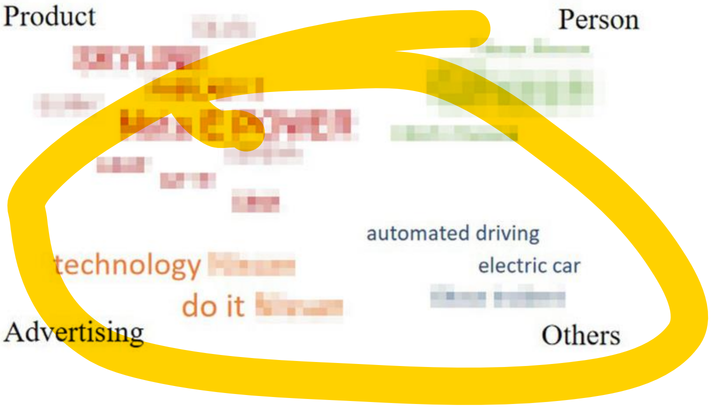

Fig 7. Drivers' cognitive structure of Brand 3 in medium trust group.

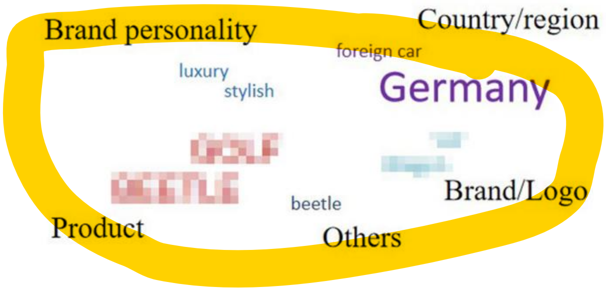

Fig 8. Drivers' cognitive structure of Brand 5 in medium trust group.

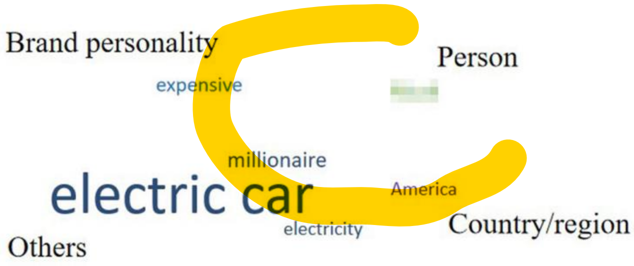

Fig 9. Drivers' cognitive structure of Brand 4 in low trust group.

Importantly, the count of the category was more for brands in the high trust group (M = 6) than brands in the medium (M = 4.5) and low trust group (M = 4) and was highly positively correlated with both trusts in automobile brand and trust in FADS, although it was non-significant for both trust in automobile brand and trust in FADS (Table 6). Moreover, there was some universality and individuality in categorizing the associated words among brands. 1) For brands in the medium and

high trust group, about 40% of words relevant to "product" were associated with them. Instead, for the brand in the low trust group (Brand 4), there was no associated word relevant to "product"; 2) For all brands in the medium and high trust group, "others" were just a small part (the biggest proportion was 36% for Brand 2) or even did not exist. Whereas, the proportion of “others” reached up to 73% for Brand 4 in low trust group; 3) For Brand 3 in the medium trust group, there was no part of "brand personality," while there was for the other four brands. 4) For Brand 5 in the medium trust group, there was no part of "person," while there was for the other four brands.

Words in cognitive structures related to unethical events

After analyzing the numbers of associated words and drivers' cognitive structures, the content of each associated word was checked one by one so that any special cognition in drivers' cognitive structures would not be missed. Finally, it was found that for Brand 3 in the medium trust group, there were some associated words relevant to unethical events that happened in recent years (23.6% of total associated words). Instead, there was no negative word associated with the other 4 brands.

Discussions

The current study explored the relationship among automobile brands, drivers’ trust in automobile brand and drivers’ initial trust in FADS, and clarified the common characteristics of drivers' cognitive structures about automobile brands that can win higher trust in automobile brand and higher initial trust in FADS. As the hypothesis, drivers' initial trust in FADS was different across automobile brands and was positively impacted by drivers' trust in the automobile brands. Moreover, drivers' cognitive structures about automobile brands in the high trust group have some common characteristics. This section discusses these findings and integrate them with previous studies to provide implications for the practice of FADS.

The impact of trust in automobile brand on initial trust in FADS

Consistent with H1, we found that when drivers do not have any direct interaction experiences with FADS, their initial trust in FADS can be directly affected by the level of their trust in the automobile brand that developed the FADS. Specifically, according to the results of survey 1, there was a positive impact from trust in automobile brand on trust in FADS. Accordingly, for automobile brands, promoting drivers’ trust in automobile brands themselves may also be a possible approach to promote drivers’ initial trust in their FADS. Besides, the positive impact of trust in automobile brand on trust in FADS also means that overtrust in automobile brand can lead to overtrust in FADS, whereas distrust in automobile brand can lead to distrust in FADS. Previous studies have already found the effectiveness of elaborate practice and adequate knowledge of driving automation on the calibration of drivers’ initial trust in driving automation [15,49]. However, before calibrating drivers’ initial trust

in driving automation, drivers' prior level of trust in the automobile brand should also be considered as a precondition.

Effect of automobile brand on initial trust in FADS

Since significant differences in the level of consumers' brand trust are existing among brands, we examined the discrimination of the chosen 9 automobile brands in the level of drivers' brand trust before verifying H2 to avoid the type I error during the verification of H2. The results showed that drivers' trust in automobile brand was indeed different across different automobile brands, which indicated the good discrimination of the chosen automobile brands. Then according to the subsequent results, we found that drivers' initial trust in FADS was also different across different automobile brands, and H2 was thus verified. This means that automobile brand can certainly influence drivers' initial trust in FADS, which provided empirical evidence for Lee and See's conceptual model of the dynamic process of trust in automation [2] and Celmer et al.'s framework of trust in branded driving automation [21]. To adjust drivers' initial trust in FADS, drivers' prior identification and judgment of the automobile brand that developed the FADS should be considered. For driving automation that is developed by different automobile brands, different trust calibration strategies and approaches are thus necessary.

Drivers' cognitive structure of automobile brand

Based on the analyses of the associated words from automobile brands via FWAT, H3 was confirmed and several common characteristics of drivers' cognitive structures of the automobile

brands that can win higher trust in automobile brand and higher initial trust in FADS could be concluded.

Characteristic 1: rich and various.

The results showed that for automobile brands in the high trust group, both the number and the category of associated words were far more than the medium and low trust groups. Therefore, it is considered that for automobile brands that can win higher trust in automobile brand and higher initial trust in FADS, drivers have richer and more varied cognitive structures. This means that to acquire higher trust in both the automobile brand itself and the FADS, automobile brands should be well-known and familiar to drivers in every aspect, which is related to the accumulation of the achievement of long-term brand promotion. Generally, familiarity is regarded as a precondition to trust in somebody or something [50], and it has been widely verified in different fields according to existing studies [51,52]. For example, Ha and Perks [53] found that a high level of brand familiarity among consumers was one of the dimensions of brand trust achievement.

Characteristic 2: Have about 40% of the cognition of the product.

According to the results, no word relevant to “product” was associated with Brand 4 in the low trust group, instead of which for the brands in the high and medium trust groups plenty of automobile products were associated. Even though the word number of "product" was quite different for each brand, the proportions of "product" on the total word number of each brand were almost 40%. Among these associated products, many are classic automobile products with decades of history as well as generations of innovation, and some are special automobile products with special functions or techniques. This indicates that quality assurance of products plays an important role in brand

development. As it has been found, product quality can influence the level of brand trust by helping to create consumers' satisfaction [27], and finally influence customers' loyalty to the brand [28].

Characteristic 3: Have some cognitions more or less related to brand personality or related person.

The results also showed that no word relevant to "person" or “brand personality” was associated with brands in the medium trust group. It could thus be considered that for brands with medium trust, although the part of "product" existed in drivers' cognitive structures to almost the same degree as brands with high trust, there was always a missing piece such as "person" or “brand personality." Accordingly, on the one hand, using the public influence of brand spokespersons to help promote brand influence and brand trust seems effective. Although it was found that the attractiveness of an advertisement spokesperson seems relatively unimportant when consumers assess a new high-technology product [54], a better fit between spokesperson attribute and product attribute could help to better shape consumers' attitudes toward the brand [55]. On the other hand, for automobile brands that can win higher trust in brand and higher initial trust in the products, drivers might have more cognitions of brand personality, like first-class, the best in the world, trustworthy, safe, and global. This is accordant with the previous study that pointed out the relationship between brand personality and brand trust [56] and provided empirical evidence for Celmer et al.'s framework of trust in branded driving automation [21].

Characteristic 4: Have no negative cognition about the unethical event in drivers' cognitive structures.

Focusing on Brand 3, it could be easily found that the valid word number and different word

number of Brand 3 were both between the two brands in the high trust group - Brand 1 and Brand 2, which means that drivers' cognitive structures for Brand 3 were almost as rich and various as Brand 1 and Brand 2 so that Brand 3 should also be highly trusted. However, it is contradictory that Brand 3 was finally in the medium trust group. Because Brand 3 was the only brand, for which there were some associated words (23.6% of total associated words) relevant to unethical events, it is concluded that the negative cognition composed by these words might be invalid in drivers' cognitive structures to help build trust or could even cut back the trust built before. According to previous studies of business behavior and consumer trust, unethical business behavior and the business climate of employees and organizations are significantly related to a lower level of trust [29,30]. On the contrary, ethical business behavior could positively influence consumers' trust [27,31]. Consequently, the negative part about unethical events in cognitive structure might lower drivers' trust in automobile brands themselves and their products, and therefore it might be necessary for brands to avoid the forming of drivers' negative cognition about such as unethical events.

Practice implications

As outlined, findings in the current research have several practice implications for calibrating initial trust in FADS as well as ADS of other levels. To avoid the disuse and misuse of ADS, the influences from automobile brands should be considered during the initial trust calibration of ADS. For automobile corporations, figuring out consumers' cognitions of their automobile brands and carrying out targeted brand management based on consumers' cognitions are needed. This may help eliminate consumers' distrust in their ADS. On the contrary, drivers' overtrust in ADS of some highly trusted

automobile brands may be existing, which can lead to the misuse of ADS and thus cause negative consequences such as severe accidents. Therefore, for a more appropriate initial trust of drivers in ADS, it is also necessary to pay attention to the prevention of drivers' overtrust in the automobile brands of the ADS

Limitations

Firstly, this study selected only nine automobile brands to verify our hypotheses in survey 1, and only five brands were selected from the nine automobile brands to continue survey 2. However, besides the selected brands, there are many other brands or companies developing driving automation. The relationships between automobile brands, trust in automobile brand, initial trust in FADS, and drivers' cognitive structures of automobile brands, therefore, need to be examined with more automobile brands. On the other hand, because only one or two brands were selected for each trust group in survey 2, much more conclusions about drivers' cognitive structures of automobile brands cannot be drawn. Moreover, all of the findings in the current study should be further confirmed with experimental research that can manipulate brand characteristics to observe the differences in the levels of initial trust in driving automation.

Conclusion

In conclusion, the level of drivers' initial trust in FADS depends on automobile brand that developed the FADS and can be positively influenced by the levels of drivers' trust in the automobile brands. For automobile brands that can win higher levels of drivers' trust in the automobile brand and

drivers' initial trust in FADS, drivers possess more rich and various cognitive structures with some particular characteristics. Consequently, influence from automobile brands and drivers' prior trust in the automobile brands should be noticed for initial trust calibration of FADS to avoid the disuse and misuse of FADS in the future.

Funding

This work was supported by the JSPS KAKENHI [grant number 17KT0153] awarded to M.I.

References

1. SAE. Taxonomy and definitions for terms related to on-road motor vehicle automated Driving Systems. 2018;J3016_201806. Available from: https://www.sae.org/standards/content/j3016_201806/
2. Lee JD, See KA. Trust in automation: Designing for appropriate reliance. Human Factors: The Journal of the Human Factors and Ergonomics Society. 2004;46(1): 50-80. doi: 10.1518/hfes.46.1.50_30392 PMID: 15151155
3. Lee JD, Moray N. Trust, self-confidence, and operators' adaptation to automation. International Journal of Human-Computer Studies. 1994;40: 153-184. doi: https://doi.org/10.1006/ijhc.1994.1007
4. de Visser EJ, Cohen M, Freedy A, Parasuraman R. A design methodology for trust cue calibration in cognitive agents. In: International Conference on Virtual, Augmented and Mixed Reality. 2014; 251-262. doi: https://doi.org/10.1007/978-3-319-07458-0_24

5. Adnan N, Nordin SM, bin Bahruddin MA, Ali M. How trust can drive forward the user acceptance to the technology? In-vehicle technology for autonomous vehicle. Transportation Research Part A: Policy and Practice. 2018;118: 819-836. doi: https://doi.org/10.1016/j.tra.2018.10.019
6. Parasuraman R, Riley V. Humans and automation: Use, misuse, disuse, abuse. Human Factors: The Journal of the Human Factors and Ergonomics Society. 1997;39(2): 230-253. doi: https://doi.org/10.1518/001872097778543886
7. Hutson M. A matter of trust. Science. 2017;358(6369): 1375-1377. doi: 10.1126/science.358.6369.1375
8. Nastjuk I, Herrenkind B, Marrone M, Brendel AB, Kolbe LM. What drives the acceptance of autonomous driving? An investigation of acceptance factors from an end-user's perspective. Technological Forecasting and Social Change. 2020;161: 120319. doi: https://doi.org/10.1016/j.techfore.2020.120319
9. Muir BM, Moray N. Trust in automation: Part [illegible]. Experimental studies of trust and human intervention in a process control simulation. Ergonomics. 1996;39(2): 429-460. doi: 10.1080/00140139608964474 PMID: 8849495
10. Gao Z, Li W, Liang J, Pan H, Xu W, Shen M. Trust in automated vehicles. Advances in Psychological Science. 2021;29(12): 2172-2183. doi: 10.3724/SP.J.1042.2021.02172
11. Merritt SM, Ilgen DR. Not all trust is created equal: Dispositional and history-based trust in human-automation interactions. Human Factors: The Journal of the Human Factors and Ergonomics Society. 2008;50(2): 194-210. doi: 10.1518/001872008X288574 PMID: 18516832

12. Hoff KA, Bashir M. Trust in automation: Integrating empirical evidence on factors that influence trust. Human Factors: The Journal of the Human Factors and Ergonomics Society. 2015;57(3): 407-434. doi: 10.1177/0018720814547570 PMID: 25875432
13. Hardin R. Trustworthiness. Ethics. 1996;107: 26-42.
14. Hergeth S, Lorenz L, Krems JF. Prior familiarization with takeover requests affects drivers' takeover performance and automation trust. Human Factors: The Journal of the Human Factors and Ergonomics Society. 2017;59(3): 457-470. doi: 10.1177/0018720816678714 PMID: 27923886
15. Payre W, Cestac J, Delhomme P. Fully automated driving impact of trust and practice on manual control recovery. Human Factors: The Journal of the Human Factors and Ergonomics Society. 2016;58(2): 229-241. Doi: 10.1177/0018720815612319 PMID: 26646299
16. Forster Y, Kraus JM, Feinauer S, Baumann M. Calibration of Trust Expectancies in Conditionally Automated Driving by Brand, Reliability Information and Introductionary Videos: An online study. In: Proceedings of the 10th International Conference on Automotive User Interfaces and Interactive Vehicular Applications – Automotive UI '18. 2018;118-128. doi: https://doi.org/10.1145/3239060.3239070
17. Korber M, Baseler E, Bengler K. Introduction matters: Manipulating trust in automation and reliance in automated driving. Applied Ergonomics. 2018;66: 18-31. doi: 10.1016/j.apergo.2017.07.006 PMID: 28958427
18. Aaker DA. Managing brand equity: capitalizing on the value of a brand name. New York: The Free Press; 1991.

19. Cretu AE, Brodie RJ. The influence of brand image and company reputation where manufacturers market to small firms: A customer value perspective. Industrial Marketing Management. 2007;36(2): 230-240. doi: https://doi.org/10.1016/j.indmarman.2005.08.013
20. Aaker JL. Dimensions of Brand Personality. Journal of Marketing Research. 1997;34(2): 347–356. doi: https://doi.org/10.2307/3151897
21. Celmer N, Branaghan R, Chiou E. Trust in branded autonomous vehicles & performance expectations: A theoretical framework. In: Proceedings of the Human Factors and Ergonomics Society Annual Meeting. 2018; 62(1): 1761-1765. doi: https://doi.org/10.1177/154193121862139
22. Chinomona R, Sandada M. Customer satisfaction, trust and loyalty as predictors of customer re-purchase intention in south african retailing industry. Mediterranean Journal of Social Sciences. 2013;4(14): 437-446. doi: 10.5901/mjss.2013.v4n14p437
23. Hong IB, Cho H. The impact of consumer trust on attitudinal loyalty and purchase intentions in B2C e-marketplaces: Intermediary trust vs. seller trust. International Journal of Information Management. 2011;31(5): 469-479. doi: https://doi.org/10.1016/j.ijinfomgt.2011.02.001
24. Zhao JD, Huang JS, Su S. The effects of trust on consumers' continuous purchase intentions in C2C social commerce: A trust transfer perspective. Journal of Retailing and Consumer Services. 2019; 50(2): 42-49. doi: https://doi.org/10.1016/j.jretconser.2019.04.014
25. Chang HH, Su WC. The impact of online store environment cues on purchase intention: Trust and perceived risk as a mediator. Online Information Review. 2008;32(6): 818-841. doi: 10.1108/14684520810923953
26. Grant M. Culture and consumption: A theoretical account of the structure and movement of the

cultural meaning of consumer goods. Journal of Consumer Research, 1986;13(1): 71-84. doi: https://doi.org/10.1086/209048

27. Kennedy MS, Ferrell LK, LeClair DT. Consumers' trust of salesperson and manufacturer: An empirical study. Journal of Business Research. 2001;51(1): 73-86. doi: https://doi.org/10.1016/S0148-2963(99)00039-9

28. Halim P, Swasto B, Hamid D, Firdaus MR. The influence of product quality, brand image, and quality of service to customer trust and implication on customer loyalty (Survey on customer brand sharp electronics product at the south kalimantan province). European Journal of Business and Management. 2014;6(29): 159-166.

29. Ferrell OC, Gresham LG, Fraedrich J. A synthesis of ethical decision making models for marketing. Journal of Macromark. 1989;9(2): 55–64. doi: https://doi.org/10.1177/027614678900900207

30. Luu TT. The linkages among leadership, trust, and business ethics. Social Responsibility Journal. 2012;8(1): 133-148.

31. Lagace RR, Dahlstrom R, Gassenheimer JB. The relevance of ethical salesperson behavior on relationship quality: The pharmaceutical industry. Journal of Personal Selling and Sales Management. 1991;11(4): 39-47.

32. Goldsmith RE, Lafferty BA, Newell SJ. The impact of corporate credibility and celebrity on consumer reaction to advertisements and brands. Journal of Advertising. 2000;29(3): 43-54. doi: https://doi.org/10.1080/00913367.2000.10673616

33. Earle TC, Cvetkovich G. Social trust: Toward a cosmopolitan Society. Westport, CT: Praeger;

1995.

34. McAllister DJ. Affect- and cognition-based trust as foundations for interpersonal cooperation in organizations. The Academy of Management Journal. 1995;38(1): 24–59. doi: https://doi.org/10.2307/256727

35. Lewis JD, Weigert A. Trust as a social reality. Social Forces. 1985;63(4): 967-985. doi: https://doi.org/10.2307/2578601

36. Madsen M, Gregor S. Measuring human-computer trust. In: 11th Australasian Conference on Information Systems. 2000;53: 6-8.

37. Hatai S. The structure of the relationship between consumers and makers and the development of the measurement scale. Advances in Consumer Studies. 2004;10(1-2): 17-41. (In Japanese)

38. Kline RB. Principles and practice of structural equation modeling. 4th ed. New York: Guilford Press; 2010.

39. Hair JF, Black W, Babin BJ, Anderson RE. Multivariate data analysis: A global perspective. 7th ed. New York: Pearson Prentice; 2010.

40. Chin WW. The partial least squares approach to structural equation modeling. In: G. A. Marcoulides (Ed.), Modern Methods for Business Research. 1998;295–358.

41. Dikmenli M. Biology student teachers' conceptual frameworks regarding biodiversity. Education. 2010a;130(3): 479-489.

42. Dikmenli M. Biology students' conceptual structures regarding global warming. Energy Education Science and Technology Part B: Social and Educational Studies. 2010b;2: 21-38.

43. Kurt H, Ekici G, Aktas M, Aksu O. Determining biology student teachers' cognitive structure on

the concept of "diffusion" through the free word-association test and the drawing-writing technique. International Education Studies. 2013;6(9): 187-206. doi: 10.5539/ies.v6n9p187

44. Türkkan E. Investigation of physics teacher candidates' cognitive structures about "electric field": A free word association test study. Journal of Education and Training Studies. 2017;5(11): 146-156. doi: https://doi.org/10.11114/jets.v5i11.2683

45. Erdogan A. Examining pre-service mathematics teachers' conceptual structures about "geometry." Journal of Education and Practice. 2017;8(27): 65-74.

46. Bahar M, Johnstone AH, Sutcliffe RG. Investigation of students' cognitive structure in elementary genetics through word association tests. Journal of Biological Education. 1999;33(3): 134-141. doi: https://doi.org/10.1080/00219266.1999.9655653

47. Miles MB, Huberman AM. Qualitative data analysis: An expanded sourcebook. 2nd ed. Thousand Oaks, CA: SAGE Publications; 1994.

48. Aiken L. Psychological testing and assessment. 3rd ed. Boston: Allyn and Bacon; 1979.

49. Lee J, Abe G, Sato K, Itoh M. Developing human-machine trust: Impacts of prior instruction and automation failure on driver trust in partially automated vehicles. Transportation Research Part F: Traffic Psychology and Behaviour. 2021;81: 384–395. doi: https://doi.org/10.1016/j.trf.2021.06.013

50. Luhmann N, Poggi G, Burns T, Davis H, Raffan J, Rooney K. Trust and power: two works. U-M-I Out of Print Books on Demand; 1979.

51. Gefen D. E-commerce: the role of familiarity and trust. Omega. 2000;28(6): 725-737. doi: https://doi.org/10.1016/S0305-0483(00)00021-9

52. Zablotska IB, Grulich AE, de Wit J, Prestage G. Casual sexual encounters among gay men: familiarity, trust and unprotected anal intercourse. AIDS and Behavior. 2011;15(3): 607-612. Doi: 10.1007/s10461-010-9675-2 PMID: 20376696

53. Ha H, Perks H. Effects of consumer perceptions of brand experience on the web: Brand familiarity, satisfaction and brand trust. Journal of Consumer Behaviour. 2005;4(6): 438-452. doi: 10.1002/cb.29

54. Lafferty BA, Goldsmith RE. How influential are corporate credibility and endorser attractiveness when innovators react to advertisements for a new high-technology product? Corporate Reputation Review. 2004;7(1): 24-36. doi: https://doi.org/10.1057/palgrave.crr.1540209

55. Bhatt N, Jayswal R, Patel JD. Impact of celebrity endorser's source credibility on attitude towards advertisements and brands. South Asian Journal of Management. 2013;20(4): 74-95.

56. Sung Y, Kim J. Effects of brand personality on brand trust and brand affect. Psychology & Marketing. 2010; 27(7): 639-661. doi: https://doi.org/10.1002/mar.20349
